# Evidence for an upper cretaceous strandline deposit in the Lance Formation, Wyoming, USA

**DOI:** 10.1371/journal.pone.0362687

**Published:** 2026-11-12

**Authors:** Keith Snyder, Nelson Llempen, Caleb Darling, Alessandro Franchini, Matthew McLain, Arthur Chadwick

**Affiliations:** 1 Department of Biology and Allied Health, Southern Adventist University, Collegedale, Tennessee, United States of America; 2 Department of Earth and Biological Sciences, Loma Linda University, Loma Linda, California, United States of America; 3 Dinosaur Science Museum and Research Center, Southwestern Adventist University, Keene, Texas, United States of America; 4 Department of Biological and Physical Sciences, The Master’s University, Santa Clarita, California, United States of America; Indian Institute of Technology (IIT) - Bombay, INDIA

## Abstract

A series of apparently related quarries in the Maastrichtian (uppermost Cretaceous) Lance Formation of Wyoming provided the opportunity to evaluate the taphonomic mode for a suite of vertebrate fossils. Extensive work on the Hanson Ranch over the past three decades has exposed tens of thousands of fossils from dinosaur-bearing bonebeds. All of these bonebeds consist of disarticulated and disassociated remains. However, seven recently discovered quarries have yielded the remains of one or more dinosaur taxa (e.g., *Triceratops, Edmontosaurus, Thescelosaurus, Anzu)* in a state of association that was hitherto unknown on the ranch. These seven quarries were found along a line heading approximately 140 degrees, a heading reflected in the long axes of many of the elongate bones in the quarries. The preservation of associated remains suggests that soft-tissue connections or integumentary containment persisted during the transport and burial. We conclude that the floating, encapsulated carcasses of these animals were stranded along a southeasterly trending shoreline of the Western Interior Seaway.

## Introduction

The Upper Cretaceous (Maastrichtian) Lance Formation of Wyoming has been a rich source of dinosaur fossils since they were first identified there in the late 19th century [1]. Originally designated as the “Ceratops beds” [2,3], or the “Triceratops beds” [4], the formation is particularly well known for its diverse assemblage of ceratopsian, hadrosaurid, theropod, and other vertebrate remains. Extensive excavation conducted on the Hanson Ranch in Niobrara County, Wyoming over the past three decades has produced tens of thousands of vertebrate fossils from a variety of localities in the Lance Formation. (Fig 1; [5]) Previous investigation focused primarily on a large hadrosaur-dominated bonebed in which disarticulated and dissociated bones predominated [5], and where *Triceratops* and other taxa were rarely encountered. During the process of exploration, 14 additional quarries have been excavated elsewhere on the Ranch. While these quarries have provided the opportunity to examine the broader context of the taphonomic milieu represented in these Lance beds and have included more remains from ceratopsids (see, for example, [6]), they have not revealed the kinds of associated or articulated fossils that are found elsewhere in the Lance Formation [7].

**Fig 1:**
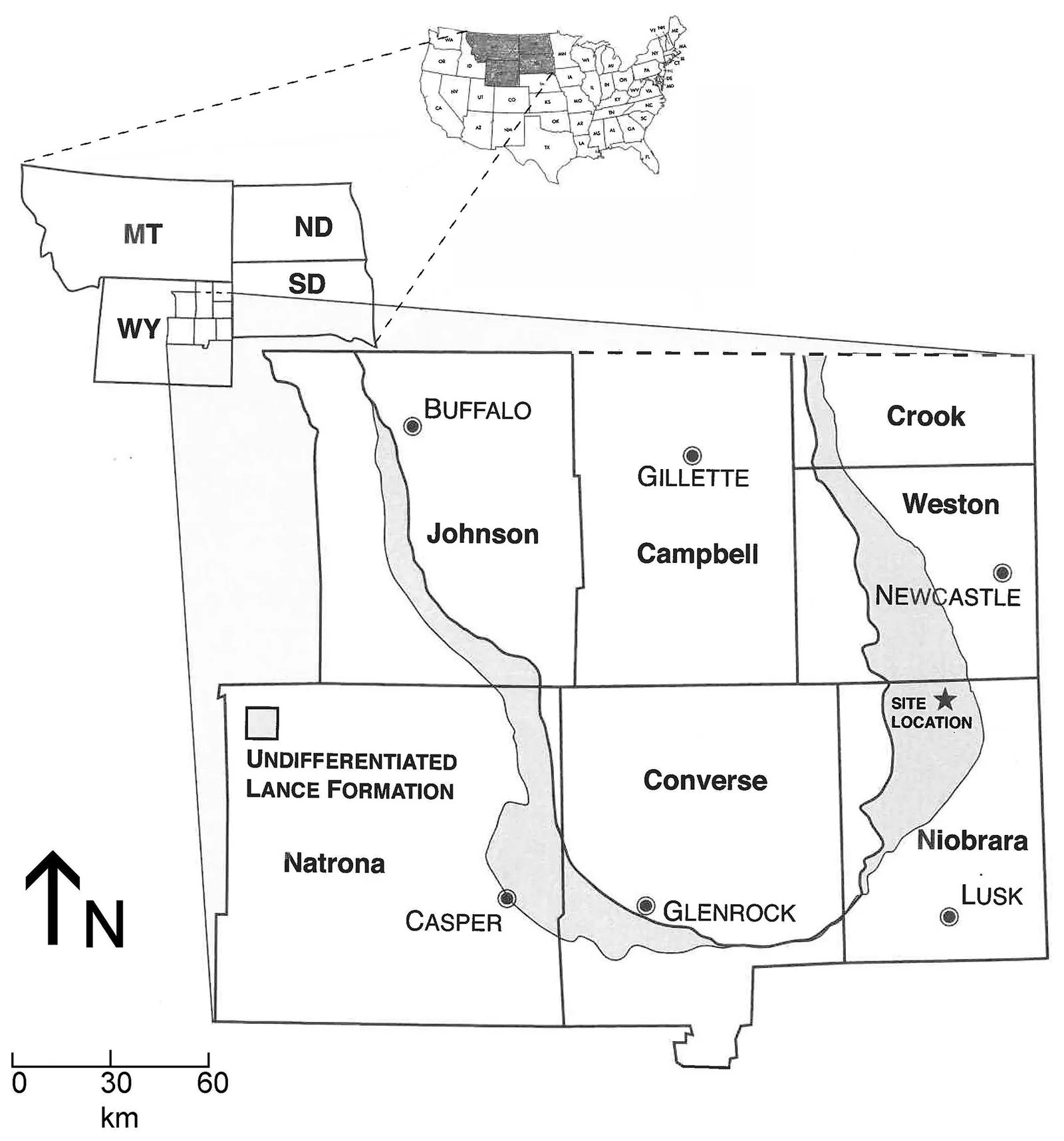
Study site locality in Niobrara County, Wyoming. Site indicated by star. Modified from Snyder, K. et al. 2020 [5]. Study sites are protected but exact location information will be shared with qualified researchers upon request.

A notable exception is represented by a series of recently identified quarries containing partially associated dinosaur skeletons. These sites preserve remains attributable to several taxa, including *Edmontosaurus, Triceratops, Thescelosaurus* and *Anzu*. In contrast to the more common dissociated bonebeds elsewhere on the Ranch, these sites contain skeletal remains that retained varying degrees of association during transportation and burial. The different modes of preservation reflected in these quarries provide an opportunity to investigate processes associated with carcass disarticulation, transport and deposition in greater detail and to expand understanding of the taphonomy represented in these beds.

The first of these sites, Triceratops2 Quarry, was discovered in 2014 when an inverted ceratopsian skull was exposed at the surface by erosion. Subsequent excavation exposed additional associated remains representing multiple dinosaur taxa within a relatively restricted area [8]. Continued exploration of nearby outcrops resulted in the discovery of six additional localities exhibiting similar taphonomic characteristics. Remarkably, these sites are distributed along a northwest-southeast trend extending approximately 3.25 km across the study area (Fig 2). The unusual preservation of associated but disarticulated remains across multiple localities, the consistent spatial alignment of the quarries, and the recurring patterns of bone orientation within the quarries raise important questions regarding the depositional processes responsible for their preservation. The present study documents the taphonomic characteristics of these quarries, evaluates their stratigraphic relationships and examines the possible depositional models capable of explaining their distribution. Particular attention is given to the extent to which the observed pattern may reflect deposition near the retreating shoreline of the Western Interior Seaway (WIS) in the latest Cretaceous.

**Fig 2:**
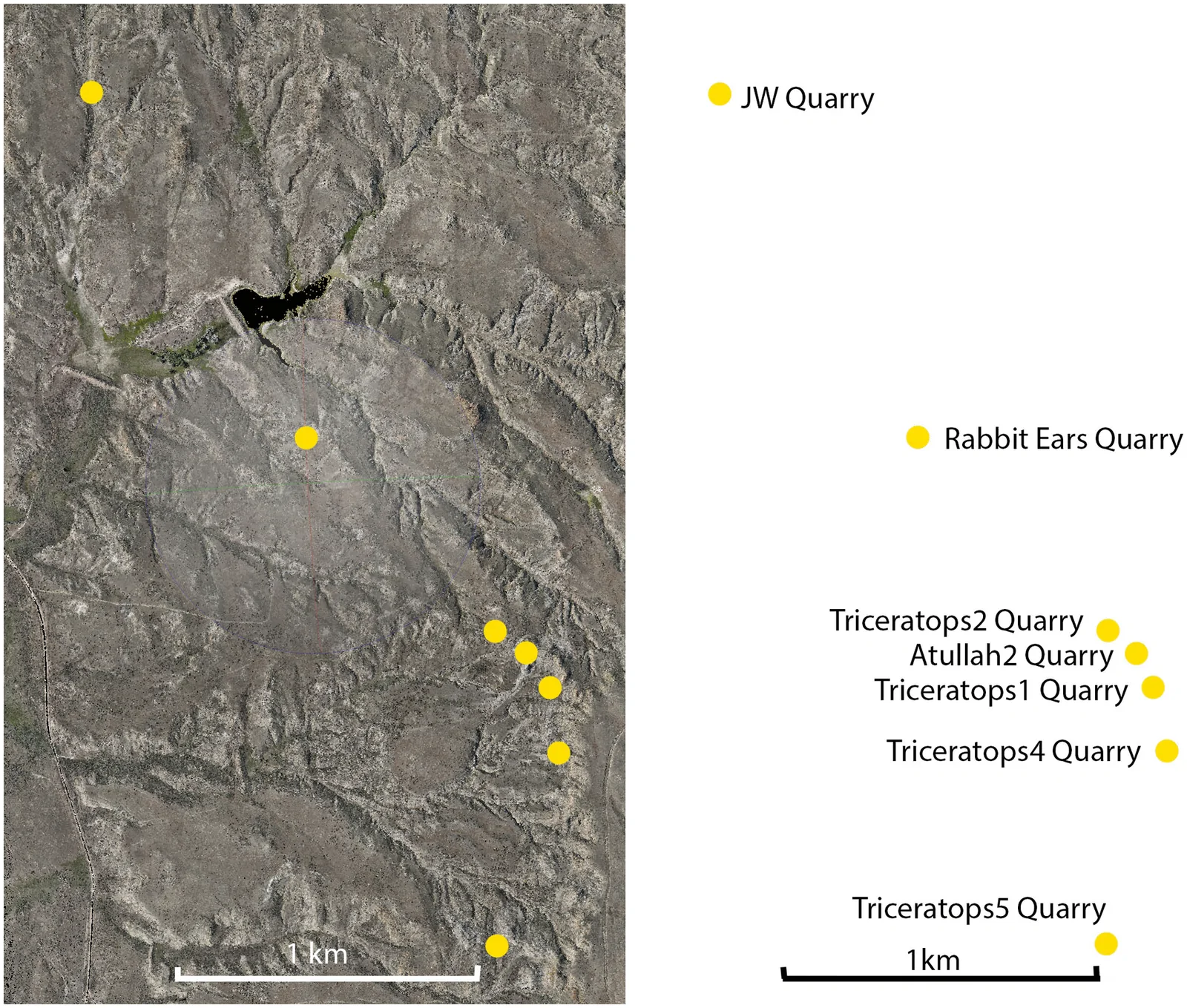
The seven quarries define an approximately NW-SE trend near 140-degrees. A) Quarries placed on a plan image of the area B) Quarries with background removed for visibility. North is up. Drone image produced by A. Chadwick.

### Geological setting

The study area is located within exposures of the Upper Cretaceous (Maastrichtian) Lance Formation on the Hanson Ranch in Niobrara and Weston Counties in eastern Wyoming. The Lance Formation represents one of the uppermost sedimentary units deposited during the final regression of the WIS. This formation is not only known for its dinosaurs but also preserves a wide variety of terrestrial and aquatic vertebrates. Lithologies exposed in the study area consist of poorly consolidated mudstones, siltstones, claystones, and fine-grained sandstones with locally resistant carbonate cemented sandstone units. Regional interpretations have traditionally viewed the Lance Formation as the product of fluvial systems occupying a broad coastal plain during the retreat of the WIS [9]. However, increasing sedimentological and stratigraphic evidence suggests that depositional environments within the formation were spatially heterogeneous and probably included a range of fluvial, deltaic, estuarine and marginal marine settings. Consequently, individual localities may record substantially different depositional conditions despite their inclusion within the same lithostratigraphic unit.

The Hanson Ranch locality occupies a position near the eastern margin of the Maastrichtian foreland basin and lies within a region that doubtless would have experienced shoreline migration during the regression of the WIS (Fig 3). Paleogeographic reconstructions indicate that shoreline positions shifted progressively eastward during the uppermost Maastrichtian [10,11]. As a result, depositional environments represented within the Lance Formation in eastern Wyoming would likely have changed as coastal, nearshore and terrestrial settings migrated eastward.

**Fig 3:**
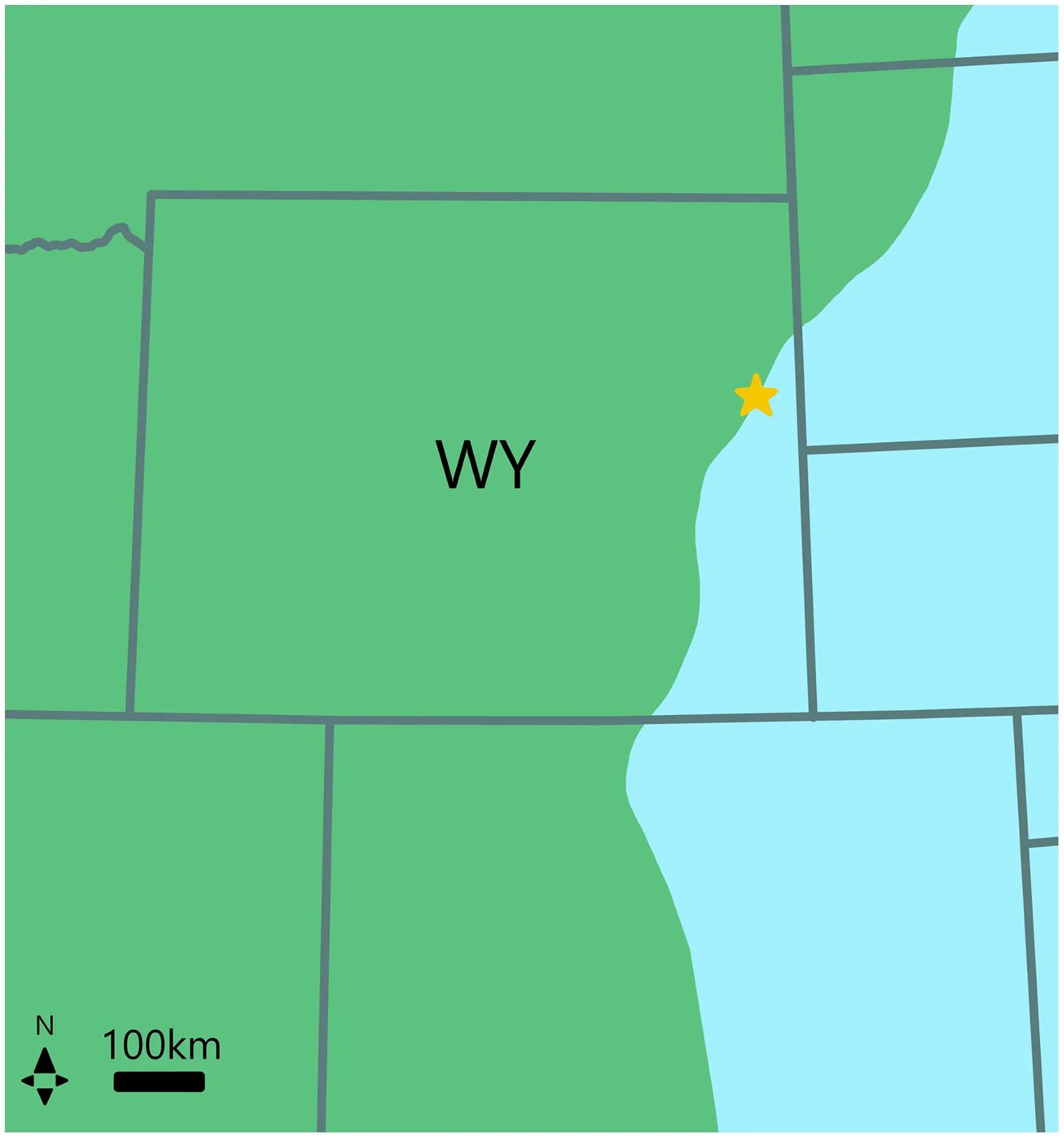
Paleogeographic map of the Upper Maastrichtian western shoreline of the WIS.

Measured sections from the study area revealed a succession dominated by purple mudstones, gray siltstones and fine-grained white sandstones. Several laterally persistent carbonate-cemented sandstone units provided useful marker horizons for local stratigraphic correlation. Petrographic examination of selected sandstone beds identified quartz grains, volcanic and plutonic lithic fragments, secondary carbonate cement and glauconitic material. The occurrence of detrital and glauconitic cement is consistent with marine influence and with other data suggesting that at least part of the Lance in this area was deposited offshore [5,12]. Cobban et al. [13] identified the lower Maastrichtian shoreline as being to the west of this locality. Although the precise temporal pattern of shoreline migration in northeastern Wyoming as preserved in uppermost Maastrichtian deposits is poorly known, it is generally acknowledged that the WIS was receding to the east and the shoreline would have migrated across this location during deposition of the Lance Formation (Fig 3 [10,11],). If this view is true, then somewhere west of the main quarry area, which is deposited offshore [5], a contemporary strandline deposit would be anticipated.

Based upon data from Roberts and Kirschbaum [10] and Lillegraven and Ostresh [11] illustrating the position of the quarries (star) described in this paper. Image produced by A. Franchini.

Because the assessment of outcrop continuity is limited by weathering and vegetation cover, long-distance stratigraphic correlations within the study area are tentative. Accordingly, the stratigraphic framework developed here is intended primarily as a working model for evaluating relationships between the various quarry sites and may not be applicable on a wider scale to the upper Lance Formation. The interpretations included here are evaluated in the context of both the available stratigraphic and sedimentological evidence and plausible alternative depositional models.

## Materials and methods

### Quarry excavation and documentation

The quarries that are the subject of this report are one kilometer west of the main Hanson Ranch bonebed previously described by Snyder et al. [5]. Excavation of the sites was conducted using centimeter scale Real-time Kinematic Global Positioning System (RTK-GPS) technology to document the positions of all recovered specimens [14]. Individual specimens were exposed, assigned field numbers, described, photographed and mapped prior to removal from the outcrop. Positional data were obtained from the upper surface of each specimen using RTK-GPS. Recovered specimens were stabilized in the field and transported to the Dinosaur Science Museum and Research Center (Keene, Texas) for preparation and curation. Spatial data collected during excavation were combined with photographic data using Geographic Information System (GIS) software to generate virtual 3D maps of the quarries, illustrating the position of each bone in plan view [14]. These digital reconstructions served as a basis for subsequent taphonomic analysis. All specimen metadata, quarry maps and associated records are archived within the fossil database available through the Dinosaur Science Museum and Research Center repository (https://fossil.swau.edu). Click “Quarry” and then choose the appropriate quarry you wish to search.

### Stratigraphic analysis

A local stratigraphic framework was developed through measurement and description of sedimentary sections adjacent to each quarry locality. Lithologic descriptions included grain size, sedimentary structures, color, bed thickness, cementation characteristics, paleocurrent directions and fossil content. Elevation control for all measured sections was established using RTK-GPS methods [14,15].

Correlation of individual sections was based primarily on laterally traceable lithologic marker beds, including resistant carbonate-cemented sandstones and distinctive mudstone intervals. Correlation confidence varies among localities and is discussed in context where relevant.

### SEM methodology

Scanning Electron Microscopy (SEM) was used to analyze samples from a resistant sandstone at the bottom of Cycle 1. The samples were mounted with double sided carbon tape. A sputter coater was then used to coat the samples in a gold-palladium coating. The samples were then analyzed using a VEGA II LSH Scanning Electron Microscope. The imaging mode used for this analysis was secondary electron (SE) imaging. The analysis was run with an accelerating voltage of 10kV, a beam current of 77.600nA, and 3.78k x magnification.

### XRD methods

A bulk sample XRD was run on a resistant sandstone sample from the bottom of Cycle 1. The samples were prepared by crushing with a stainless-steel mortar and pestle. Two milliliters of the sample were then further ground to < 10μm by mechanical milling using a McCrone micronizing mill. The samples were air-dried over a period of several days before being analyzed. The samples were analyzed using a Bruker D8 Advance X-ray Diffractometer and indexed using JADE. The X-ray radiation source on the Bruker D8 Advance is a Cu long, fine focus X-ray tube, emitting Cu Ka radiation. The analysis was run with 40kV voltage, 40mA current, and a scanning range (2θ) or 2.00°–72.98°.

### Taphonomic analysis

Specimens were assigned to taxonomic categories based on diagnostic anatomical features. Skeletal association, articulation, completeness, element representation and spatial distribution were documented in each quarry. Particular attention was given to evaluating the degree of skeletal association preserved during burial. Quarry maps were used to reconstruct the distribution of skeletal elements and to assess whether preserved remains likely originated from individual carcasses.

### Orientation analysis

Orientation measurements were collected for all elongate skeletal elements exhibiting a length-to-width ratio (L/W) greater than 2:1. Long axis orientations were measured from digital quarry diagrams and recorded as axial data. Measurements were grouped into 15° classes and analyzed using circular statistical methods. Because axial data contain an inherent 180° ambiguity, orientations were transformed using the standard double-angle procedure prior to application of the Rayleigh tests for preferred orientation [16]. Also, because axial data give direction but do not independently establish the sense of transport, paleocurrent data from Brunton compass measurements on cross-beds, ripples, and trough axes in the vicinities of the quarries were compiled and used to aid in interpretation of sense of flow (https://doi.org/10.5061/dryad.gtht76j3s).

### Interpretive framework

The primary objective of this study was to evaluate the depositional processes responsible for the preservation of associated dinosaur remains across multiple quarries. Taphonomic observations, bone orientation data, stratigraphic relationships, sedimentological characteristics and regional paleogeographic reconstructions were all considered together to evaluate competing depositional hypotheses, including fluvial, deltaic and shoreline related accumulation models.

No permits were required for the described study, which complied with all relevant regulations. Contracts between the landowners and Southwestern Adventist University are on file at the university.

## Results

### Stratigraphy

This portion of the Lance Formation reflects depositional conditions near the eastern shoreline of the Western Interior Seaway during the uppermost Cretaceous [5]. Although stratigraphic resolution in the Lance is often hindered by weathering and limited outcrop continuity, three regionally correlative sedimentary cycles were identified—designated Cycle 1(C1), Cycle 2(C2), and Cycle 3(C3)—which can be consistently traced across all quarries (Fig 4) constrained by continuous red carbonate cemented sandstones.

**Fig 4:**
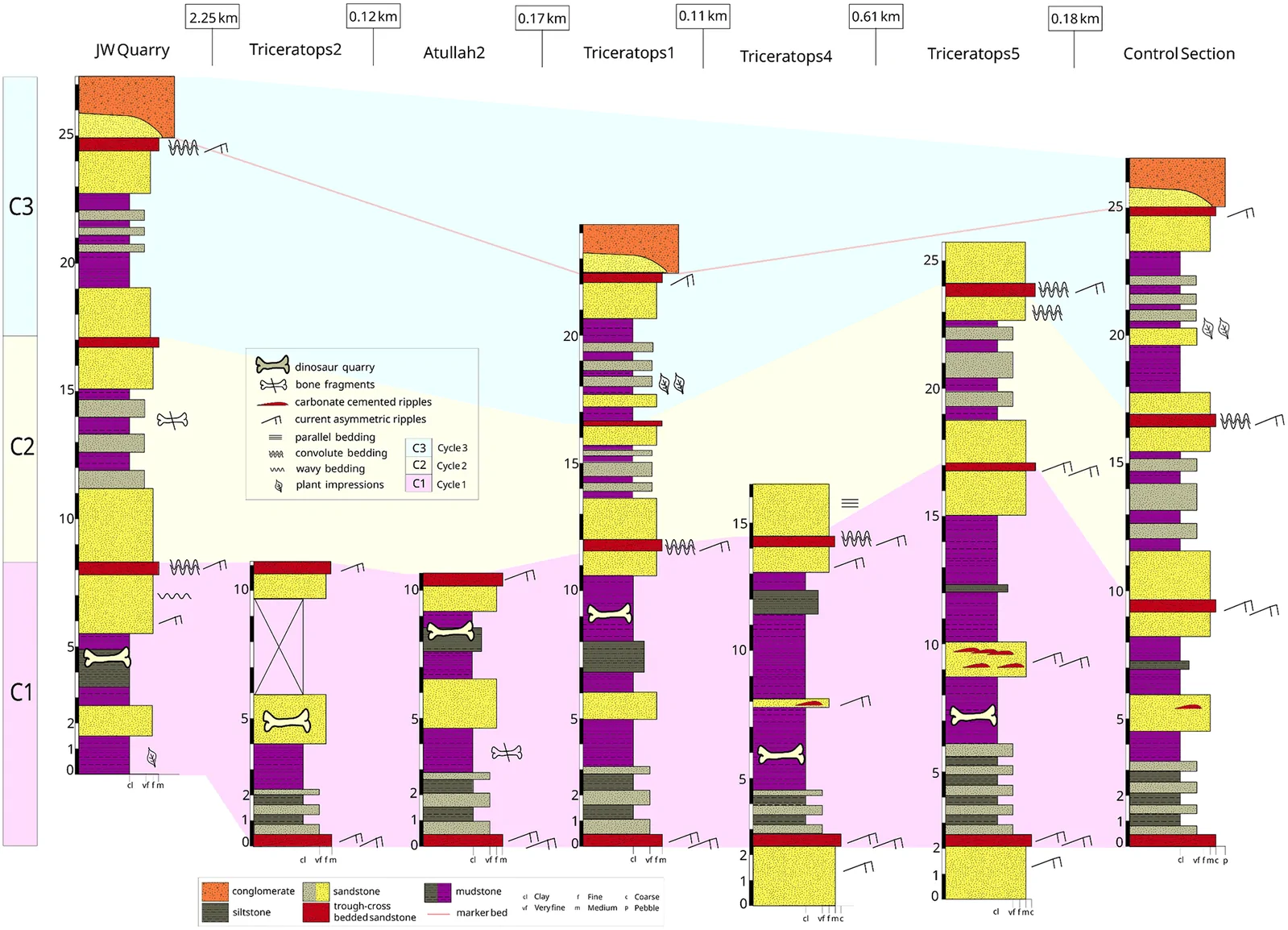
Stratigraphic sections taken near six of the quarries, and a control section taken nearby. The fossil accumulations appear at several horizons within a single cycle.

#### Cycle 1.

Cycle 1 forms the basal part of all measured sections and is characterized by a dominance of purple mudstones and grey siltstones interbedded with thin to medium-bedded very fine- to coarse-grained sandstones. This cycle shows the highest number of preserved vertebrate fossil localities, with most bone accumulations embedded in purple massive mudstone facies particularly in Triceratops4 and Triceratops5 quarries, which most likely represent the same stratigraphic horizon. Occasionally, these sediments also contain fossil plant macerals. Similar stratigraphic alignment is observed between Triceratops1, Atullah2, and possibly JW Quarry, although this latter correlation remains tentative due to distance (3.25 km from Triceratops5) and lack of high-resolution control at this scale within the Lance Formation. A distinctive middle sandstone bed within Cycle 1, yellow in color and occasionally showing carbonate-cemented current ripples, can be correlated across multiple sections. This bed thins toward the southeast, appearing nearly absent at Triceratops4, and thickens again toward Triceratops5 and the Control Section. This stratigraphic behavior suggests lateral wedging, potentially related to channel migration or bar accretion in a fluvial to deltaic setting. Notably, Triceratops2 Quarry is the only site whose fossil remains were hosted within this sandstone rather than in the bounding purple mudstones. The correlations of Triceratops1, Attulah2, Triceratops4, and Triceratops5 are viewed with high confidence, as the units were walked out along a continuous 1.5 km exposure containing these four quarries. However, due to a fault identified between Attulah2 and Triceratops2, and the discontinuity of outcrop between the exposure and JW quarry, the correlations of these two quarries are viewed with lower confidence.

Below Cycle 1, similar facies assemblages—mudstones interbedded with ripple-laminated sandstones—persist, suggesting continuity in depositional processes prior to the base of the measured section. For a proper analysis of this main cycle and all sections, the first laterally consistent red cemented sandstone was arbitrarily selected as a sequence base level. Petrographic analysis of a resistant sandstone at the bottom of Cycle 1 revealed plutonic and volcanic rock fragments, suggesting input from a terrestrial source to the west. The samples were cemented by secondary carbonate cement. The samples also contained glauconite in the forms of cement and coating on quartz grains (Fig 5). SEM imaging also revealed peloidal glauconite containing mixed glauconite and smectite, which indicates early stages of glauconitization (Fig 6). Bulk sample XRD also yielded results consistent with the identification of interlayered glauconite/smectite with a 10Å peak at approximated 9^º^, and a low intensity peak at 6.5^º^ (Fig 7, [17]). The presence of early stage glauconitization as well as the glauconite being present as cement and grain coatings seem to indicate that the glauconite present in this sample is of authigenic origin [18]. This evidence signifies a marine influence [18,19]. These factors along with regional paleogeographic reconstructions (Fig 4), have allowed consideration of a marginal marine environment for the deposition of Cycle 1.

**Fig 5:**
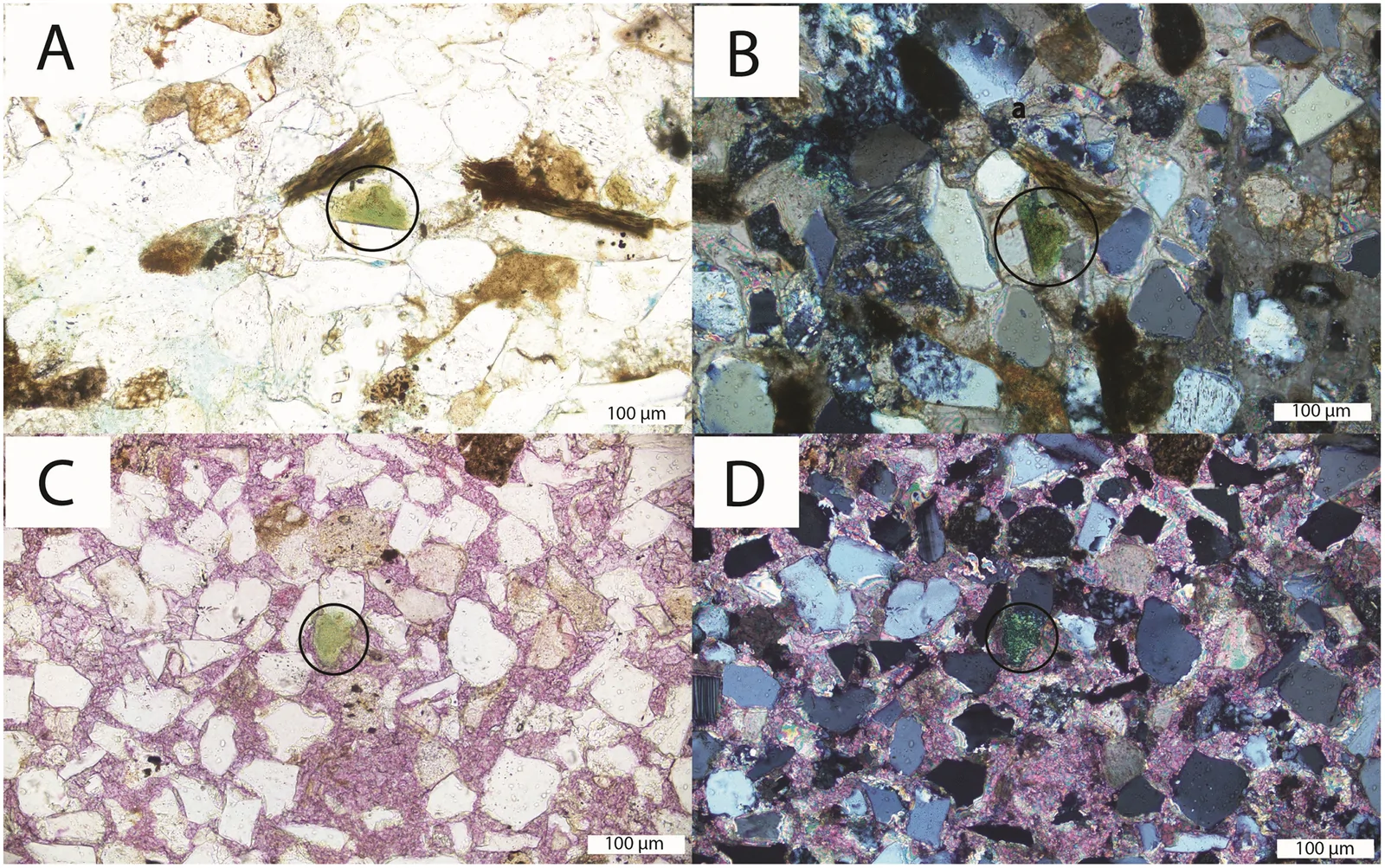
Thin section of resistant sandstone below Cycle I. Authigenic glauconite and glauconite cement infilling between detrital grains, (A) plane polarized light and (B) cross-polars. Glauconite coating on detrital quartz grains, (C) plane polarized light and (D) cross-polars. Prepared by National Petrographic Service, Inc. and photographed with an Olympus BX53-P petrographic microscope, 20x objective.

**Fig 6:**
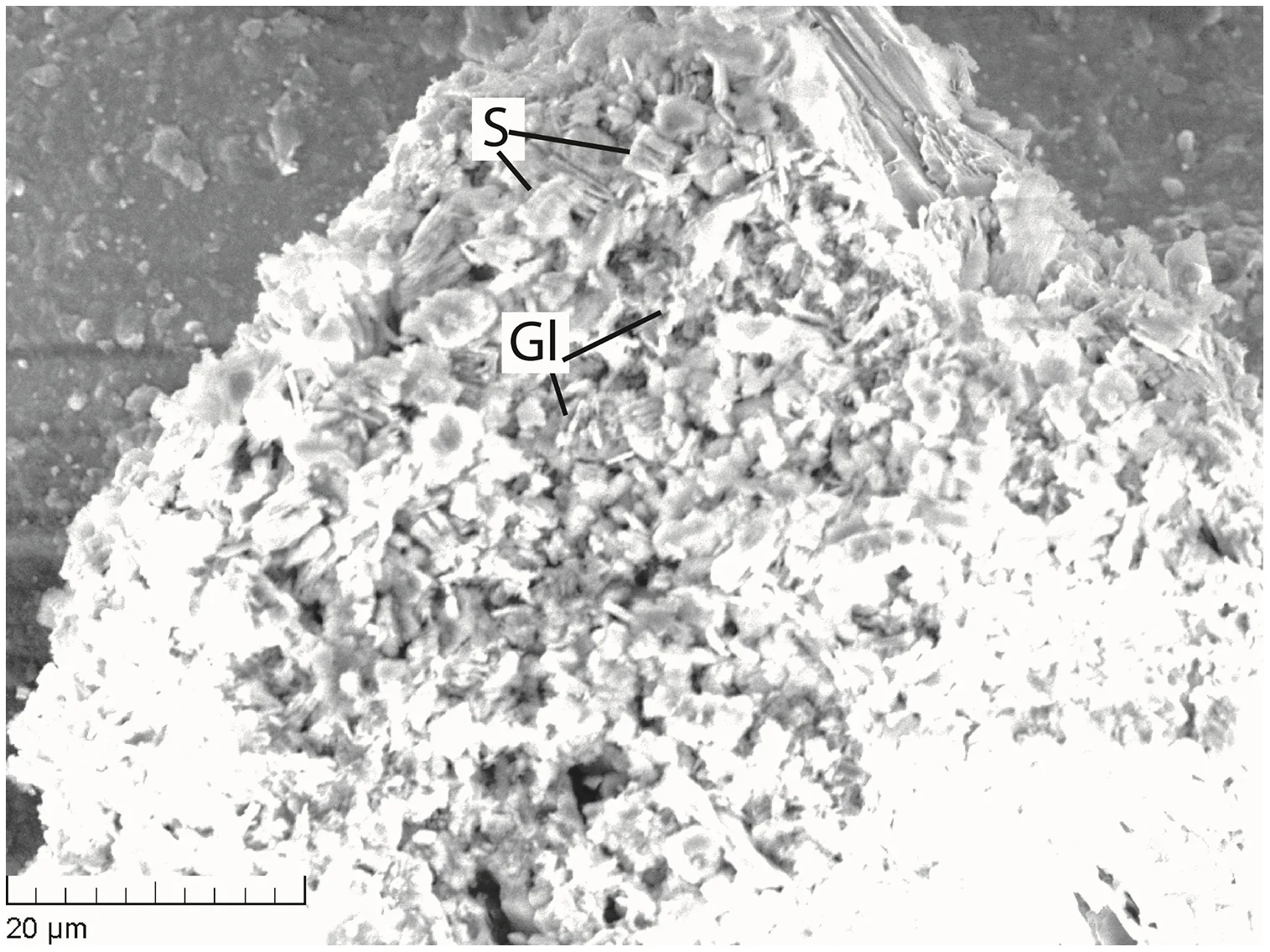
SEM imaging of resistant sandstone at the bottom of Cycle 1. Image of glauconite pellet with mixed glauconite (Gl)/smectite (S).

**Fig 7:**
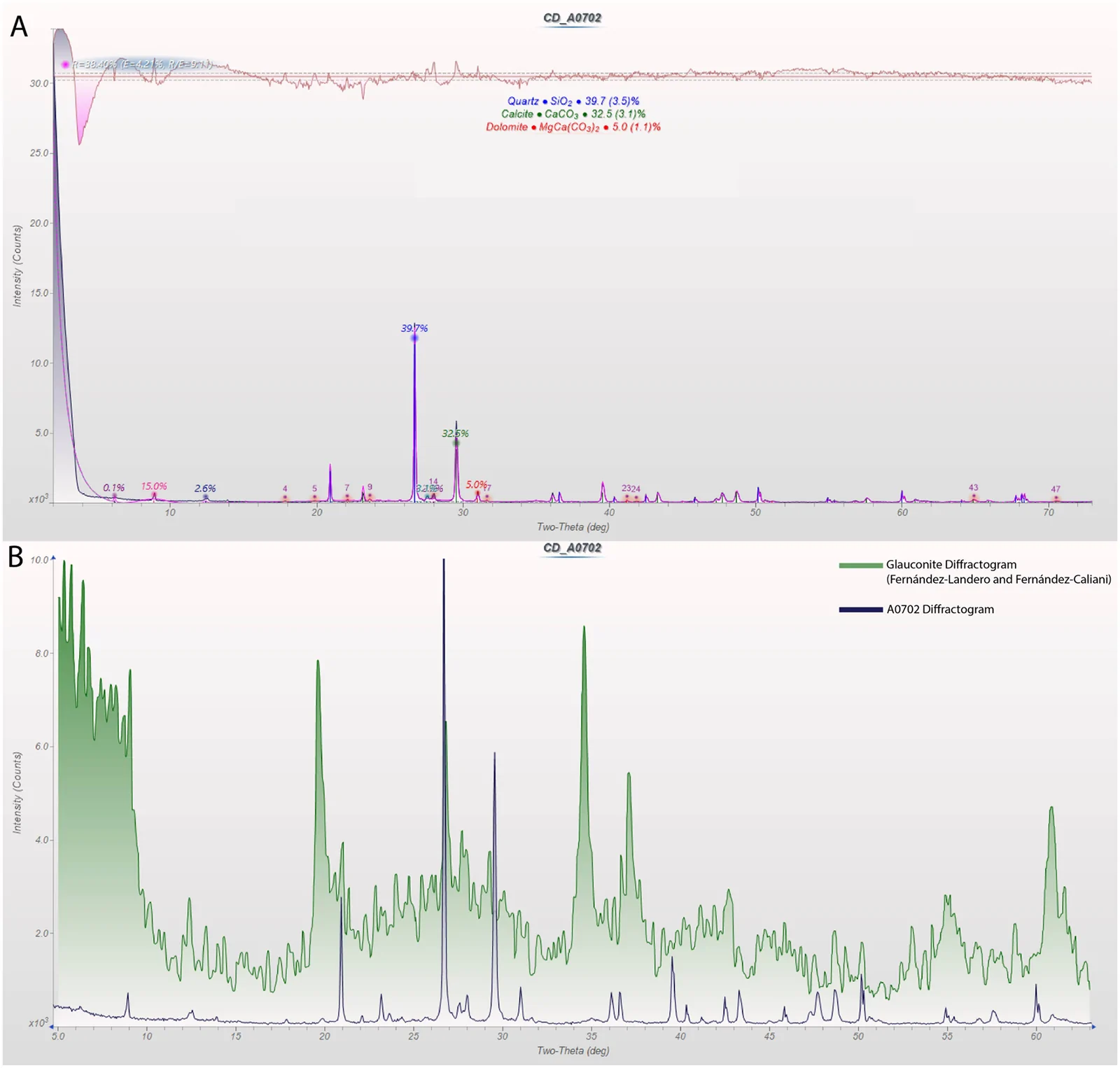
Bulk XRD results from resistant sandstone at the bottom of cycle 1. (A) Generalized indexing of diffractogram. (B) Diffractogram overlaid with glauconite separate diffractogram [17].

#### Cycle 2.

Unlike Cycle 1, Cycle 2 lacks any known fossil quarries, but the beds retain value for correlation since they exhibit little lateral facies change and moderate thickness variations. Cycle 2 is composed of interbedded very fine- to medium-grained sandstones with parallel laminations, and reduced percentage of purple mudstones and thinner bedding overall. Scattered dinosaur bone fragments were occasionally encountered in the mudstones. These facies may represent higher-energy overbank or levee deposits.

#### Cycle 3.

Cycle 3, like Cycle 2, contains no known fossil quarries, but preserves the same lithological vertical facies arrangement, dominated by purple mudstones containing plant remains and very fine tan sandstones. This cycle is capped by a conglomeratic unit that serves as a reliable regional marker bed, easily distinguishable by its carbonate mud-clast-supported fabric, erosive base, and interbedded medium sandstone. This conglomerate is clearly exposed in Triceratops1, Triceratops2, and the Control Section, and has also been identified in smaller, mapped outcrops between them. This distinctive bed likely marks a critical shift toward higher-energy proximal shoreline migration. An additional notable feature throughout the whole succession, is the continuity of the carbonate-cemented, red-colored sandstones with trough crossbedding. Although the carbonate cementation likely reflects diagenetic overprinting, the lateral continuity, consistent stratigraphic position, and common association with soft-sediment deformation suggest these beds can be confidently used as secondary correlation markers, likely reflective of episodic events that establish a time constraint.

### Stratigraphic and depositional trends

Despite the lateral distance, these facies can be continuously correlated across all sections, with only thickness variations observed, implying minimal lateral facies change and supporting deposition in a relatively low-gradient, laterally extensive floodplain or interdistributary deltaic environment. These thickness differences may be the result of localized sedimentologic controls, such as point bar–floodplain interactions, channel avulsion, or crevasse splay progradation.

The vertical stacking of facies across the sections displays a tentative coarsening-upward trend, consistent with the increasing energy levels at the top of each cycle. This is reflected in the pattern of current ripple lamination to wavy bedding to convolute bedding, consistent with a pro-gradational depositional regime supporting gradual shoreline pro-gradation during regression of the WIS.

The stratigraphic correlation was constructed along a NW–SE transect, matching the taphonomic alignment of quarries and long bone orientations (~140° azimuth). Continuous stratigraphic tracing is difficult due to deep weathering, poor outcrop exposure, and limited resolution, particularly at Rabbit Ears Quarry, which remains unexplored. However, the well-preserved profiles invite consideration of a model with continuous sedimentary succession and strong lateral connectivity of facies, minimal facies change, and localized variations in bed thickness. Compiled paleocurrent data from 65 measurements on outcrops in the vicinity of these quarries yielded a mean direction of 141 degrees (r > 22, p < .005) shown in Fig 8.

**Fig 8:**
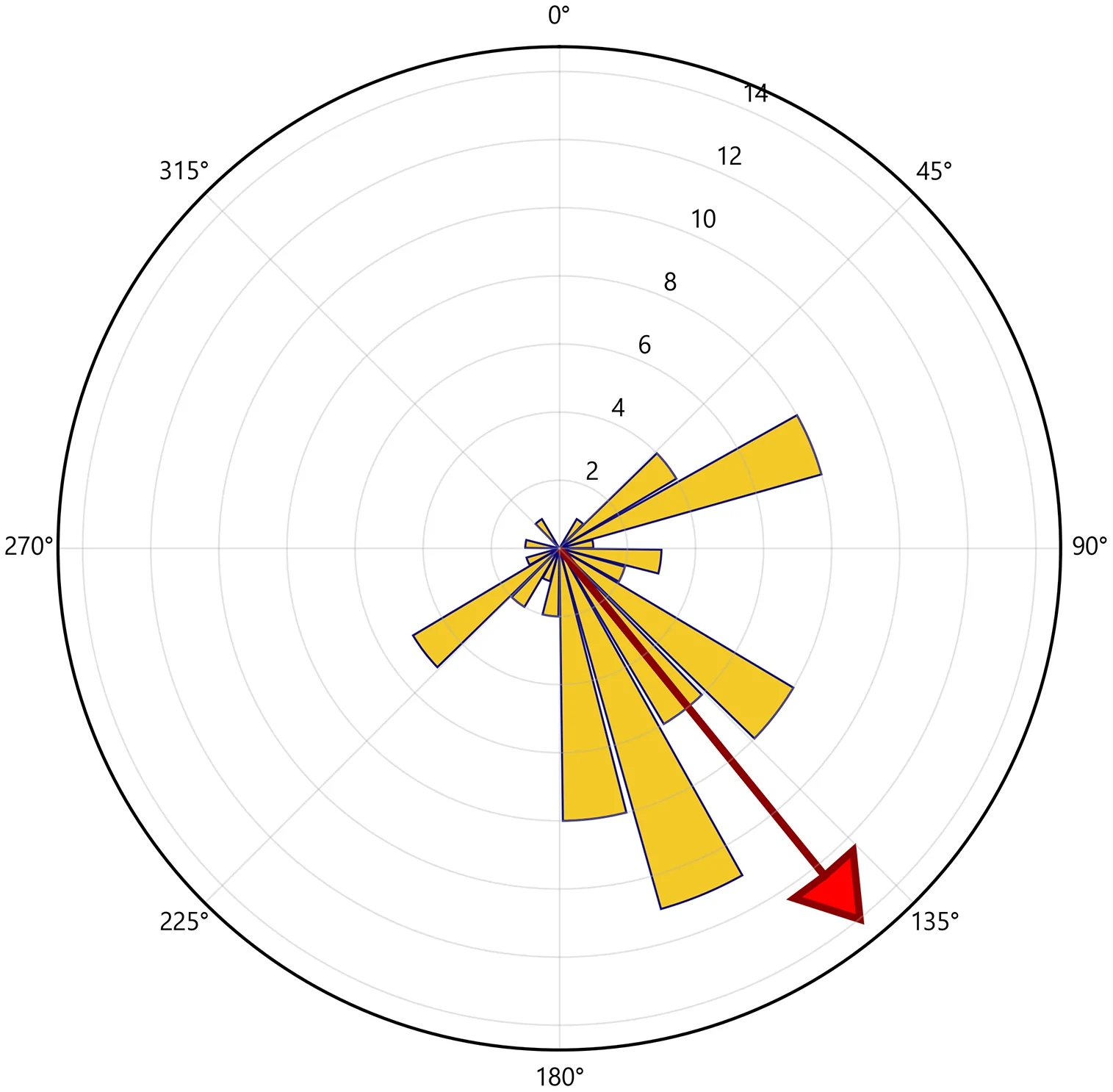
Paleocurrent rose derived from measurements on outcrops in close association with the quarries described in this paper. Number of measurements, 65; Rayleigh statistic, z = 22.12; P-value < .001; Mean direction 141.0 degrees. Arrow gives mean paleocurrent direction.

### Taphonomy

Triceratops2 Quarry – This quarry contained the remains of four distinct dinosaur individuals of four different taxa in various states of disarticulation and disassociation, in addition to other non-dinosaurian taxa (Fig 9). All bones with distinctively elongate axes (L/W > 2, in this case 80 specimens) were plotted in a rose diagram (Fig 10A). The general axes of elongate bones in this quarry were west and east, although the direction of dispersion of the bones in the quarry itself is northwest to southeast. The remains of each taxon are detailed below in separate diagrams (Fig 11). The elevations (in meters above sea level) of some bones are given in parentheses. These numbers are a mean of several readings taken across various points on each bone.

**Fig 9:**
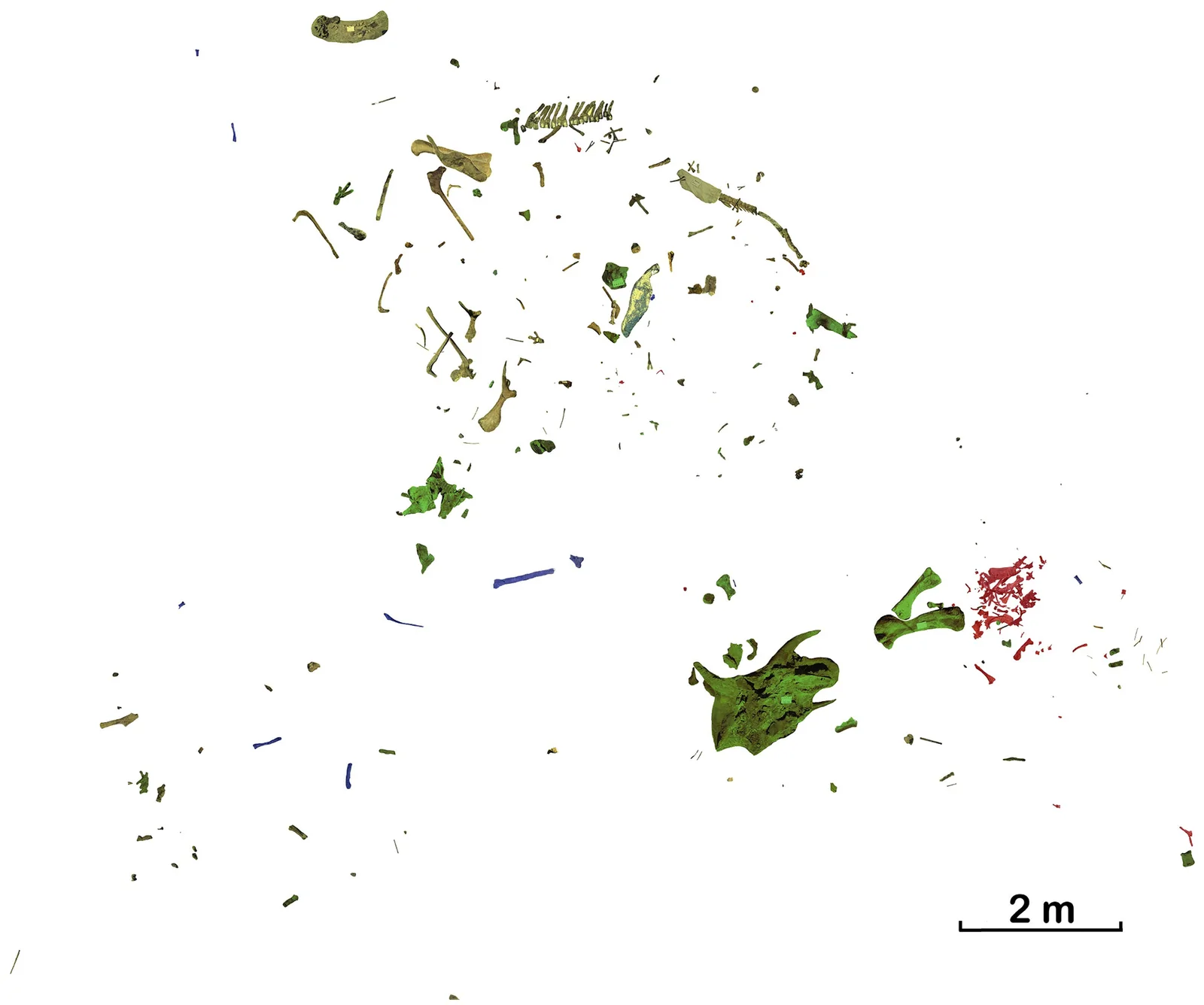
Quarry diagram for Triceratops2 Quarry with all bones represented. *Edmontosaurus* bones depicted in yellow, *Triceratops* bones in green, *Thescelosaurus* bones in red, *Anzu* bones in blue. North is up.

**Fig 10:**
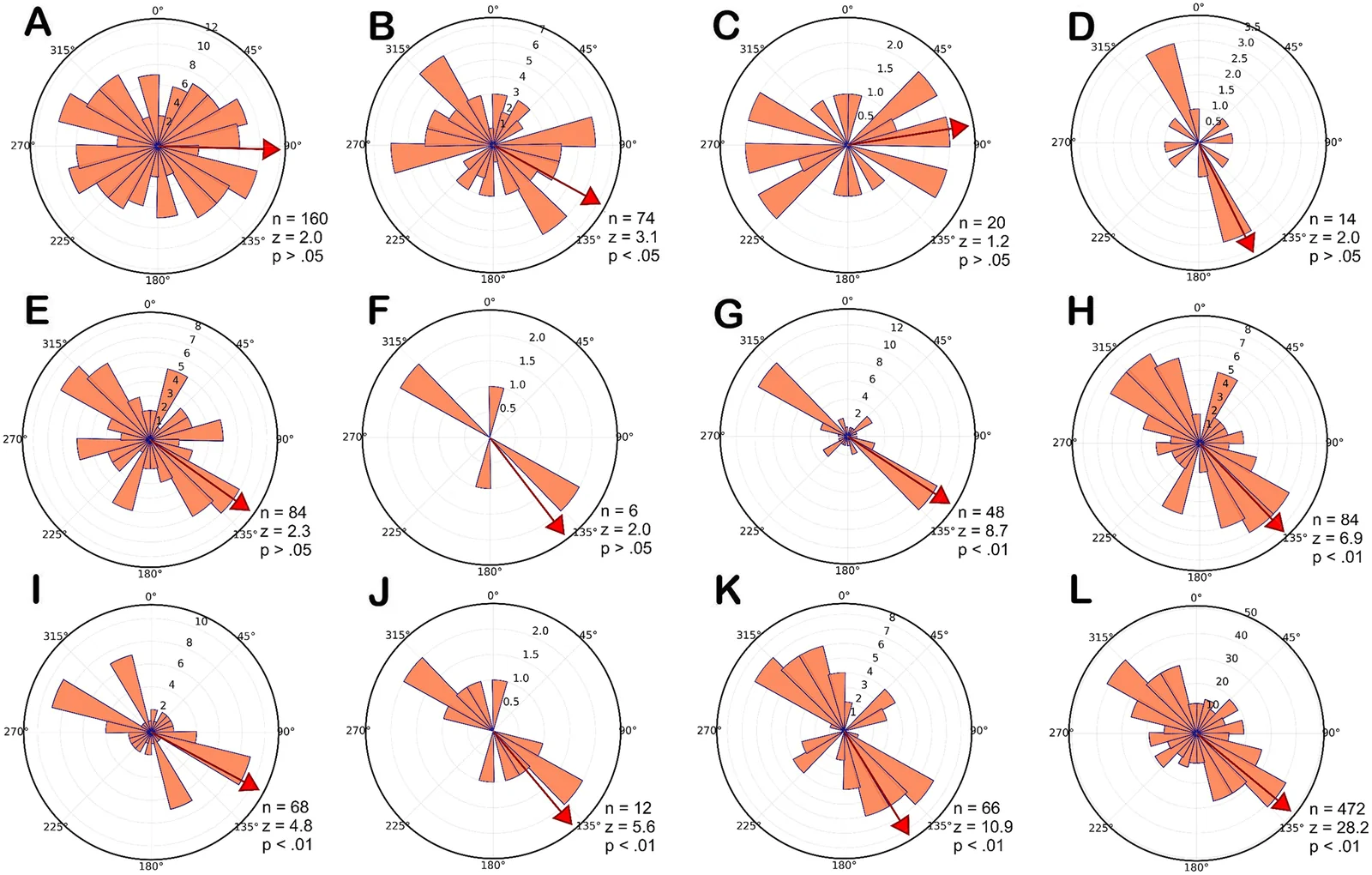
Rose diagrams for orientation of elongate bones in all quarries. A – E) Triceratops2 Quarry A) all bones. B) *Triceratops* bones. C) *Anzu* bones. D) *Thescelosaurus* bones. E) *Edmontosaurus* bones. F) Triceratops1 Quarry. G) Atullah2 Quarry. H) JW Quarry. I) Triceratops4 Quarry. J) Rabbit Ears Quarry. K) Triceratops5 Quarry. L) Composite of all quarry diagrams (B-K). N gives the number of measurements. Z gives the Rayleigh statistic. P gives the probability.

**Fig 11:**
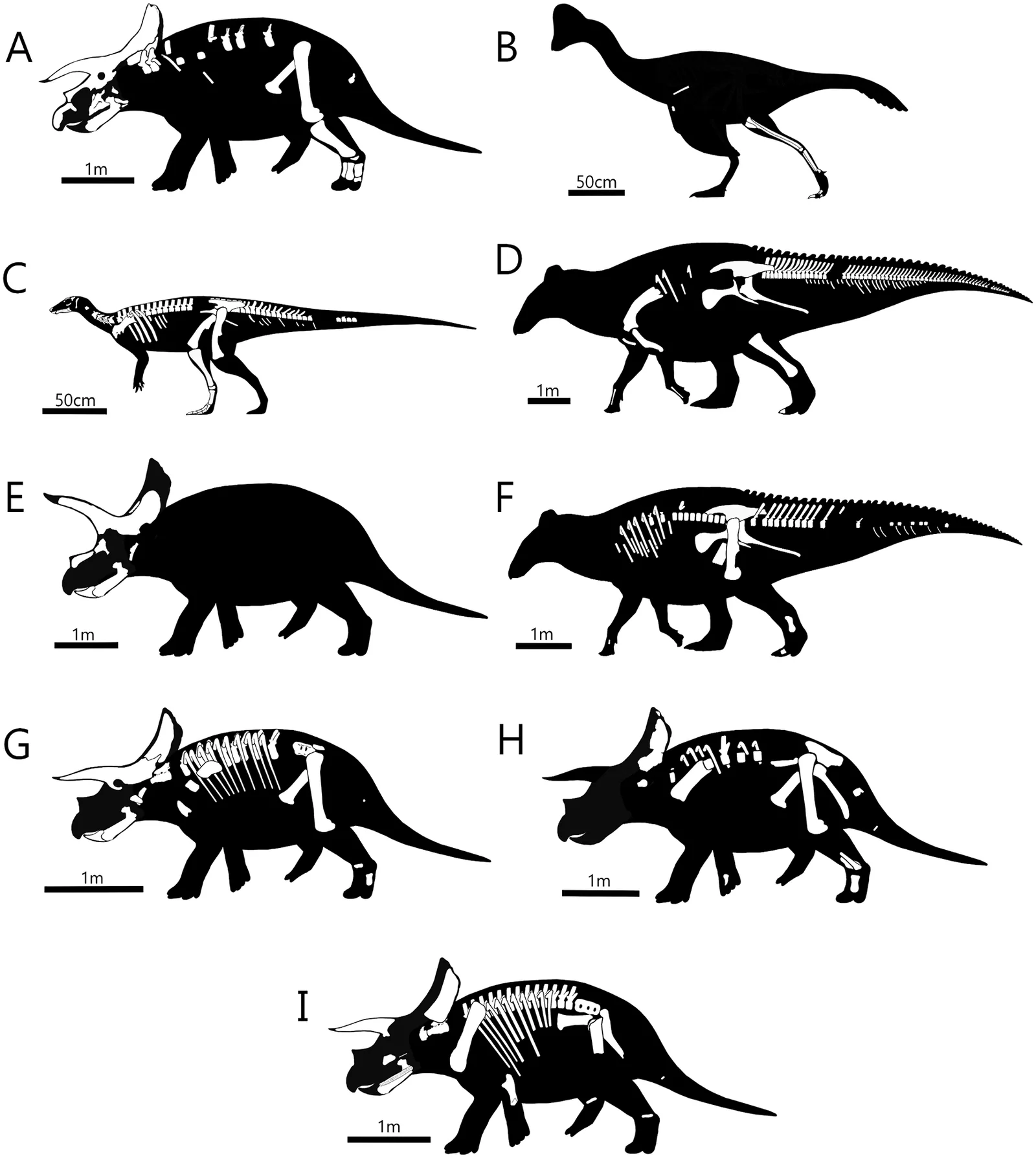
Dinosaur bones in skeletons recovered in each quarry. Left side illustrated. Triceratops2 Quarry A) *Triceratops*. B) *Anzu*. C) *Thescelosaurus*. D) *Edmonstosaurus*. E) Triceratops1 Quarry – *Triceratops*. F) Atullah2 Quarry – *Edmontosaurus*. G) JW Quarry – *Triceratops*. H) Triceratops4 Quarry – *Triceratops*. I) Triceratops5 Quarry – *Triceratops*.

#### Triceratops.

A mostly intact, large, chasmosaurine ceratopsid skull (HRS 15437) and associated postcrania are referred to the genus *Triceratops* based on their presence in the Lance Formation and the lack of fenestrae in the parietals (distinguishing it from *Torosaurus*). This specimen can be further referred to *Triceratops horridus* rather than *Triceratops prorsus* based on its shallow and long rostrum and its short nasal horncore [20]. The skull was found inverted with the brow horns pointing in a northeasterly direction (Fig 9, green bones bottom center, Fig 11A). The elevation of the lowest part of the skull was 1235.2 meters. The disarticulated left squamosal (1235.4) was above and in contact with the parietal region of the skull. The right squamosal (1235 .5) was displaced a meter to the south at the same elevation as the top of the skull. The right maxilla (1235.0) was located three meters northwest of the skull. The left maxilla (1235.4) was 0.3 meters to the east of the skull. The left dentary fragments (1235.5) were four meters due north of the skull. Neither the maxillae nor the dentary possessed any teeth. Two cervical, five dorsal and one caudal vertebrae were recovered (all between 1235.4 and 1235.7) spread out east-northeast of the skull (Fig 11). Only two partial ribs attributed to *Triceratops* were found, and none of the chevrons in the area were attributed to *Triceratops* since they were all associated with the *Edmontosaurus* tail mentioned below. A single *Triceratops* femur, tibia, pubis, and one metatarsal (all 1235.1), presumably from the same animal, were one to two meters northeast of the skull. These bones, like the skull itself, were deposited with long axes aligned with an east-northeast to west-southwest direction. A second metatarsal (1234.8) was a meter north of the skull and a third (1235.1) was three meters east of the skull. The trend of the 34 elongate bones attributable to *Triceratops* is generally along a west- northwest to east- southeast line (Fig 10B).

#### Anzu.

These remains are the first found on the Ranch unambiguously attributable to the caenagnathid oviraptorosaur, *Anzu wyliei* [21], as demonstrated by the rugosity in the middle surface of the astragalocalcaneum (HRS 15439), which is an autapomorphy of *A. wyliei* [22]. The astragalocalcaneum fits well on the tibia, and the fibula, metatarsals, and phalanges are all similarly-sized and not duplicated, suggesting these bones came from the same individual of *Anzu wyliei*. These remains will be described further in a future publication. No cranial material from *Anzu* has yet been recovered. Two caudal vertebrae were recovered, the first (1234.9) two meters north and the second (1235.2) 3.5 meters north of the *Triceratops* skull. A partial radius (1235.0) was 5.5 meters north and six meters west of the *Triceratops* skull and a partial metacarpal (1234.9) was one meter northwest of that. The largest bone from *Anzu* recovered was a tibia (1235.1) two meters west of the *Triceratops* skull (Fig 9, blue bones). At the same elevation and another meter west-southwest was the delicate but intact fibula. Two metatarsals (1235.0, 1235.1) were three meters south and six and five meters west from the skull. The astragalocalcaneum (1235.1) was 30 cm east of the tibia. A pedal phalanx was in contact with the top of the inverted *Triceratops* skull (1235.6), another (1235.4) was two meters east of the *Triceratops* skull and a third (1235.6) was two meters farther east from that and a pedal claw (1235.4) another meter east from that. A phalanx of the hallux (1235.3) was two meters north-northeast of that. An as-yet unidentified fragment (1235.3), likely attributable to *Anzu*, was located 12 meters north and four meters west of the *Triceratops* skull. Fig 6B illustrates the skeletal recovery. Although the remains of this taxon were scarce compared to the others, those bones with elongate axes (10) were arrayed in a west-southwest-east-southeast pattern (Fig 10C).

#### Thescelosaurus.

This specimen is unambiguously referred to *Thescelosaurus* based on the presence of frontals wider at the midorbital level than across the posterior end (HRS 15489), which is recognized as an autapomorphy of the genus [23,24]. A future publication will describe the specimen in greater detail. On the Hanson Ranch generally, *Thescelosaurus* is a rare taxon. In 29 years of excavation, only 46 bones and teeth attributable to *Thescelosaurus* have been excavated apart from the specimen described below (http://fossil.swau.edu). This particular specimen was first encountered at Triceratops2 Quarry in 2014 while excavating the *Triceratops* skull (Fig 9, red bones). A femur, a pedal phalanx, and three caudal vertebrae were uncovered three meters east of the *Triceratops* skull and at the same elevation (1235.5). The following season, a large portion of a *Thescelosaurus* skeleton was uncovered in a one square meter area just north of the femur and at approximately the same elevation. Fifteen disarticulated skull pieces were found in close association under the mostly articulated pelvic bones. Forty associated vertebrae (dorsal and caudal) were found directly to the north and 15 ribs (six right, eight left, one indeterminate) were scattered nearby. Pelvic appendages were more completely represented than forelimbs, and proximal elements were better represented than distal elements. The sacrum, right ilium, right and left ischia, pubes, and femora, a left tibia, and fibula, three metatarsals, thirteen pedal phalanges and three pedal unguals represented the hindlimbs and a left scapulocoracoid and a possible radius represented the forelimbs. A total of 118 elements were recovered (Fig 11C). One particularly vexing challenge with this excavation was the variable matrix. Part of the bones occurred in sandy matrix, and these were quite fragile, whereas others were encased in an indurated carbonate cemented sandstone. These bones often required work with an air chisel to disengage them from the matrix. During subsequent seasons, as the site was expanded, other scattered *Thescelosaurus* elements were exposed. In 2016, a third, larger pubis (1235.8) was uncovered five meters east and two meters south of the *Triceratops* skull. It is clear that the tightly clustered remains belong to a single individual, but this third larger pubis indicates that more than one individual is represented in this site. While the bone distribution for the *Thescelosaurus* does not give much directionality, the overall distribution of bones with elongate axes (Fig 10D) is similar to that of other specimens in this quarry.

#### Edmontosaurus.

It was not until the third season of excavation here (2016) that the first bones of *Edmontosaurus* were recovered from the site (Fig 9, yellow bones). This specimen is referred to *Edmontosaurus* because it has a circular distal blade of the pubis (HRS 20951) that is much more expanded ventrally than dorsally [25]. Because *E. annectens* is distinguished from *E. regalis* morphologically by cranial bones [25], the stratigraphic position in the Maastrichtian was used to refer this specimen to *E. annectens*. No cranial bones from this *Edmontosaurus* were recovered. Sixty-five articulated caudal vertebrae (1235.6) were excavated from a region six to eight meters north of the *Triceratops* skull and at the same elevation. Twenty-nine chevrons, four ribs (1235.4, 1235.2, 1235.2, 1235.4), and numerous tendons were also excavated. Two scapulae (both 1234.8), a single humerus (1235.2), radius (1235.5), and coracoid (1234.8) were also recovered. Subsequent expansion of the quarry yielded two each of ilia (1235.2, 1235.5), ischia (1235.2, 1235.5), and pubices (1235.2, 1235.5). A single tibia (1235.0) was also recovered. One pubis was positioned vertically in the sandy sediment, without any obvious evidence for trampling on the bone itself or in the associated bones. The *Edmontosaurus* humerus (1235.1) was recovered 0.5 m east of the *Triceratops* skull. A third *Edmontosaurus* pubis (1235.7) was excavated eight meters east of the *Triceratops* skull at the easternmost boundary of the quarry, so the remains of more than one animal are represented here, just as was the case for the *Thescelosaurus*. However, the distribution of bones with the closely associated pelvic bones and the assemblage of caudal vertebrae nearby support the assertion that the bulk of the *Edmontosaurus* bones represent one specimen (Fig 11D), and that it remained closely associated during transport and deposition. Excavation of the area surrounding these bones was continued for a minimum of two meters laterally to determine whether there could be more of the skeleton present. The general pattern of bone orientation for elongate bones of this specimen (Fig 10E) is northwest to southeast, paralleling the distribution of the bones across the quarry.

#### Testudines.

Several bones attributable to Testudines were also recovered. Many of the bones are isolated pieces of plastron or carapace, but one nearly intact hypoplastron (1235.6) and several turtle limb bones (1235.5, 1235.6) were among the recovered remains. The orientation of these bones echoes other trends seen in this quarry, generally along a northwest-southeast line.

### Triceratops1 Quarry

This site is located 230 meters southeast from Triceratops2. It was recognized by the presence of a protruding brow horn of a ceratopsid cranium (Fig 12). Because it was at the top of a ridge, only limited excavation could be carried out. In addition to the partially complete skull with a single brow horn oriented to the southwest, a premaxilla, jugal, parietal, and dentary (all 1243.2) and several ceratopsid bone fragments were recovered (Fig 11E). The few bones recovered with elongate axes (6) were directed toward the northwest and southeast (Fig 10F). Excavation of the two-meter area surrounding the skull did not yield additional bones, reinforcing the conviction that all remaining bones had been recovered from the site.

**Fig 12:**
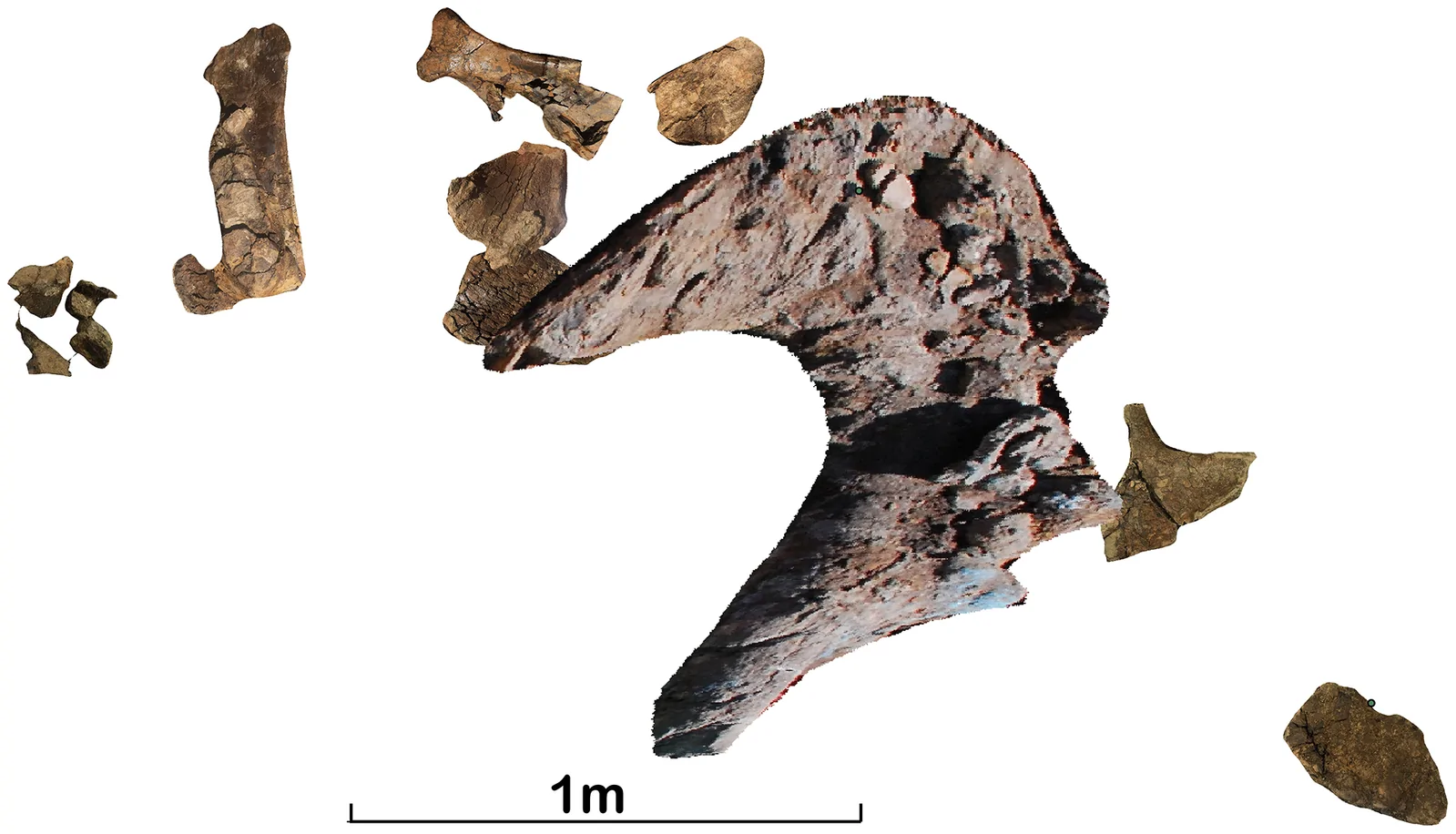
Triceratops1 Quarry showing all bones recovered. North is up.

### Atullah2 Quarry

Another quarry site was opened 90 meters east and 75 meters south from Triceratops2 Quarry. This site (Fig 13) has revealed the partially articulated remains of an *Edmontosaurus* skeleton. Aside from a couple of as-yet unidentified skull fragments, cranial elements were absent. There were two disarticulated (1241.2, 1241.3) and nine associated dorsal vertebrae (1241.1–1241.2) with most of the ribs (9 right, 5 left) in proximity. Nine caudal vertebrae (1241.0–1241.3) and nine chevrons (1240.9–1241.2) were recovered (Fig 11F). From the pectoral girdle, one radius (1241.1) and one metacarpal (1241.1) and from the pelvic girdle, an ilium (1241.3), ischium (1241.1), pubis (1241.2), right and left femora (1241.1), a right metatarsal and a pedal phalanx were recovered. According to the ranch owners, the area around these remains had served as a source of eroding bones which were given away as souvenirs. Presumably much more of the skeleton was originally present. Initially, it seemed plausible that this specimen could represent the body axis of the *Edmontosaurus* in Triceratops2 Quarry, but the presence of redundant pelvic elements countered that possibility. When all bones with a detectable long axis were plotted on a rose diagram (Fig 10G), the directionality was northwest and southeast.

**Fig 13:**
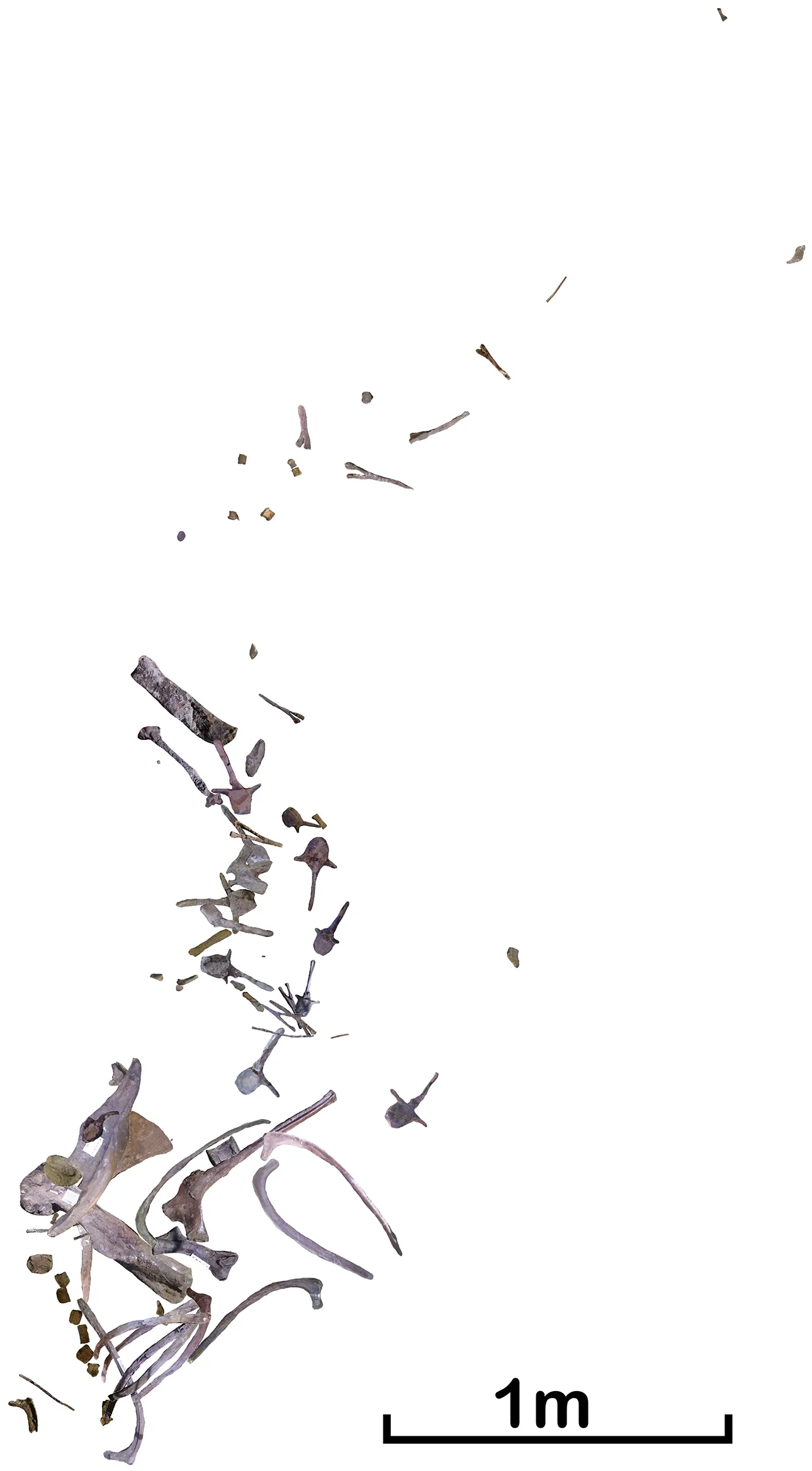
Atullah2 Quarry showing all bones recovered. North is up.

### JW Quarry

This fourth quarry is just over two kilometers northwest of Triceratops2. JW Quarry (Fig 14) adds significant taphonomic data relevant to the process of disarticulation and transport of the remains of individual specimens. In this site, the disarticulated remains of a single *Triceratops* were uncovered. The inverted skull (1200.0), including the braincase and brow horns in the upper left of Fig 14, was surrounded by a mass of bones presumed to be from the same animal in an area of about 16 square meters. The horns were oriented in a northeasterly direction. Additional cranial elements recovered include right and left jugals (1200.3, 1200.5), a predentary (1200.6), a right quadrate (1200.3), a quadratojugal (1200.8), a lachrymal, right and left squamosals, and a toothless dentary. The spinal column was represented by the syncervical (1200.5), eight dorsal vertebrae (1200.4–1200.8), ten left and eight right ribs (1200.1–1200.9), and 22 partial ribs. As was the case at the other *Triceratops* quarries, there were few limb and tail bones, but other parts of the skeleton were well represented. These included right and left scapulae and a right coracoid (all 1200.5) from the pectoral girdle and the sacrum (1200.4), an ilium (1200.8), and the right pubis (1200.7) from the pelvic girdle. The left femur (1200.2), an astragalus, one possible phalanx (both 1200.4) were also recovered. Continued excavation at this site suggested most bones have been recovered (Fig 11G). The long axes of elongate elements (23) trends northeast-southwest (Fig 10H).

**Fig 14:**
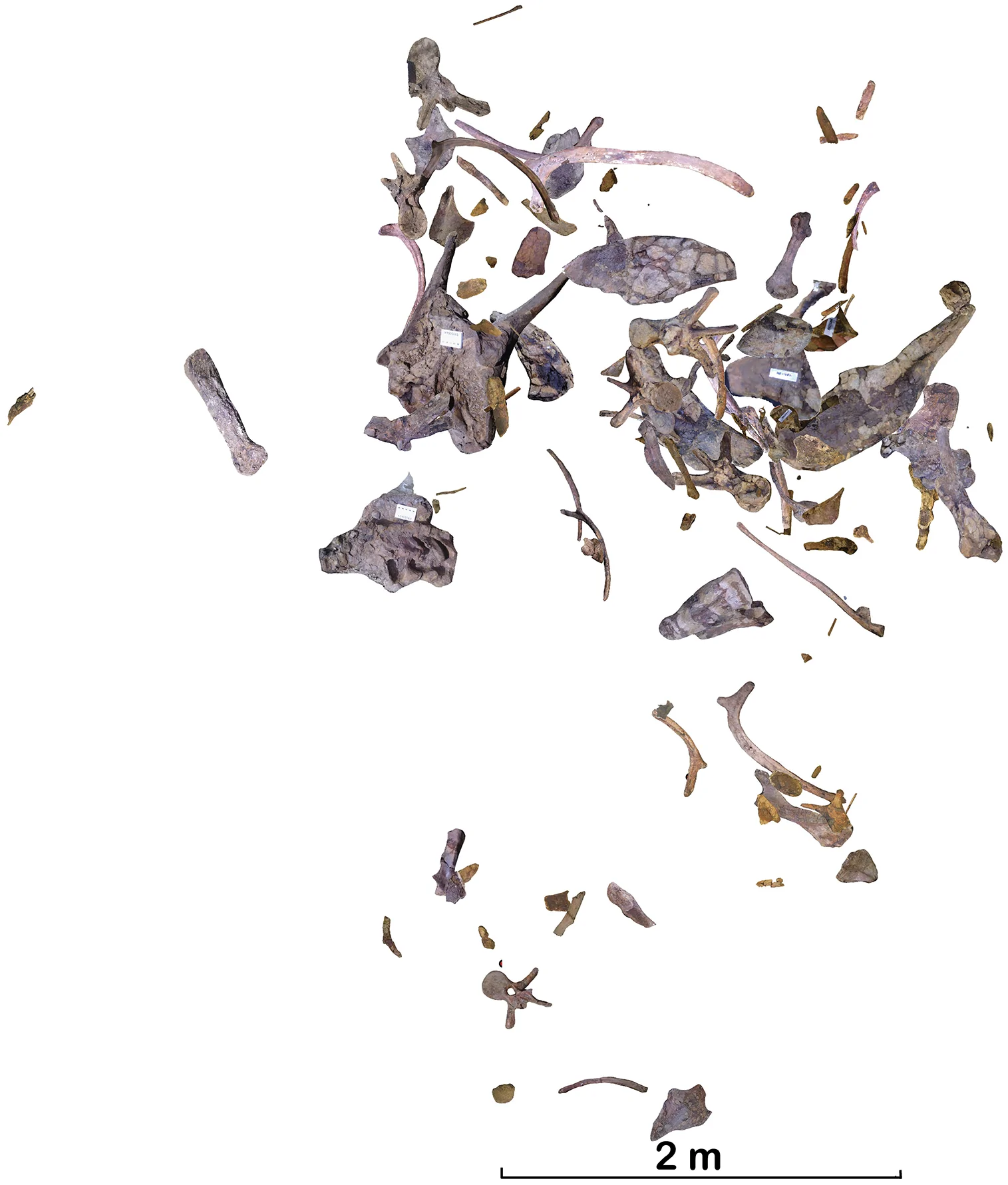
JW Quarry showing all bones recovered. North is up.

### Triceratops4 Quarry

This quarry, discovered in 2021 revealed additional taphonomic data from disarticulated but associated *Triceratops* remains (Fig 15). Excavation was halted in 2025 because the site is up against a high bank, and further excavation was deemed impractical. Thus far the only cranial elements recovered were an occipital condyle (1238.3), a squamosal and a single epoccipital. The vertebral column is represented by three dorsal vertebrae (1238.4), one possible cervical vertebra (1238.3), two centra, 23 ribs (three left, four right and 16 partials, all 1238.3), and one chevron (1238.2). The pectoral girdle includes a right scapula (1238.5) and a right metacarpal (1238.3). The pelvic girdle was represented by a left pubis (1238.4), paired right and left ischia (1238.3), a left ilium (1238.2), right and left femora (1238.1, 1238.3), a right tibia and fibula (1238.4, 1238.3), and a metatarsal (1238.4), all presumably from the same *Triceratops* (Fig 11H). Thus far, the large majority of the elongate bones (23) appear to be oriented in east-west and northeast-southwest directions (Fig 10I).

**Fig 15:**
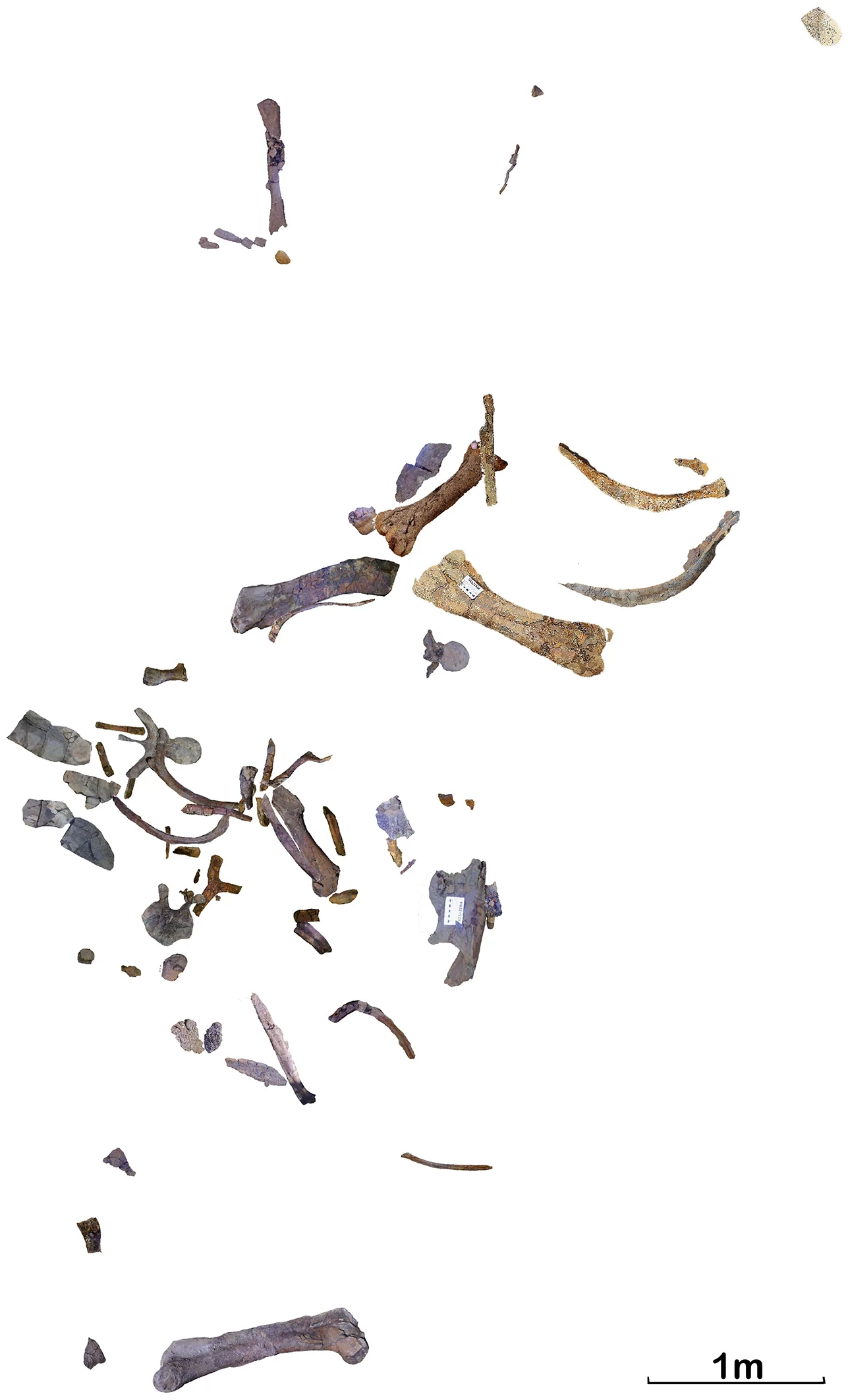
Triceratops4 Quarry showing all bones recovered. North is up.

### Rabbit Ears Quarry

This quarry, also encountered in 2021, was explored at the surface and has not yet been excavated. The majority of dinosaur bones identified at this site were field-identified as either frill or bone fragments consistent with *Triceratops* (Fig 16). Bone elevations ranged from 1208.5 to 1208.7 meters. The elongate bones (6) had an orientation of northwest to southeast (Fig 10J).

**Fig 16:**
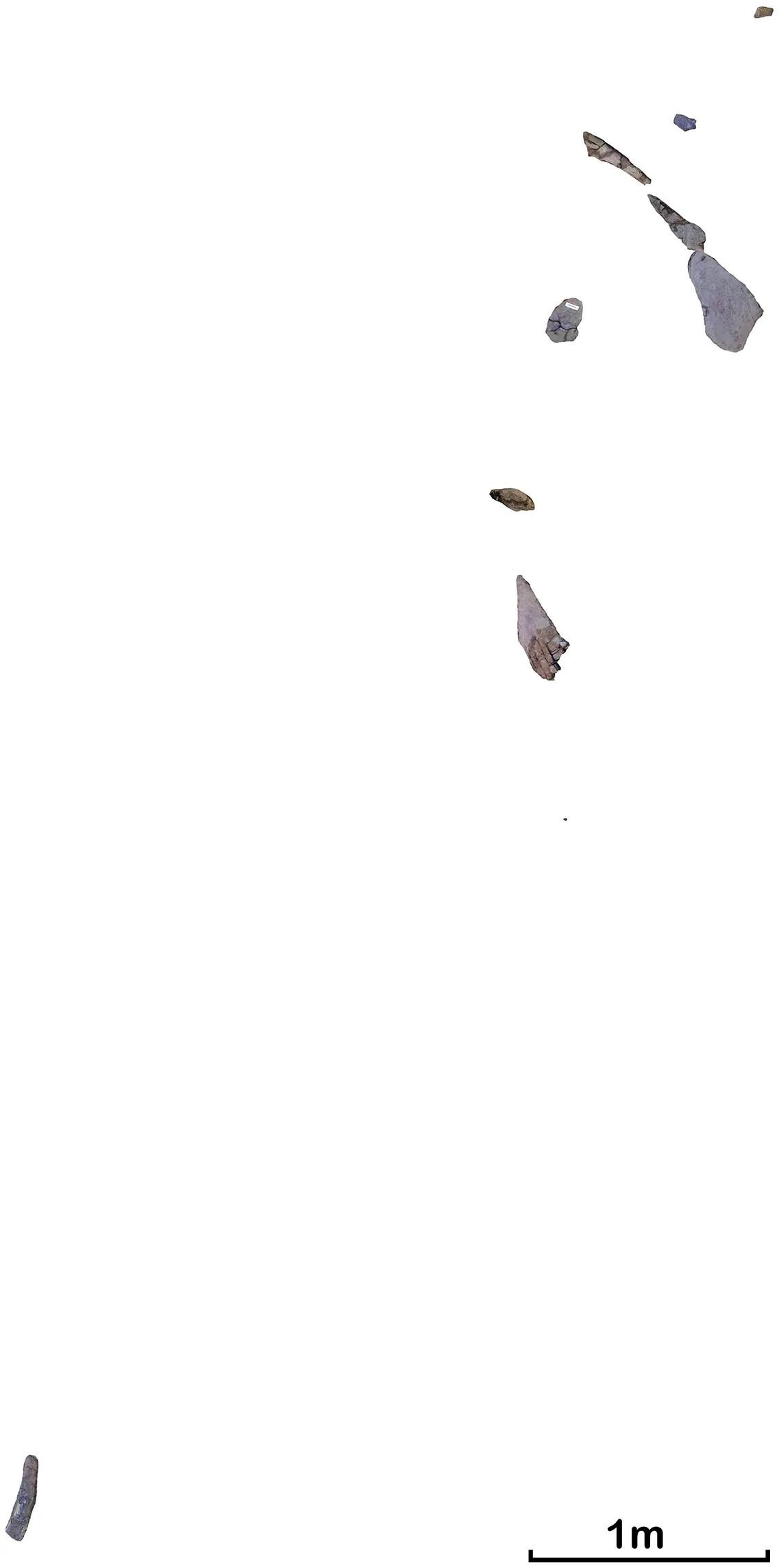
Rabbit Ears Quarry showing all bones observed. North is up.

### Triceratops5 Quarry

This site was discovered in 2021 during reconnaissance (Fig 17). The initial indication of bone was a partially exposed *Triceratops* squamosal. Further exploration of the area in June 2022 revealed several more pieces of frill and two occipital condyles. Approximately 25 meters to the SW from the initial discovery site, another site was located which quickly revealed a scapula with associated coracoid, several dorsal vertebrae, and assorted pieces of frill and tendons which were removed in 2022. During the subsequent three seasons, excavation continued at this site, revealing over 200 bones of *Triceratops* including many fragments of frill, an intact braincase with occipital condyle (1223.8), left and right squamosals (1223.6, 1223.8), a right lachrymal (1223.6), a maxilla (1223.9), right and left dentaries (1223.8, 1223.9) and a horn core (1223.9). The axial skeleton included the syncervical (1223.9), a total of thirteen dorsal vertebrae (1223.7–1223.9), a hyoid (1223.7), a chevron (1223.6), twenty-six mostly intact ribs (10 right and 16 left, all 1223.7–1223.9) and additional rib fragments. The pectoral girdle included the left scapulocoracoid (1224.6) and right coracoid (1224.8), and a humerus (1223.3). The pelvic girdle included a sacrum (1223.7), right and left pubes (1223.7), a right ischium (1223.7), and two astragali (1223.6, 1223.8), all from *Triceratops* (Fig 11I). Although this site has been mostly excavated, there are nearby locations with *Triceratops* bones that are yet to be explored. The ribs lie mostly parallel to each other and to the femur in a northwest to southeast orientation. The directional disposition of the bones excavated so far is shown in Fig 10K.

**Fig 17:**
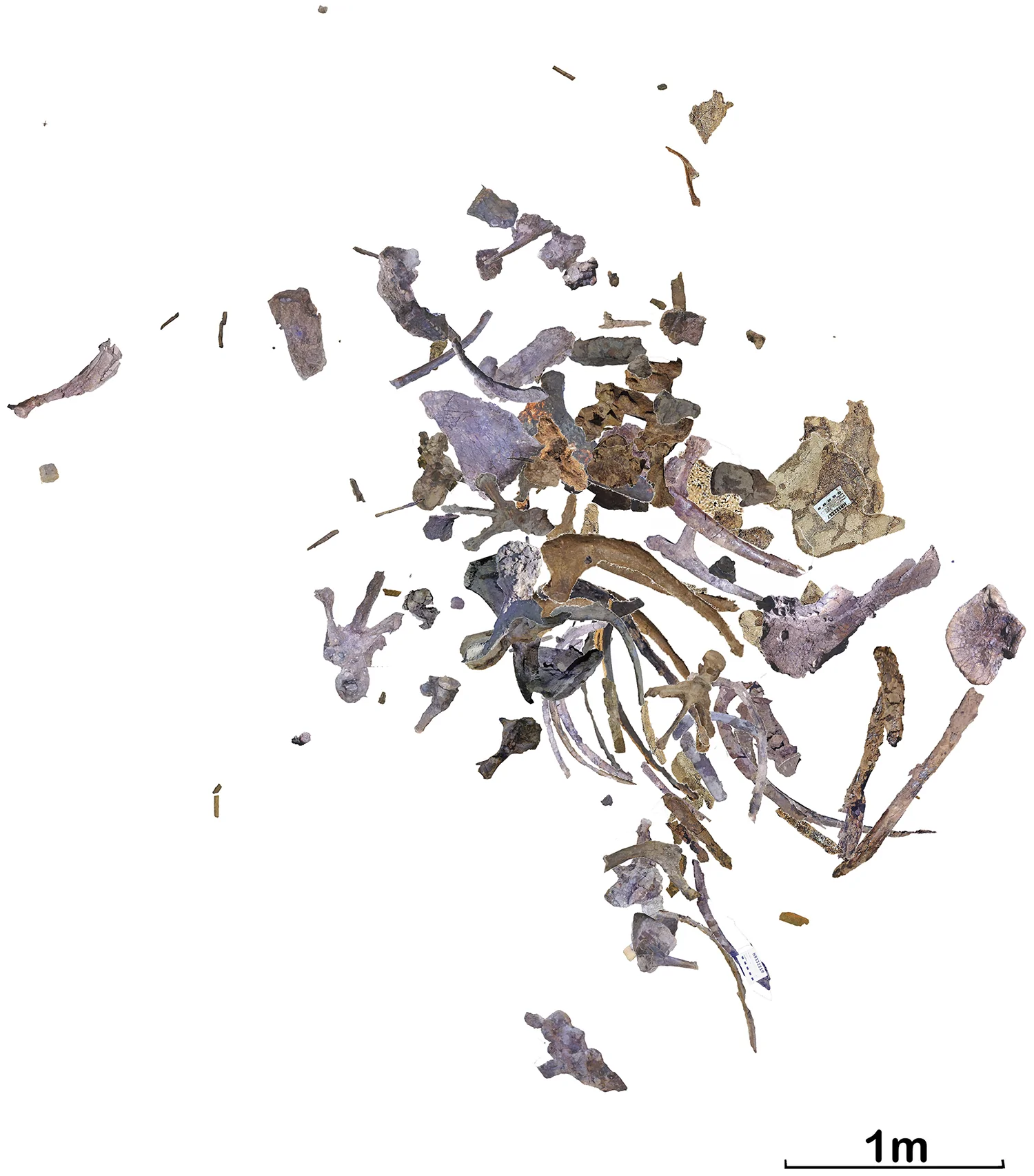
Triceratops5 Quarry showing all bones recovered. North is up.

## Discussion

### Patterns of skeletal association

The quarries described in this study differ markedly from the extensive bonebed previously documented on the Hanson Ranch. Whereas the main bonebed is dominated by disarticulated and dissociated skeletal elements [5], the remains in the seven quarries that are the subject of this report preserve varying degrees of skeletal association and in some cases articulation. This difference is significant because it reveals a different taphonomic history from death to burial for these bones. The preservation of associated skeletal remains indicates that complete disarticulation had not occurred prior to final deposition. In these quarries the bones of the animals remained associated in spite of evidence of transport suggesting the presence of soft tissues with significant integrity to permit their movement and burial as a unit. This is in sharp contrast with the situation in the main bonebed a kilometer east of the quarries described above, where complete dissociation and disarticulation is the rule. It also contrasts with the fragmented and extensively dispersed remains characteristic of many fluvial lag deposits [26]. The repeated occurrence of associated/articulated skeletons across multiple localities further suggests that the observed pattern of preservation is not a taphonomic anomaly. Rather it likely defines a set of recurring depositional conditions capable of preserving skeletal associations over a broad area.

### Carcass transport and differential preservation

One of the most evident features of the assemblage is the substantial variation in preservation among taxa and individuals. Triceratops2 Quarry contains a tightly clustered and relatively complete *Thescelosaurus* skeleton alongside considerably more dispersed remains of *Anzu*, partially associated remains of *Triceratops* and a partially articulated and associated *Edmontosaurus*. This variability suggests that the carcasses entered the depositional system in different states of decay. Differences in body size, buoyancy, transport distances and stages of decomposition all may have been operative in determining the state of preservation of the remains. Experimental studies of vertebrate decomposition demonstrate that carcasses may remain intact for different amounts of time depending on the environmental conditions and biological factors [27]. Thus, the variation in preservation seen in Triceratops2 Quarry does not in itself require disparate depositional environments. In fact, it seems clear that the various carcasses entered a common depositional setting at different stages of decay. Although this wide variation in states is informative, it almost necessitates a somewhat different history for each specimen.

Decomposition due to varying time since death could also explain different distribution patterns seen in the quarry with bones in the most intact carcass (*Thescelosaurus*) remaining together and the more decomposed carcasses spreading out due to wave action and longshore drift. A shoreline irregularity could explain the rich accumulation of carcasses in one place, and result in the wider range of bone orientation for this particular quarry. The overall pattern of alignment of bones in the quarry suggests transport of some sort is a part of the explanation.

Several authors have proposed bloating and floating for dinosaurs found in marine deposits [28–30] and for the frequent discoveries of upside-down ankylosaurs [31,32]. Bloating and floating has been observed in many extant marine vertebrates, with the mandibles and head detaching from the rest of the carcass [33]. Schäfer [33] found from experiments with seal decomposition that the lower jaws detached after 36 days of floating at the surface, and by 41 days “the carcass is still afloat but it now resembles a loose sack” (p. 33). After 48 days, Schäfer [33] noted that the body was open at both ends, but that the vertebral column, rib cage, shoulder blades, pelvis, and thighs were still in the floating sack of skin. Brand et al. [34] with lizards, and Syme and Salisbury [35] with crocodiles, have shown that modern reptiles, allowed to decay in water, are also surrounded by a tough sack of skin that can retain the bones of the creature for extended periods after death. Although dinosaurs like *Triceratops* and *Edmontosaurus* are orders of magnitude larger than lizards, the similar decay in floating seals and extant reptiles suggests that encapsulation and transport of bones in a sack of skin is a realistic possibility to explain some of the taphonomy encountered. Crocodiles [35], lizards [34], and seals [33] initially sank to the bottom after death, then rose and floated at the surface for a protracted period before coming to final rest on the bottom. During the interim, the floating sac of dermis/epidermis tended to keep the bones together as disarticulation ensued. Since both Brand et al. [34] and Syme and Salisbury [35] documented the loss of bones from the dermal/epidermal sac during transport and/or flotation, a similar package of dinosaur remains could be delivered to the site of ultimate burial in a variety of different states, as argued for varying taphonomic modes of dinosaur fossils in marine deposits in Eastern North America [28]. The common occurrence of dinosaur specimens lacking skulls [36] matches well with taphonomic observations of floating carcasses losing heads before elements of the torso [33,34]. The loss of the skull would leave a tear in the dermal sack. This tear may have opened the door to rapid loss of cervical vertebrae in floating dinosaur carcasses, which would explain the absence of most cervical vertebrae from all seven sites. The absence of caudal vertebrae from all sites except for the mostly intact *Edmontosaurus* tail found in Triceratops2 and a few proximal caudals from the *Edmontosaurus* at Atullah2 Quarry, can be explained by the early loss of tails prior to stranding and burial.

### Loss of teeth from jaws

In four quarries a total of nine dentaries and three maxillae were identified. Eight of these were from *Triceratops*. The remaining dentary was from *Thescelosaurus.* All the dentaries and maxillae were within meters of the skulls, yet none of them contained teeth. This would suggest that the loss of teeth from these skulls may have occurred during the decomposition of the skeleton prior to transport, rather than during any transport that may have accompanied burial, since the teeth were not generally found with the skeletons [37]. This is what Brand et al. [34] found for modern mammal teeth and Syme and Salisbury found for crocodiles [33]. Teeth were often lost from jaws early, especially when the bones were in water. Similar tooth loss is commonly observed in the jaws of ornithopod [38] and theropod [39] dinosaurs, where the rapid decay of periodontal ligaments is linked to the rapid postmortem tooth loss. The rarity of cracks on isolated teeth that were found is consistent with disarticulation occurring in water [34].

### Evaluation of bone orientation data

Preferred orientation of elongate skeletal elements is evident in most of the studied quarries. Rayleigh analyses indicate that elongate bones commonly exhibit statistically significant alignment, and the dominant orientations are broadly similar among localities. Such alignment reflects the influence of directional processes during transport and deposition. While directional orientation of the bones alone does not uniquely identify the depositional environment responsible for that alignment, the northwest-southeast trend is consistent with a single depositional process operative across the extent of the quarries.

Long-bone orientation constitutes axial rather than directional data, and the measured alignments do not independently establish the sense of paleo-flow direction. But the consistent trend throughout the quarries implies the directional data have a sense. In this case, independently derived data obtained from local paleocurrent indicators align with the long-axis data from the bones and give a preferred sense for the bone transport toward the southeast (Fig 8, [40,41]).

The consistency of directional sense among multiple quarries supports the hypothesis that the remains were influenced by a recurring hydrodynamic regime operating across a substantial portion of the study area. This suggests that the bones/carcasses were in water prior to deposition and that longshore currents or southeasterly flowing water currents were involved in the deposition. Of further interest is the observation that all seven quarries, the only quarries opened on this Ranch that have significant associated remains, lie on or near a line that also trends toward the southeast at 140 degrees (Fig 8). This alignment is consistent with a shoreline accumulation of floating carcasses, since it roughly parallels the expected shoreline of the WIS (Fig 3). This linear feature is consistent with a transient Upper Cretaceous shoreline along the WIS. Strandlines and strandline deposits have long been recognized as important elements in environmental interpretations [42]. Sometimes the term “strandline” is used generically to represent what is believed to be a high-stand (or low-stand) deposit or surface during a transgressive (or regressive) event [43]. Sometimes specific kinds of deposits such as pebble beds have been identified as relict strandline deposits [44,45]. Specific physical characteristics of strandlines themselves have also been recognized. For example, Binns [46] identifies what he assumes to be physical strandlines by the presence of pumice drift accumulations in Quaternary sediments in Great Britain. Dinosaur trackways have also been used to define strandlines [47,48].

Longshore drift can explain the similarity of the long bone orientation as the remains were liberated from the rotting stranded carcass and moved laterally along the shoreline [49–51]. Bones aligned in nodules contributed to identifying a strandline deposit in Argentina [52]. Rogers and Kidwell [50], Varricchio [53], and Tasch and Zimmerman [54] all suggest aligned bone concentrations are consistent with a strandline origin. The Tendaguru deposits of Tanzania contain the associated, but largely disarticulated bones of sauropods, in much the same state as the bones described in this report [55–57]. These deposits are described as strandline deposits. The bodies of the dinosaurs were transported from the place of death to the site of burial by water. Weigelt [58] identifies massed bone deposits as strandline accumulations. In Weigelt’s case and others, the alignment is the definitive factor warranting a strandline designation.

### Stratigraphic relationships and associated uncertainty

The skeletal remains at these seven sites would have been at similar elevations if they comprised a single strandline deposit. The recovered bones range in elevation from 1240 meters in the east to 1200 meters in the west. However, the quarries lie along the eastern limb of a north-south structural basin. Since the present dip of this arm of the basin is 3–5 degrees toward the west, sites that were the farthest west (Rabbit Ears and JW) would today rest at a lower elevation than those farther east, just as is seen. Thus, the elevations that occur are consistent with a series of strandings closely related in time. All of the bonebed-containing facies appear to be within a single coarsening-upward cycle (C1), allowing the possibility that these remains record related stranding events.

Three of the quarries considered in this paper (Rabbit Ears, Triceratops4, and Triceratops5) were originally located by testing this strandline hypothesis and walking out the ancient shoreline in search of additional sites. It will be interesting to see what further discoveries can be made by continuing to search along this trajectory.

### Evaluation of alternative depositional models

Several depositional models may potentially explain the observed distribution of associated dinosaur remains. Fluvial channel-lag deposits are capable of concentrating vertebrate remains and producing preferred orientation of elongate skeletal elements through hydraulic processes [59–61]. Such deposits commonly exhibit hydraulic sorting, selective preservation of resistant skeletal elements, and extensive disarticulation resulting from transport and reworking [60,61]. Consequently, the repeated occurrence of associated skeletons at multiple localities is more consistent with limited transport and relatively rapid burial than with prolonged accumulation within a reworked channel-lag deposit [61,62].

Point-bar deposits can generate preferred orientation of elongate skeletal elements through hydraulic alignment and sorting [26,59]. However, because the taphonomic characteristics of such deposits are strongly influenced by local hydrologic and depositional conditions, the repeated occurrence of associated skeletons across multiple localities suggests the recurrent operation of similar preservational processes rather than stochastic hydraulic concentration alone [62,63]. Floodplain pond or oxbow-lake settings may preserve associated skeletons but generally do not predict the consistent alignment of multiple quarries along a common regional trend, nor do they readily explain recurring preferred orientations observed across the assemblage.

Deltaic environments remain plausible alternatives. Such settings are characterized by complex interactions among fluvial, tidal, and wave-driven processes [64,65] that can transport, rework, and concentrate vertebrate remains while preserving a broad spectrum of taphonomic states ranging from isolated elements to associated or partially articulated skeletons [66,67]. Each of these models accounts for some aspects of the observed data. However, none individually explains the combined occurrence of associated skeletons, recurring orientation patterns, stratigraphic similarities, and regional spatial alignment as effectively as a shoreline-influenced depositional setting.

### Evidence for shoreline influence

Several independent observations suggest proximity to a shoreline environment during deposition. These include the regional paleogeographic position of the study area during regression of the WIS, the occurrence of glauconitic material, especially glauconitic cement [18,19], within some sandstone units, the presence of laterally persistent sedimentary cycles, and the organization of quarry localities along a northwest–southeast transect. None of these observations independently demonstrates shoreline deposition. Nevertheless, when considered collectively, these observations are consistent with deposition in a setting influenced by shoreline processes. The proposed shoreline interpretation does not require all carcasses to have been deposited simultaneously or during a single catastrophic event since repeated accretion of carcasses within a broadly similar depositional setting during shoreline migration could generate the observed distribution.

### Preferred depositional interpretation

The combined taphonomic, stratigraphic, sedimentological, and paleogeographic evidence is most consistent with deposition in a shoreline-influenced environment associated with the final regression of the Western Interior Seaway. Such an environment would provide a setting for concentrating carcasses, maintaining partial skeletal association, and generating recurring directional alignment of elongate skeletal elements.

The strandline interpretation proposed here should be regarded as the preferred working hypothesis rather than a demonstrated conclusion. Future investigations incorporating higher-resolution sedimentological analyses, quantitative mineralogical characterization, and additional stratigraphic control will provide important tests of this hypothesis and further refine interpretations of vertebrate accumulation within the Lance Formation.

## Conclusions

Along a 3.25 km northwest-southeast line, seven apparently related taphonomic quarry sites in the Lance Formation of NE Wyoming have each produced articulated and associated bones of one or more individuals. Predictive exploration along this presumed strandline led to the discovery of three of the seven quarries. They predominantly contain remains of *Triceratops*, but also include *Edmontosaurus, Thescelosaurus, Anzu*, and non-dinosaurian remains including turtles and crocodilians. These remains are best explained as partially disarticulated carcasses that retained some association during aquatic transport and shoreline stranding. The loss of teeth from the jaws is consistent with modern mammal and crocodile disarticulation in water [34,35] and expected from the rapid decay of the periodontal ligaments during flotation of the carcasses. The general orientation of long bones from each of these sites is from the NW to SE (approx. 140 degrees), matching the orientation of the quarries and the proposed strandline. Longshore drift provides a reasonable explanation for the similarity of long bone orientation. This, along with local and regional paleocurrent data suggest stranding of floating carcasses on a shoreline of the easterly regressing Western Interior Seaway toward the end of the Cretaceous.
