# Supplementary material for: Evidence for an upper cretaceous strandline deposit in the Lance Formation, Wyoming, USA
Source: PLoS One. 2026 Nov 12;21(11):e0362687. doi: 10.1371/journal.pone.0362687 (PMC13759098; doi:10.1371/journal.pone.0362687)

PLOS One

Identification of an Upper Cretaceous strandline in the Lance Formation, Wyoming, USA

--Manuscript Draft--

**Manuscript Number:**	PONE-D-26-22387
**Article Type:**	Research Article
**Full Title:**	Identification of an Upper Cretaceous strandline in the Lance Formation, Wyoming, USA
**Short Title:**	Identification of a strandline in the Lance Formation
**Corresponding Author:**	Keith Snyder, Ph.D.<br>Southern Adventist University<br>Collegedale, Tennessee UNITED STATES OF AMERICA
**Keywords:**	Lance Formation; Upper Cretaceous; Strandline deposit; Dinosaur taphonomy; Western Interior Seaway
**Abstract:**	Abstract<br>A series of apparently related quarries in the Maastrichtian (uppermost Cretaceous) Lance Formation of Wyoming provided us the opportunity to evaluate the taphonomic mode for a suite of vertebrate fossils. Extensive work on the Hanson Ranch over the past three decades has exposed tens of thousands of fossils from dinosaur-bearing bonebeds. All of these bonebeds consisted of disarticulated and disassociated remains. However, seven recently discovered quarries have yielded the remains of one or more dinosaur taxa (e.g., Triceratops, Edmontosaurus, Thescelosaurus, Anzu) in a state of association that was hitherto unknown on the ranch. These seven quarries were found along a line heading approximately 145 degrees, a heading reflected in the long axes of the elongate bones in the quarries. Because the bones were still associated after transport and burial, we can know that the carcasses were still associated during the disarticulation process. We conclude that the floating, encapsulated carcasses of these animals were stranded along a southeasterly trending shoreline of the Western Interior Seaway.
**Order of Authors:**	Keith Snyder, Ph.D.
	Nelson Llempen
	Caleb Darling
	Alessandro Franchini
	Matthew McLain
	Arthur Chadwick
**Additional Information:**	
**Question**	**Response**
**Financial Disclosure**<br><br>Enter a financial disclosure statement that describes the sources of funding for the work included in this submission. Review the submission guidelines for detailed requirements. View published research articles from *PLOS ONE* for specific examples.<br><br>This statement is required for submission	The author(s) received no specific funding for this work.

Powered by Editorial Manager® and ProduXion Manager® from Aries Systems Corporation

and **will appear in the published article** if the submission is accepted. Please make sure it is accurate.<br><br>**Funded studies**<br>Enter a statement with the following details:<br>• Initials of the authors who received each award<br>• Grant numbers awarded to each author<br>• The full name of each funder<br>• URL of each funder website<br>• Did the sponsors or funders play any role in the study design, data collection and analysis, decision to publish, or preparation of the manuscript?<br><br>Did you receive funding for this work?	
**Competing Interests**<br><br>Use the instructions below to enter a competing interest statement for this submission. On behalf of all authors, disclose any competing interests that could be perceived to bias this work—acknowledging all financial support and any other relevant financial or non-financial competing interests.<br><br>This statement is required for submission and **will appear in the published article** if the submission is accepted. Please make sure it is accurate and that any funding sources listed in your Funding Information later in the submission form are also declared in your Financial Disclosure statement.<br><br>View published research articles from *PLOS ONE* for specific examples.	The authors have declared that no competing interests exist.

**NO authors have competing interests**<br><br>Enter: *The authors have declared that no competing interests exist.*<br><br>**Authors with competing interests**<br><br>Enter competing interest details beginning with this statement:<br><br>*I have read the journal's policy and the authors of this manuscript have the following competing interests: [insert competing interests here]*<br><br>* typeset	
**Ethics Statement**<br><br>Enter an ethics statement for this submission. This statement is required if the study involved:<br><br>• Human participants<br>• Human specimens or tissue<br>• Vertebrate animals or cephalopods<br>• Vertebrate embryos or tissues<br>• Field research<br><br>Write "N/A" if the submission does not require an ethics statement.<br><br>General guidance is provided below. Consult the submission guidelines for detailed instructions. **Make sure that all information entered here is included in the Methods section of the manuscript.**	N/A

**Format for specific study types**<br><br>**Human Subject Research (involving human participants and/or tissue)**<br>• Give the name of the institutional review board or ethics committee that approved the study<br>• Include the approval number and/or a statement indicating approval of this research<br>• Indicate the form of consent obtained (written/oral) or the reason that consent was not obtained (e.g. the data were analyzed anonymously)<br><br>**Animal Research (involving vertebrate animals, embryos or tissues)**<br>• Provide the name of the Institutional Animal Care and Use Committee (IACUC) or other relevant ethics board that reviewed the study protocol, and indicate whether they approved this research or granted a formal waiver of ethical approval<br>• Include an approval number if one was obtained<br>• If the study involved *non-human primates*, add *additional details* about animal welfare and steps taken to ameliorate suffering<br>• If anesthesia, euthanasia, or any kind of animal sacrifice is part of the study, include briefly which substances and/or methods were applied<br><br>**Field Research**<br><br>Include the following details if this study involves the collection of plant, animal, or other materials from a natural setting:<br>• Field permit number<br>• Name of the institution or relevant body that granted permission	
Data Availability<br><br>Before publication, Authors are required to make fully available and without restriction all data underlying their findings. Sharing the data underlying your findings increases the visibility, credibility, and impact of your work, while supporting transparency and future scientific discovery. Please see our PLOS Data Policy page for detailed information on this policy.</p><br>A Data Availability	The minimal data set is available at https://fossil.swau.edu/

Statement, detailing where the data can be accessed, is required at first submission. Insert your Data Availability Statement in the box below.</p><br><br>Please see the data reporting section of our submission guidelines for instructions on what you need to include in your Data Availability Statement. Examples of acceptable Data Availability Statements are included here for reference:</p><br> <br> - The minimal data set [and accompanying code, where generated] is available at [repository name] via [DOI hyperlink] <br> - No data was generated by this study. The following existing data sources were used: [dataset name] from [dataset location] available via [web address or DOI hyperlink]. <br> - For Study Protocols: No datasets were generated or analysed during the current study. All relevant data from this study will be made available upon study completion. <br> <br><br><i>PLOS allows rare exemptions to address legal and ethical concerns. If you have legal or ethical restrictions, please use the box below to detail these in full sentences for the Journal team to consider.</i></p><br><br>This statement will be typeset if the manuscript is accepted for publication.</p>	

Powered by Editorial Manager® and ProduXion Manager® from Aries Systems Corporation

Manuscript - Strandline
Click here to access/download;Manuscript;STRANDLINE - 5-5-26.docx

Identification of an Upper Cretaceous strandline in the Lance Formation, Wyoming, USA

Identification of a strandline in the Lance Formation

Keith Snyder[1*¶], Nelson Llempen[2&], Caleb Darling[2&], Alessandro Franchini[3&], Matthew McLain[4&], and Arthur Chadwick[3¶]

[1]Department of Biology and Allied Health, Southern Adventist University, Collegedale, TN, United States of America

[2]Department of Earth and Biological Sciences, Loma Linda University, Loma Linda, CA, United States of America

[3]Dinosaur Science Museum and Research Center, Southwestern Adventist University, Keene, TX, United States of America

[4]Department of Biological and Physical Sciences, The Master's University, Santa Clarita, CA, United States of America

*Corresponding author

E-mail: kasnyder@southern.edu (KS)

Abstract

A series of apparently related quarries in the Maastrichtian (uppermost Cretaceous) Lance Formation of Wyoming provided us the opportunity to evaluate the taphonomic mode for a suite of vertebrate fossils. Extensive work on the Hanson Ranch over the past three decades has exposed tens of thousands of fossils from dinosaur-bearing bonebeds. All of these bonebeds consisted of disarticulated and disassociated remains. However, seven recently discovered quarries have yielded the remains of one or more dinosaur taxa (e.g., *Triceratops, Edmontosaurus, Thescelosaurus*, *Anzu*) in a state of association that was hitherto unknown on the ranch. These seven quarries were found along a line heading approximately 145 degrees, a heading reflected in the long axes of the elongate bones in the quarries. Because the bones were still associated after transport and burial, we can know that the carcasses were still associated during the disarticulation process. We conclude that the floating, encapsulated carcasses of these animals were stranded along a southeasterly trending shoreline of the Western Interior Seaway.

Keywords:

Lance Formation

Upper Cretaceous

Strandline deposit

Dinosaur taphonomy

Western Interior Seaway

Introduction

The Upper Cretaceous (Maastrichtian) Lance Formation of Wyoming has been a rich source of dinosaur fossils since they were first identified there in the late 19th century [1]. Originally designated as the "*Ceratops* beds" [2,3], or the "*Triceratops* beds" [4], the Lance Formation has long been recognized as a source of *Triceratops* fossils. The fossils generally occurred as isolated specimens in various states of completeness. However, our 29 years of research on the Hanson Ranch in Niobrara County in southeastern Wyoming has been focused on ten quarries in a major hadrosaur bonebed in the Lance (Fig 1; [5]) where disarticulated and dissociated bones predominate, and where we rarely encountered *Triceratops* bones. During the process of exploration, 14 additional quarries have been excavated elsewhere on the Ranch. While these quarries have provided the opportunity to examine the broader context of the taphonomic milieu represented in these Lance beds and have included more remains from ceratopsids (see, for example, [6]), they have not revealed the kinds of associated or articulated fossils that are found elsewhere in the Lance Formation [7].

Fig 1. Study site locality in Niobrara County, Wyoming. Site indicated by star. Study sites are protected but exact location information will be shared with qualified researchers upon request.

The initial impetus to open a new quarry (Triceratops2 Quarry) in 2014 was the discovery of an inverted, but quite complete *Triceratops* skull at the surface about 1.6 km south of the main quarry area. During excavation of the skull, the remains of a mostly complete, partially articulated *Thescelosaurus* were encountered. Further excavation revealed scattered remains of an *Anzu*, and the partially articulated remains of an *Edmontosaurus*, all within an area of approximately 225 square meters [8]. A second site (Triceratops1 Quarry), containing a second ceratopsid skull and a few associated bones was excavated at the same time.

As our work expanded, an additional nearby site was encountered with the partially articulated remains of a single *Edmontosaurus* (Atullah2 Quarry). A fourth site (JW Quarry) was added as a result of excavation begun in 2020, revealing the associated remains of a single *Triceratops*. While examining an aerial view of these sites, we noticed that the three earlier quarries lined up and pointed to this fourth site 2.5 km away. We immediately began to survey along the northwesterly line that connected the quarries. Most of the surface was eroded below the level of the productive sites, but a fifth site (Rabbit Ears Quarry) was discovered on an elevated rise. It has been explored at the surface where several large pieces of *Triceratops* frill were identified but ~~has~~ not yet been excavated. No other sites were exposed along this transect farther to the northwest beyond JW Quarry (Fig 2). However, by following the line to the southeast, we discovered two additional sites (Triceratops4 and Triceratops5), and we have begun excavation at those sites. The unique levels of associated but disarticulated remains represented in all of these quarries provided data that could potentially forward our understanding of the taphonomic history of these Lancian bonebeds. Therefore, we undertook a careful study of the relationship of the remains with a goal of understanding both the processes of disarticulation and association represented in these quarries and the curious distribution of these sites along a southeasterly trending line. These quarries appear to define a portion of the Western Interior Seaway shoreline.

Fig 2. All seven quarries align on a 140-degree azimuth. A) Quarries placed on a Google Earth image. **B**) Quarries with background removed for visibility. North is up.

Geology

The Upper Cretaceous (Maastrichtian) Lance Formation strata being considered in this report consist of unconsolidated to poorly consolidated mudstones and claystones with

interbedded poorly indurated fine-grained white quartz sandstone and well indurated, carbonate-cemented sandstone. As with most Lance outcrops, stratigraphy is poorly defined and until recently has not been successfully used to compare these outcrops stratigraphically with bone-containing beds elsewhere in the area. Historically, the consensus was that the Lance represents sediments from an ancient fluvial system that migrated across this area of Wyoming and South Dakota [9]. However, this view does not appear to hold for the whole of the Lance Formation, which, in eastern Wyoming at this time sits along the shoreline of the Western Interior Seaway (WIS), and we have previously suggested that at least part of the Lance in this area was deposited offshore [5,10]. Cobban et al. [11] identified the lower Maastrichtian shoreline as being to the west of our locality. Although we do not know the precise temporal pattern of shoreline migration in northeastern Wyoming as preserved in upper Maastrichtian deposits, we know that the WIS was receding to the east and the shoreline would have migrated across this location during deposition of the Lance Formation (Fig 3, [12,13]). If this view is true, then somewhere west of the main quarry area, which is deposited offshore [5], we can anticipate the existence of a contemporary strandline deposit.

Fig 3. Paleogeographic map of the Upper Maastrichtian western shoreline of the WIS. Adapted from Roberts and Kirschbaum [12] and Lillegraven and Ostresh [13] illustrating the position of the quarries (star) described in this paper.

Materials and methods

The quarries that are the subject of this report are a kilometer west of the main Hanson Ranch bonebed [5]. Excavation of the sites was carried out using centimeter level resolution RTK GPS to map the positions of all specimens recovered [14]. Each specimen was carefully exposed, assigned a field number, described, and illustrated in a logbook. The specimen was

then photographed, and GPS coordinates were taken from the upper surface. The excavation was then completed, and the specimen was removed from the outcrop. Specimens were carefully protected and transported to the Dinosaur Science Museum and Research Center in Texas where preparation and curation took place. The GPS and photographic data were subsequently processed and used to generate virtual 3D maps of the quarries, illustrating the position of each bone in plan view [14]. Much of the subsequent analysis is based on these maps. Because we can view each quarry as an independent object, search for taxa as well as other features, and generate a display of the virtual quarry map with the selected items highlighted [15], it is relatively straightforward to show the photographic positions of every bone from any selected taxon. These quarry maps were then used to extract positional data for each taxon. All quarry maps and individual specimen data and metadata are accessible at https://fossil.swau.edu.

The stratigraphic framework of the quarries along the strandline transect was developed through detailed lithofacies descriptions, and measured using high resolution RTK GPS [14]. The seven sections were then correlated across a ~2.5 km NW–SE transect in the Lance Formation.

For bone distribution diagrams, the azimuth of the long axis of each long-bone (l:w > 2) was read from reconstructed quarry diagrams using a protractor. Orientations were subsequently grouped into 24 equal 15° bins for circular statistical analysis. Because bone long axes are bidirectional (axial data), orientations were analyzed using the double-angle transformation method prior to application of the Rayleigh test [16]. The known paleocurrent direction, inferred from independent sedimentary structures, was toward the southeast. This information was used to resolve the 180° ambiguity inherent in the axial data to determine the most likely down-current flow direction.

No permits were required for the described study, which complied with all relevant regulations. Contracts between the land owners and Southwestern Adventist University are on file at the university.

Results - taphonomy

Triceratops2 Quarry

This quarry contained the remains of four distinct dinosaur individuals of four different taxa in various states of disarticulation and disassociation, in addition to other non-dinosaurian taxa (Fig 4). All bones with distinctively elongate axes (L/W >2, in this case 80 specimens) were plotted in a rose diagram (Fig 5A). The general axes of elongate bones in this quarry were west and east, although the direction of dispersion of the bones in the quarry itself is northwest to southeast. The remains of each taxon are detailed below in separate diagrams. The elevations (in meters above sea level) of some bones are given in parentheses. These numbers are a mean of several readings taken on different locations on each bone.

Fig 4. Quarry diagram for Triceratops2 Quarry with all bones represented. *Edmontosaurus* bones depicted in yellow, *Triceratops* bones in green, *Thescelosaurus* bones in red, Anzu bones in blue. North is up.

Fig 5. Rose diagrams for orientation of elongate bones in all quarries. A - E) Triceratops2 Quarry **A**) all bones. **B**) *Triceratops* bones. **C**) *Anzu* bones. **D**) *Thescelosaurus* bones. **E**) *Edmontosaurus* bones. **F**) Triceratops1 Quarry. **G**) Atullah2 Quarry. **H**) JW Quarry. **I**) Triceratops4 Quarry. **J**) Rabbit Ears Quarry. **K**) Triceratops5 Quarry. **L**) Composite of all quarry diagrams (B-K). N gives the number of measurements. Z gives the Raleigh statistic. P gives the probability.

Triceratops

A mostly intact, large, chasmosaurine ceratopsid skull (HRS 15437) and associated postcrania are referred to the genus *Triceratops* based on their presence in the Lance Formation and the lack of fenestrae in the parietals (distinguishing it from *Torosaurus*). This specimen can be further referred to *Triceratops horridus* rather than *Triceratops prorsus* based on its shallow and long rostrum and its short nasal horncore [17].

The skull was found inverted with the brow horns pointing in a northeasterly direction (Fig 6, bottom center, Fig 7). The elevation of the lowest part of the skull was 1235.2 meters. The disarticulated left squamosal (1235.4) was above and in contact with the parietal region of the skull. The right squamosal (1235.5) was displaced a meter to the south at the same elevation as the top of the skull. The right maxillary (1235.0) was located three meters northwest of the skull. The left maxillary (1235.4) was 0.3 meters to the east of the skull. Neither the maxillaries nor the dentary possessed any teeth. The left dentary fragments (1235.5) were four meters due north of the skull. Two cervical, five dorsal and one caudal vertebrae were recovered (all between 1235.4 and 1235.7) spread out east-northeast of the skull (Fig 6). Only two partial ribs attributed to *Triceratops* were found, and none of the chevrons in the area were attributed to *Triceratops* since they were all associated with the *Edmontosaurus* tail mentioned below.
A single *Triceratops* femur, tibia, pubis, and one metatarsal (all 1235.1), presumably from the same animal, were one to two meters northeast of the skull. These bones, like the skull itself, were deposited with long axes aligned with an east-northeast to west-southwest direction. A second metatarsal (1234.8) was a meter north of the skull and a third (1235.1) was three meters east of the skull. The trend of the 34 elongate bones across the quarry is generally along a west-northwest to east-southeast line (Fig 5B).

Fig 6. Triceratops2 Quarry showing only bones from *Triceratops*. North is up.

Fig 7. Triceratops2 Quarry showing *Triceratops* bones in skeleton. Left side illustrated.

Anzu

These remains are the first we have found unambiguously attributable to the caenagnathid oviraptorosaur, *Anzu wyliei* [18], as demonstrated by the rugosity in middle of the anterior surface of the astragalocalcaneum (HRS 15439), which is an autapomorphy of *A. wyliei* [19]. The astragalocalcaneum fits well on the tibia, and the fibula, metatarsals, and phalanges are all similarly-sized and not duplicated, suggesting these bones came from the same individual of *Anzu wyliei*. These remains will be described further in a future publication. No cranial material from *Anzu* has yet been recovered. Two caudal vertebrae were recovered, the first (1234.9) two meters north and the second (1235.2) 3.5 meters north of the *Triceratops* skull. A partial radius (1235.0) was 5.5 meters north and six meters west of the *Triceratops* skull and a partial metacarpal (1234.9) was one meter northwest of that. The largest bone from *Anzu* recovered was a tibia (1235.1) two meters west of the *Triceratops* skull (Fig 8). At the same elevation and another meter west-southwest was the delicate but intact fibula. Two metatarsals (1235.0, 1235.1) were three meters south and six and five meters west from the skull. The astragalocalcaneum (1235.1) was 30 cm east of the tibia. A pedal phalanx was in contact with the top of the inverted *Triceratops* skull (1235.6), another (1235.4) was two meters east of the *Triceratops* skull and a third (1235.6) was two meters farther east from that and a pedal claw (1235.4) another meter east from that. A hallux (1235.3) was two meters north-northeast of that. An as-yet unidentified fragment (1235.3), likely attributable to *Anzu*, was located 12 meters north and four meters west of the *Triceratops* skull. Figure 9 illustrates the skeletal recovery.

Although the remains of this taxon were scarce compared to the others, those bones with elongate axes (10) were arrayed in a west-southwest-east-southeast pattern (Fig 5C).

Fig 8. Triceratops2 Quarry showing only bones from *Anzu*. North is up.

Fig 9. Triceratops2 Quarry showing *Anzu* bones in skeleton.

Thescelosaurus

We refer this specimen to *Thescelosaurus* based on the presence of frontals wider at the midorbital level than across the posterior end (HRS 15489), which is recognized as an autapomorphy of the genus [20,21]. A future publication will describe the specimen in greater detail.

On the Hanson Ranch generally, *Thescelosaurus* is a rare taxon. In 29 years of excavation, only 46 bones and teeth ~~are~~ attributable to *Thescelosaurus* have been excavated apart from the specimen described below (http://fossil.swau.edu). This particular specimen was first encountered at Triceratops2 Quarry in 2014 while excavating the *Triceratops* skull (Figs 4,10). A femur, a pedal phalanx, and three caudal vertebrae were uncovered three meters east of the *Triceratops* skull and at the same elevation (1235.5). The following season, a large portion of a *Thescelosaurus* skeleton was uncovered in a one square meter area just north of the femur and at approximately the same elevation. Fifteen disarticulated skull pieces were found in close association under the mostly articulated pelvic bones. Forty associated vertebrae (dorsal and caudal) were found directly to the north and 15 ribs (six right, eight left, one indeterminate) were scattered nearby. Pelvic appendages were more completely represented than forelimbs, and proximal elements were better represented than distal elements. The sacrum, right ilium, right and left ischia, pubes, and femora, a left tibia, and fibula, three metatarsals, thirteen pedal phalanges and three pedal unguals represented the hindlimbs and a left scapulocoracoid and a

possible radius represented the forelimbs. A total of 118 elements were recovered (Fig 11). One particularly vexing challenge was the variable matrix. Part of the bones occurred in sandy matrix, and these were quite fragile, whereas others were encased in an indurated carbonate cemented sandstone. These bones often required work with an air chisel to disengage them from the matrix. During subsequent seasons, as the site was expanded, other scattered *Thescelosaurus* elements were exposed. In 2016, a third, larger pubis (1235.8) was uncovered five meters east and two meters south of the *Triceratops* skull. It is clear that the tightly clustered remains belong to a single individual, but this third larger pubis indicates that more than one individual is represented in this site. While the bone distribution for *Thescelosaurus* cluster does not give much directionality, the overall distribution of bones with elongate axes (Fig 5D) is similar to that of other specimens in this quarry.

Fig 10. Triceratops2 Quarry showing only bones from *Thescelosaurus*. North is up.

Fig 11. Triceratops2 Quarry showing *Thescelosaurus* bones in skeleton. Left side illustrated.

Edmontosaurus

It was not until our third season of excavation here (2016) that the first bones of *Edmontosaurus* were recovered from the site (Fig 12). We refer this specimen to *Edmontosaurus* because it has a circular distal blade of the pubis (HRS 20951) that is much more expanded ventrally than dorsally [22]. Because *E. annectens* is distinguished from *E. regalis* morphologically by cranial bones [22], we rely on the stratigraphic position in the Maastrichtian to refer this specimen to *E. annectens*. No cranial bones from *Edmontosaurus* were recovered. We excavated 65 articulated caudal vertebrae (1235.6) from a region six to eight meters north of the *Triceratops* skull and at the same elevation. Twenty-nine chevrons, four ribs (1235.4, 1235.2, 1235.2, 1235.4), and numerous tendons were also excavated. Two scapulae (both 1234.8), a

single humerus (1235.2), radius (1235.5), and clavicle (1234.8) were also recovered. Subsequent expansion of the quarry yielded two each of ilia (1235.2, 1235.5), ischia (1235.2, 1235.5), and pubices (1235.2, 1235.5). A single tibia (1235.0) was also recovered. One pubis was positioned vertically in the sandy sediment, without any obvious evidence for trampling on the bone itself or in the associated bones. The *Edmontosaurus* humerus (1235.1) was recovered 0.5 m east of the *Triceratops* skull. A third *Edmontosaurus* pubis (1235.7) was excavated eight meters east of the *Triceratops* skull at the easternmost boundary of the quarry, so the remains of more than one animal are represented here, just as was the case for the *Thescelosaurus*. However, the distribution of bones with the closely associated pelvic bones and the assemblage of caudal vertebrae nearby support the assertion that the bulk of the *Edmontosaurus* bones represent one specimen (Fig 13), and that it remained closely associated during transport and deposition. Excavation of the area surrounding these bones was continued for a minimum of two meters laterally to determine whether there could be more of the skeleton present. The general pattern of bone orientation for elongate bones of this specimen (Fig 5E) is northwest to southeast, paralleling the distribution of the bones across the quarry.

Fig 12. Triceratops2 Quarry showing only bones from *Edmontosaurus*. North is up.

Fig 13. Triceratops2 Quarry showing *Edmontosaurus* bones in skeleton. Left side illustrated.

Testudines

The distribution of turtle remains is indicated in Figure 14. Many of the bones are isolated pieces of plastron or carapace, but one nearly intact plastron (1235.6) and several turtle limb bones (1235.5, 1235.6) were among the recovered remains. The orientation of the bones echoes other trends we are seeing in this quarry, generally along a northwest-southeast line (Fig 14).

Fig 14. Triceratops2 Quarry showing only bones from Testudines. North is up.

Triceratops1 Quarry

This site is located 230 meters southeast from Triceratops 2. It was recognized by the presence of a protruding brow horn of a ceratopsid cranium (Fig 15). Because it was at the top of a ridge, only limited excavation could be carried out. In addition to the partially complete skull with a single brow horn oriented to the southwest, a premaxilla, jugal, parietal, and dentary (all 1243.2) and several ceratopsid bone fragments were recovered (Fig 16). The few bones recovered with elongate axes (6) were directed toward the northwest and southeast (Fig 5F). Excavation of the two-meter area surrounding the skull did not yield additional bones, reinforcing our conviction that we recovered all that remained at the site.

Fig 15. Triceratops1 Quarry showing all bones recovered. North is up.

Fig 16. Triceratops1 Quarry showing *Triceratops* bones in skeleton.

Atullah2 Quarry

Another quarry site was opened 90 meters east and 75 meters south from Triceratops2 Quarry. This site (Fig 17) has revealed the partially articulated remains of an *Edmontosaurus* skeleton. Aside from a couple of as-yet unidentified skull fragments, cranial elements were absent. There were two disarticulated (1241.2, 1241.3) and nine associated dorsal vertebrae (1241.1 – 1241.2) with most of the ribs (9 right, 5 left) in proximity. Nine caudal vertebrae (1241.0 – 1241.3) and nine chevrons (1240.9- 1241.2) were recovered (Fig 18). From the pectoral girdle, one radius (1241.1) and one metacarpal (1241.1) and from the pelvic girdle, an Ilium (1241.3), ischium (1241.1), pubis (1241.2), right and left femora (1241.1), a right metatarsal and a pedal phalanx were recovered. According to the ranch owners, the area around these remains has served as a source of eroding bones which were given away as souvenirs. We

can therefore assume much more of the skeleton was originally present. Initially, it seemed plausible that this specimen could represent the body axis of the *Edmontosaurus* in Triceratops2 Quarry, but the presence of redundant pelvic elements countered that possibility. When all bones with a detectible long axis were plotted on a rose diagram (Fig 5G), the directionality was northwest and southeast.

Fig 17. Atullah2 Quarry showing all bones recovered. North is up.

Fig 18. Atullah2 Quarry showing *Edmontosaurus* bones in skeleton. Left side illustrated.

JW Quarry

This fourth quarry is just over two kilometers northwest of Triceratops2. JW Quarry (Fig 19) adds significant taphonomic data relevant to the process of disarticulation and transport of the remains of individual specimens. In this site, the disarticulated remains of a single *Triceratops* were uncovered. The inverted skull (1200.0), including the braincase and brow horns in the upper left of Figure 19, was surrounded by a mass of bones presumed to be from the same animal in an area of about 16 square meters. The horns were oriented in a northeasterly direction. Additional cranial elements recovered include right and left jugals (1200.3, 1200.5), a predentary (1200.6), a right quadrate (1200.3), a quadratojugal (1200.8), a lachrymal, right and left squamosals, and a toothless dentary. The spinal column was represented by the syncervical (1200.5). eight dorsal vertebrae (1200.4 – 1200.8), ten left and eight right ribs (1200.1-1200.9), and 22 partial ribs. As was the case at the other Triceratops quarries, there were few limb bones and tail bones, but other parts of the skeleton were well represented. These included right and left scapulae and a right coracoid (all 1200.5) from the pectoral girdle and the sacrum (1200.4), an ilium (1200.8), and the right pubis (1200.7) from the pelvic girdle. The left femur (1200.2), an astragalus, one possible phalanx (both 1200.4) were also recovered. Continued excavation at this

site suggested most bones have been recovered (Fig 20). The long axes of elongate elements (43) trends northeast-southwest (Fig 5H).

Fig 19. JW Quarry showing all bones recovered. North is up.

Fig 20. JW Quarry showing *Triceratops* bones in skeleton. Left side illustrated.

Triceratops4 Quarry

This quarry, discovered in 2021 revealed additional taphonomic data from disarticulated but associated *Triceratops* remains (Fig 21). Excavation was halted in 2024 because the site is up against a high bank, and further excavation was deemed impractical. Thus far the only cranial elements recovered were an occipital condyle (1238.3), a squamosal and a single epoccipital. The vertebral column is represented by three dorsal vertebrae (1238.4), one possible cervical vertebra (1238.3), two centra, 23 ribs (three left, four right and 16 partials, all 1238.3), and one chevron (1238.2). The pectoral girdle includes a right scapula (1238.5) and a right metacarpal (1238.3). The pelvic girdle was represented by a left pubis (1238.4), paired right and left ischia (1238.3), a left ilium (1238.2), right and left femora (1238.1, 1238.3), a right tibia and fibula (1238.4, 1238.3), and a metatarsal (1238.4), all presumably from the same *Triceratops* (Fig 22). Thus far, the large majority of the elongate bones (23) appear to be oriented in east-west and northeast-southwest directions (Fig 5I).

Fig 21. Triceratops4 Quarry showing all bones recovered. North is up.

Fig 22. Triceratops4 Quarry showing *Triceratops* bones in skeleton. Left side illustrated.

Rabbit Ears Quarry

This quarry, also encountered in 2021, was explored at the surface and has not yet been excavated. All the dinosaur bones identified at this site (seven of 12 total) were field identified as either frill or bone fragments consistent with *Triceratops* (Fig 23). Bone elevations ranged from

1208.5 to 1208.7 meters. The elongate bones (6) had an orientation of northwest to southeast (Fig 5J).

Fig 23. Rabbit Ears Quarry showing all bones observed. North is up.

Triceratops5 Quarry

This site was discovered in 2021 during reconnaissance (Fig 24). The initial indication of bone was a partially exposed *Triceratops* squamosal. Further exploration of the area in June 2022 revealed several more pieces of frill and two occipital condyles. Approximately 25 meters to the SW from the initial discovery site, another site was located which quickly revealed a scapula with associated coracoid, several dorsal vertebrae, and assorted pieces of frill and tendons which were removed in 2022. The following three seasons, excavation continued on this site, revealing over 200 bones of *Triceratops* including many fragments of frill, an intact braincase with occipital condyle (1223.8), left and right squamosals (1223.6, 1223.8), a right lachrymal (1223.6), a maxillary (1223.9), right and left dentaries (1223.8, 1223.9) and a horn core (1223.9). The axial skeleton included the syncervical (1223.9), a total of thirteen dorsal vertebrae (1223.7-1223.9), a hyoid (1223.7), a chevron (1223.6), twenty-six mostly intact ribs (10 right and 16 left, all 1223.7-1223.9) and additional rib fragments. The pectoral girdle included the left scapulocoracoid (1224.6) and right coracoid (1224.8), and a humerus (1223.3). The pelvic girdle included a sacrum (1223.7), right and left pubes (1223.7), a right ischium (1223.7), and two astragali (1223.6, 1223.8), all from *Triceratops* (Fig 25). Although this site has been mostly excavated, there are nearby locations with Triceratops bones that are yet to be explored. The ribs lie mostly parallel to each other and to the femur in a northwest to southeast orientation. The directional disposition of the bones excavated so far is shown in Fig 5K.

Fig 24. Triceratops5 Quarry showing all bones recovered. North is up.

Fig 25. Triceratops5 Quarry showing *Triceratops* bones in skeleton. Left side illustrated.

Results - stratigraphy

This portion of the Lance Formation reflects depositional conditions near the eastern shoreline of the Western Interior Seaway during the upper Maastrichtian [5]. Athough stratigraphic resolution in the Lance is often hindered by weathering and limited outcrop continuity, we identified three regionally correlative sedimentary cycles—designated Cycle 1(C1), Cycle 2(C2), and Cycle 3(C3)—which can be consistently traced across all quarries (Fig 26) constrained by continuous red carbonate cemented sandstones.

Fig 26. Stratigraphic sections taken near six of the quarries, and a control section taken nearby. The carcasses appear at several horizons within a single cycle.

Cycle 1

Cycle 1 forms the basal part of all measured sections and is characterized by a dominance of purple mudstones and grey siltstones interbedded with thin to medium-bedded very fine- to coarse-grained sandstones. This cycle shows the highest number of preserved vertebrate fossil localities, with most bone accumulations embedded in purple massive mudstone facies, particularly in Triceratops4 and Triceratops5 quarries, which most likely represent the same stratigraphic horizon. Occasionally, these sediments also contain fossil plant macerals. Similar stratigraphic alignment is observed between Triceratops1, Atullah2, and possibly JW Quarry, although this latter correlation remains tentative due to distance (2.25 km) and lack of high-resolution control at this scale within the Lance Formation.

A distinctive middle sandstone bed within C1, yellow in color and occasionally showing carbonate-cemented current ripples, can be correlated across multiple sections. This bed thins toward the southeast, appearing nearly absent at Triceratops4, and thickens again toward

Triceratops5 and the Control Section. This stratigraphic behavior suggests lateral wedging, potentially related to channel migration or bar accretion in a fluvial to deltaic setting. Notably, Triceratops2 Quarry is the only site whose fossil remains were hosted within this sandstone rather than in the bounding purple mudstones.

Below Cycle 1, similar facies assemblages—mudstones interbedded with ripple-laminated sandstones—persist, suggesting continuity in depositional processes prior to the base of the measured section. For a proper analysis of this main cycle and all sections, the first laterally consistent red cemented sandstone was arbitrarily selected as a sequence base level. Petrographic analysis of the two resistant sandstones at the top and bottom of Cycle 1 revealed plutonic and volcanic rock fragments, suggesting input from a terrestrial source to the west. The samples were cemented by secondary carbonate cement but lacked the presence of any primary carbonate grains. The samples also contained glauconite grains and glauconitic cement which implies a marine influence [23]. These factors along with regional paleogeographic reconstructions (Fig 26), have allowed us to suggest a marginal marine environment for the deposition of Cycle 1.

Cycle 2

Unlike Cycle 1, Cycle 2 lacks any known fossil quarries, but the beds retain value for correlation since they exhibit little lateral facies change and moderate thickness variations. Cycle 2 is composed of interbedded very fine- to medium-grained sandstones with parallel laminations, and reduced percentage of purple mudstones and thinner bedding overall. Scattered dinosaur bone fragments were occasionally encountered in the mudstones. These facies may represent higher-energy overbank or levee deposits.

Cycle 3

Cycle 3, like Cycle 2, contains no known fossil quarries, but ~~does~~ preserve the same lithological vertical facies arrangement, dominated by purple mudstones containing plant remains and very fine tan sandstones. This cycle is capped by a conglomeratic unit that serves as a reliable regional marker bed, easily distinguishable by its carbonate mud clast-supported fabric, erosive base, and interbedding medium sandstone. This conglomerate is clearly exposed in Triceratops1, Triceratops2, and the Control Section, and has also been identified in smaller, unmapped outcrops between them. This peculiar bed likely marks a critical shift toward higher-energy proximal shoreline migration.

An additional notable feature throughout the whole succession~~,~~ is the continuity of the carbonate-cemented, red-colored sandstones with trough crossbedding. Although the carbonate cementation likely reflects diagenetic overprinting, the lateral continuity, consistent stratigraphic position, and common association with soft-sediment deformation suggest these beds can be confidently used as secondary correlation markers, likely reflective of episodic events that establish a time constraint.

Stratigraphic and Depositional Trends

Despite the lateral distance, these facies can be continuously correlated across all sections, with only thickness variations observed, implying minimal lateral facies change and supporting deposition in a relatively low-gradient, laterally extensive floodplain or interdistributary deltaic environment. These thickness differences may be the result of localized sedimentologic controls, such as point bar–floodplain interactions, channel avulsion, or crevasse splay progradation. Further paleocurrent analysis on the abundant current ripple lamination structures is required to refine this interpretation.

The vertical stacking of facies across the sections displays a tentative coarsening-upward trend, consistent with the increasing energy levels at the top of each cycle. This is reflected in the pattern of current ripple lamination to wavy bedding to convolute bedding, consistent with a pro-gradational depositional regime. While this model requires further corroboration through additional stratigraphic and sedimentologic analyses along the Lance exposures at the Hanson Ranch, it fits with gradual shoreline progradation during regression of the Western Interior Seaway.

The stratigraphic correlation was constructed along a NW–SE transect, matching the taphonomic alignment of quarries and long bone orientations (~140° azimuth). Continuous stratigraphic tracing is difficult due to deep weathering, poor outcrop exposure, and limited resolution, particularly at Rabbit Ears Quarry, which remains unexplored. However, the well-preserved profiles allow us to interpret a continuous sedimentary succession with strong lateral connectivity of facies, minimal facies change, and localized variations in bed thickness.

Discussion

Description of the Quarries

Quarries in the main bonebed a kilometer east of the quarries described above, tend to yield an abundance of *Edmontosaurus* bones, generally thoroughly disarticulated [5]. Other outlying quarries [6] yield mostly bones of dinosaur taxa other than *Edmontosaurus*, completely disarticulated, and rarely associated, along with the remains of turtles, crocodiles and fish, taxa infrequently encountered in the main bonebed. Of the quarries that are the subject of this report, Triceratops2 is unique in representing four dinosaurian taxa, all partially disarticulated, in a small area. The other six quarries described above are limited to articulated and associated bones from usually a single *Triceratops* or *Edmontosaurus* (Atullah2 Quarry) specimen. These quarries

also may contain disarticulated turtle or crocodile remains. The bones occur in what appears to be a single horizon in each quarry, and the long axes of bones are distributed close to the overall trend of the quarries themselves.

Degrees of Disarticulation

In Triceratops2 Quarry, a variety of different modes of association of bones were encountered, from completely disarticulated and only loosely associated (*Anzu*), to completely disarticulated, but generally associated (*Triceratops*), to mostly disarticulated, but tightly associated (*Thescelosaurus*), to significantly articulated and associated (*Edmontosaurus*). Although this wide variation in states is informative, it almost necessitates a somewhat different history for each specimen. However, differering amounts of decomposition due to varying time since death could result in slightly different distribution patterns with bones in the more intact carcass, (Thescelosaurus), remaining together and the more decomposed carcasses spreading out due to wave action. A shoreline irregularity could explain the rich accumulation of carcasses in one place, and result in the wider range of bone orientation for this particular quarry. The overall pattern of alignment of bones in the quarry suggests transport of some sort is a part of the explanation, but what mechanism of transport can keep the bones of each taxon together during transport?

Several authors have proposed bloating and floating for dinosaurs found in marine deposits [24-26] and for the frequent discoveries of upside-down ankylosaurs [27,28]. Bloating and floating has been observed in many extant marine vertebrates, with the mandibles and head detaching from the rest of the carcass [29]. Schäfer [29] found from experiments with seal decomposition that the lower jaws detached after 36 days of floating at the surface, and by 41 days "the carcass is still afloat but it now resembles a loose sack" (p. 33). After 48 days, Schäfer

[29] noted that the body was open at both ends, but that the vertebral column, rib cage, shoulder blades, pelvis, and thighs were still in the floating sack of skin. Brand et al. [30] with lizards, and Syme and Salisbury [31] with crocodiles, have shown that modern reptiles, allowed to decay in water, are also surrounded by a tough sack of skin that can retain the bones of the creature for extended periods after death.

Although dinosaurs like *Triceratops* and *Edmontosaurus* are orders of magnitude larger than lizards, the similar decay in floating seals and extant reptiles suggests that encapsulation and transport of bones in a sack of skin is a realistic possibility to explain some of the taphonomy encountered. Crocodiles [31], lizards [30], and seals [29] initially sank to the bottom after death, then rose and floated at the surface for a protracted period before coming to final rest on the bottom. During the interim, the floating sac of dermis/epidermis tended to keep the bones together as disarticulation ensued. Since both Brand et al. [30] and Syme and Salisbury [31] documented the loss of bones from the dermal/epidermal sac during transport and/or flotation, a similar package of dinosaur remains could be delivered to the site of ultimate burial in a variety of different states, as argued for varying taphonomic modes of dinosaur fossils in marine deposits in Eastern North America [24]. The common occurrence of dinosaur specimens lacking skulls [32] matches well with taphonomic observations of floating carcasses losing heads before elements of the torso [29,30]. The loss of the skull would leave a tear in the dermal sack. This hole may have opened the door to rapid loss of cervical vertebrae in floating dinosaur carcasses, which would explain the absence of cervical vertebrae from all seven sites. The absence of caudal vertebrae from all sites except for the mostly intact *Edmontosaurus* tail found in Triceratops2 and a few proximal caudals from the *Edmontosaurus* at Atullah Quarry, can be explained by the early loss of tails prior to stranding and burial.

Loss of Teeth from Jaws

In four quarries a total of nine dentaries and three maxillaries were identified. Eight of these were from *Triceratops*. The remaining dentary was from *Thescelosaurus*. All the dentaries and maxillaries were within meters of the skulls, yet none of them contained teeth. This would suggest that the loss of teeth from these skulls may have occurred during the decomposition of the skeleton prior to transport, rather than during any transport that may have accompanied burial, since the teeth were not generally found with the skeletons [33]. This is what Brand et al. [30] found for modern mammal teeth and Syme and Salisbury found for crocodiles [33]. Teeth were often lost from jaws early, especially when the bones were in water. Similar tooth loss is commonly observed in the jaws of ornithopod [34] and theropod [35] dinosaurs, where the rapid decay of periodontal ligaments is linked to the rapid postmortem tooth loss. The rarity of cracks on isolated teeth that were found is consistent with disarticulation occurring in water [30].

Direction of Transport

The bones in all seven quarries give directional data for transport that can be useful for reconstructing the taphonomic history of the deposits. Although long bones give reliable orientation data, they rarely give sense of the direction of transport. We have assumed transport was toward the southeast because both local and regional paleocurrent data derived from crossbedding gives that sense [36,37], as do the distributions of bones across the quarry sites. With that assumption, elongate bones from all seven quarries align toward the east or southeast. This suggests that the bones/carcasses were in water prior to deposition and that southeasterly flowing water currents were involved in the deposition.

Of further interest is the observation that all seven quarries, the only quarries we have opened on this Ranch that have significant associated remains~~,~~ lie on or near a line that also

trends toward the southeast at 140 degrees (Fig 15). This alignment is consistent with a shoreline accumulation of floating carcasses, since it roughly parallels the expected shoreline of the WIS (Fig 3), and we believe that this linear feature identifies a transient Upper Cretaceous shoreline along the shore of the WIS. Strandlines and strandline deposits have long been recognized as important elements in environmental interpretations [38]. But what criteria are used to determine the position of a strandline? Sometimes the term is used generically to represent what is believed to be a high-stand (or low-stand) deposit or surface during a transgressive (or regressive) event [39]. Sometimes specific kinds of deposits such as pebble beds have been identified as relict strandline deposits [40,41]. Specific physical characteristics of strandlines themselves have also been recognized. For example, Binns [42] identifies what he assumes to be physical strandlines by the presence of pumice drift accumulations in Quaternary sediments in Great Britain. Dinosaur trackways have also been used to define strandlines [43,44].

Longshore drift can explain the similarity of the long bone orientation as the remains were liberated from the rotting stranded carcass and moved laterally along the shoreline [45-47]. Bones aligned in nodules contributed to identifying a strandline deposit in Argentina [48]. Rogers and Kidwell [46], Varricchio [49], and Tasch and Zimmerman [50] all suggest aligned bone concentrations are consistent with a strandline origin. The Tendaguru deposits of Tanzania contain the associated, but largely disarticulated bones of sauropods, in much the same state as the bones described in this report [51-53]. These deposits are described as strandline deposits. The bodies of the dinosaurs were transported from the place of death to the site of burial by water. Weigelt [54] identifies massed bone deposits as strandline accumulations. In Weigelt's case and others, the alignment is the definitive factor warranting a strandline designation.

Timing of Events

We would expect that skeletal remains at these seven sites were originally at similar elevations if they comprised a single strandline deposit. The recovered bones range in elevation from 1240 meters in the east to 1200 meters in the west. However, the quarries lie along the eastern limb of a north-south structural basin. Since the present dip of this arm of the basin is 3 - 5 degrees toward the west, we would expect that sites that were the farthest west (JW and Rabbit Ears) would be at a lower elevation than those farther east, just as we observe. Thus, the elevations that we find are consistent with a series of strandings closely related in time. All of the bonebed-containing facies appear to be within a single coarsening-upward cycle (C1), allowing the possibility that these remains record related stranding events.

Three of the quarries considered in this paper (Rabbit Ears, Triceratops4, and Triceratops5) were originally located by testing this strandline hypothesis and walking out the ancient shoreline in search of additional sites. It will be interesting to see what further discoveries can be made by continuing to search along this trajectory.

Conclusions

Along a 2.5 km northwest-southeast line, seven apparently related taphonomic quarry sites in the Lance Formation of NE Wyoming have each produced articulated and associated bones of one or more individuals. Predictive exploration along this presumed strandline enabled ~~us to~~ discover three of the seven quarries. They predominately contain remains of *Triceratops*, but also include *Edmontosaurus*, *Thescelosaurus*, *Anzu*, and non-dinosaurian remains including turtles and crocodilians. We interpret these as disarticulated bloated carcasses partially held together by a tough semi-intact dermis, with the loss of some elements prior to deposition. The loss of teeth from the jaws is consistent with modern mammal and crocodile disarticulation in water [30,31] and expected from the rapid decay of the periodontal ligaments during flotation of

the carcasses. The general orientation of long bones from each of these sites is from the NW to SE (approx. 140 degrees), matching the orientation of the quarries and the proposed strandline. Longshore drift provides a reasonable explanation for the similarity of long bone orientation. This mirrors local and regional paleocurrent data suggesting stranding of floating carcasses on a shoreline of the easterly regressing Western Interior Seaway.

Acknowledgements

We thank all the diligent and committed quarry members for their help with data collection. We acknowledge Southwestern Adventist University and Southern Adventist University for institutional support and access to facilities and resources essential for this work. We also thank the owners of the Hanson Research Station for helping us like family and facilitating this research.

References

1. Hatcher JB, Lull RS. The Ceratopsia. Monographs of the US Geol Surv. 1907;49:1–198.
2. Marsh OC. Geological horizons as determined by vertebrate fossils. Amer J Sci. 1891;42:336–8.
3. Hatcher JB. Relative age of the Lance Creek (Ceratops) beds of Converse County, Wyoming, the Judith River beds of Montana and the Belly River beds of Canada. Amer Geol. 1903;31:369–75.
4. Cobban WA, Reeside Jr JB. Correlation of the Cretaceous formations of the Western Interior of the United States: Geol Soc Amer Bul. 1952;63:1011–44.

5. Snyder K, Mclain M, Wood J, Chadwick A. Over 13,000 elements from a single bonebed help elucidate disarticulation and transport of an *Edmontosaurus* thanatocoenosis. PLOS One. 2020;15:e0233182. https://doi.org/10.1371/journal.pone.0233182.

6. Mclain MA, Ullmann PV, Ash RD, Bohnstedt K, Nelsen D, Clark RO, Brand LR, Chadwick AV. Independent confirmation of fluvial reworking at a Lance Formation (Maastrichtian) bonebed by traditional and chemical taphonomic analyses. Palaios, 2021;36:193–215. https://doi.org/10.2110/palo.2020.064.

7. Sereno PC, Saitta ET, Vidal D, Myhrvold N, Ciudad Real M, Baumgart SL, Bop LL, Keillor TM, Eriksen M, Derstler K. Duck-billed dinosaur fleshy midline and hooves reveal terrestrial clay-template “mummification”. Science. 2025;391(6780):eadw3536.

8. Mclain MA, Snyder K, Chadwick AV. Discovery of a unique multitaxic dinosaur bonebed from the Lance Formation (Maastrichtian) of Wyoming. Geol Soc Amer. 2017;10:1130.

9. Committee WGATS. Geologic history of Powder River Basin. Amer Assoc Petrol Geol Bul. 1965;49:1893–1907.

10. Ullmann PV, Caputo C, Snyder K, Chadwick A, Ash RD. Trace element taphonomy of the Hanson Ranch *Edmontosaurus* Bonebed supports its origin via transportation of a mass death assemblage. Chem Geol. 2025;672:122501. https://doi.org/10.1016/j.chemgeo.2024.122501.

11. Cobban WA, Merewether EA, Fouch TD, Obradovich JD. Some Cretaceous shorelines in the western interior of the United States. In: Caputo, M.V., Peterson, J.A., Franczyk, K.J. (Eds.), Mesozoic Systems of the Rocky Mountain Region, USA. Rocky Mount Sec SEPM. 1994;393–413.

12. Roberts LN, Kirschbaum MA. Paleogeography of the Late Cretaceous of the Western Interior of middle North America: Coal distribution and sediment accumulation. US Government Printing Office; 1995.

13. Lillegraven JA, Ostresh LM. Late Cretaceous (earliest Campanian/Maastrichtian) evolution of western shorelines of the North American Western Interior Seaway in relation to known mammalian faunas. In: Brown TM, Rose KD, editors. Dawn of the Age of Mammals in the northern part of the Rocky Mountain Interior, North America; GSA Special Papers; 1990, pp.1-30.

14. Chadwick AV, Silver MP, Turner L, Woods J. The application of digital reconstruction techniques in taphonomy of an Upper Cretaceous dinosaur site in eastern Wyoming. J Taph. 2016;14:1–14.

15. Woods JA, Chadwick AV. Development of an on-line database with GIS connections for vertebrate and other fossils. Proc 40th Geosci Infor Society. 2005;36:53–69.

16. Mardia KV, Jupp PE. Directional Statistics. Chichester: John Wiley & Sons; 2000.

17. Mallon JC, Roloson M, Bamforth E., Scannella JB, Ryan MJ. The Canadian fossil record supports anagenesis in *Triceratops* (Ornithischia, Ceratopsia). Can J Earth Sci. 2025;62:1222–36.

18. Frederico CA, Mclain M. New Caenagnathid (theropoda: Oviraptorosauria) material from the Lance Formation (Maastrichtian) of Wyoming. Geol Soc Amer Abstr. 2021;53:370156.

19. Lamanna MC, Sues HD, Schachner ER, Lyson TR. A new large-bodied oviraptorosaurian theropod dinosaur from the latest Cretaceous of western North America. PLOS One. 2014;9:e92022.

20. Boyd CA, Brown CM, Scheetz RD, Clarke JA. Taxonomic revision of the basal neornithischian taxa *Thescelosaurus* and *Bugenasaura*. J Vert Paleo. 2009;29:758–70.

21. Boyd CA. The cranial anatomy of the neornithischian dinosaur *Thescelosaurus neglectus*. PeerJ. 2014;2:e669.

22. Hai X, Zhao X, Wang K, Li D, Chen S, Mallon JC, Zhang Y, Xu X. Comparative osteology and phylogenetic relationship of *Edmontosaurus* and *Shantungosaurus* (Dinosauria: Hadrosauridae) from the Upper Cretaceous of North America and East Asia. Acta Geol Sinica. 2014;88:1801–40.

23. Cloud Jr PE. Physical limits of glauconite formation. AAPG Bulletin. 1955 Apr 1;39(4):484-92.

24. Schwimmer DR. Late Cretaceous dinosaurs in eastern USA—A taphonomic and biogeographic model of occurrences. In: Dinofest International, Philadelphia: Academy of Natural Sciences; 1997. p. 203–11.

25. Schwimmer DR, Stewart JD, Williams GD. Scavenging by sharks of the genus *Squalicorax* in the Late Cretaceous of North America. Palaios. 1997;12:71–83.

26. Everhart MJ, Ewell K. Shark-bitten dinosaur (Hadrosauridae) caudal vertebrae from the Niobrara Chalk (Upper Coniacian) of western Kansas. Trans Kan Acad Sci. 2006;109:27–35.

27. Sternberg CM. Comments on dinosaurian preservation in the Cretaceous of Alberta and Wyoming. National Museums of Canada Pub in Palaeontology. 1970;4:1–9.

28. Mallon JC, Henderson D, McDonough CM, Loughry WJ. A "bloat-and-float" taphonomic model best explains the upside-down preservation of ankylosaurs. Palaeogeogr Palaeoclimatol Palaeoecol. 2018;497:117–27. https://doi.org/10.1016/j.palaeo.2018.02.010.

29. Schäfer W. Ecology and Palaeoecology of Marine Environments. Oertel I. transl. Chicago: Univ. Chicago Press; 1972.

30. Brand LR, Hussey M, Taylor J. Decay and disarticulation of small vertebrates in controlled experiments. J Taph. 2003;1:69–95.

31. Syme CE, Salisbury SW. Patterns of aquatic decay and disarticulation in juvenile Indo-Pacific crocodiles (*Crocodylus porosus*), and implications for the taphonomic interpretation of fossil crocodyliform material. Palaeogeogr, Palaeoclimatol, Palaeoecol. 2014;412:108–23. https://doi.org/10.1016/j.palaeo.2014.07.031.

32. Dodson P. Sedimentology and taphonomy of the Oldman formation (Campanian), Dinosaur Provincial Park, Alberta (Canada). Palaeogeogr Palaeoclimatol Palaeoecol. 1971;10:21–74.

33. Gangloff RA, Fiorillo AR. Taphonomy and paleoecology of a bonebed from the Prince Creek Formation, North Slope, Alaska. Palaios. 2010;25:299–317. https://doi.org/10.2110/palo.2009.p09-103r.

34. LeBlanc AR, Reisz RR, Evans DC, Bailleul AM. Ontogeny reveals function and evolution of the hadrosaurid dinosaur dental battery. BMC Evol Bio. 2016;16:152.

35. LeBlanc AR, Brink KS, Cullen TM, Reisz RR. Evolutionary implications of tooth attachment versus tooth implantation: A case study using dinosaur, crocodilian, and mammal teeth. J Vert Paleo. 2017;37:e1354006.

36. Conner CW. The Lance Formation: petrography and stratigraphy, Powder River basin and nearby basins, Wyoming and Montana. U.S. Geol Surv Bul. 1992;1917(II):1–17.

37. Weeks SR. Depositional model of a late Cretaceous dinosaur fossil concentration, Lance Formation. [thesis]. Loma Linda (CA): Loma Linda University; 2016.

38. Ulrich EO. Correlation by displacements of the strandline and the function and proper use of fossils in correlation: Bul Geol Soc Amer. 1916;27:451–90.

39. Brett CE, Goodman WM, LoDuca ST. Sequences, cycles, and basin dynamics in the Silurian of the Appalachian Foreland Basin. Sed Geol. 1990;69:191–244.

40. Allen P. Strand-line pebbles in the mid-Hastings Beds and the geology of the London Uplands, Old Red Sandstone, New Red Sandstone and other pebbles. Conclusion. Proc Geol Assoc. 1967;78:241–76.

41. Malenda H, Kutztown P., Simpson E, Reading PA, Fillmore D. Depositional setting of fish-part sandstones and conglomerates: A taphonomic interpretation of a rare lacustrine strand line. Soc Vert Paleo. 2010;30:128A.

42. Binns RE. Composition and derivation of pumice on postglacial strandlines in northern Europe and the western Arctic. Geol Soc Amer Bul. 1972;83:2303–24.

43. Benton MJ. Ichnology: Sedimentological use of dinosaurs. Nature. 1986;321:732.

44. Lockley MG. The paleobiological and paleoenvironmental importance of dinosaur footprints. Palaios. 1986;1:37–47.

45. Leggitt VL, Buchheim HP. Bird bone taphonomic data from recent lake margin strandlines compared with an Eocene *Presbyornis* (Aves: Anseriformes) bone strandline. Geol Soc Amer Abstr. 1997;29:A105.

46. Rogers RR, Kidwell SM. A conceptual framework for the genesis and analysis of vertebrate skeletal concentrations. Introduction. In: Rogers R R, Eberth D A, Fiorillo AR editors. Bonebeds: Genesis, Analysis and Paleobiological Significance. Chicago: Univ of Chicago Press; 2007. p. 1–63.

47. Strganac C, Jacobs LL, Polcyn MJ, Mateus O, Myers TS, Salminen J, May SR, Araújo R, Ferguson KM, Gonçalves AO, Morais ML. Geological setting and paleoecology of the Upper Cretaceous Bench 19 marine vertebrate bonebed at Bentiaba, Angola. Netherlands J Geosci. 2015;94:121–36. https://doi.org/10.1017/njg.2014.32.

48. Rogers RR, Arcucci AB, Abdala F, Sereno PC, Forster CA, May CL. Paleoenvironment and taphonomy of the Chañares Formation tetrapod assemblage (Middle Triassic), northwestern Argentina: Spectacular preservation in volcanogenic concretions. Palaios. 2001;16:461–81.

49. Varricchio DJ. Jack's birthday site, a diverse dinosaur bonebed from the Cretaceous Two Medicine Formation of Montana. [dissertation]. Bozeman (MT): Montana State University; 1995.

50. Tasch P, Zimmerman JR. Fossil and living Conchostracan distribution in Kansas-Oklahoma across a 200-Millon-Year Time Gap. Science. 1961;133:584–586.

51. Janensch W. Bericht iiber den Verlauf der Tendaguru-Expedition. Archiv fiir Biontologie. 1914;3:17–58. German.

52. Russell D, Béland P, Mcintosh JS. Paleoecology of the dinosaurs of Tendaguru (Tanzania). Mémoires de La Société Géologique de France (Nouvelle Série). 1980;139:169–75.

53. Heinrich WD. The taphonomy of dinosaurs from the Upper Jurassic of Tendaguru (Tanzania) based on field sketches of the German Tendaguru expedition (1909–1913). Fossil Record. 1999;2:25–61.

54. Weigelt J. Recent vertebrate carcasses and their paleobiological implications. Schaefer J translator: Chicago; Univ of Chicago Press; 1989.

Figure 1

Click here to access/download;Figure;Figure 1 Lance Formation.tif

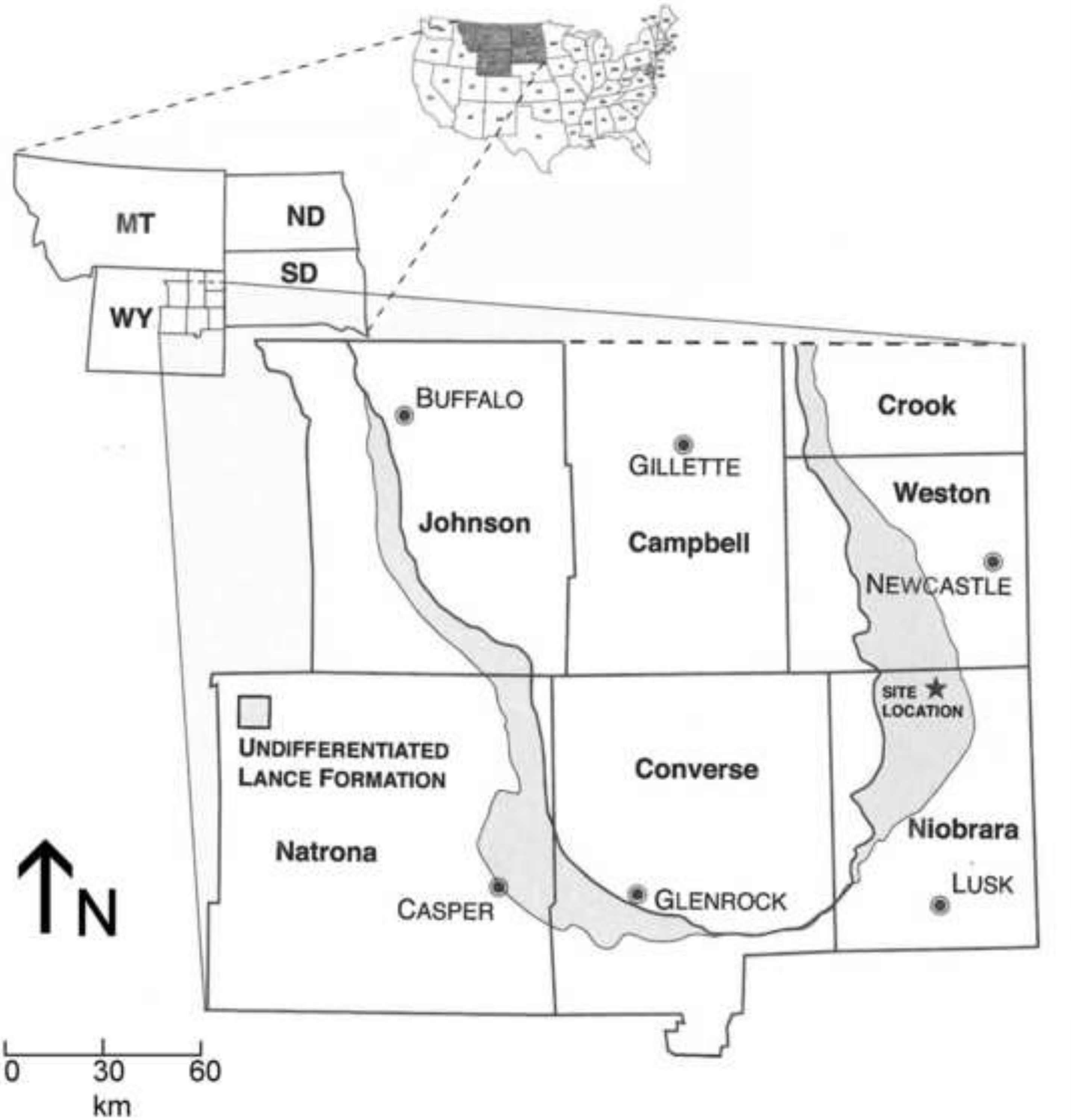

Figure 2

Click here to access/download;Figure;Figure 2 Line of quarries.tif

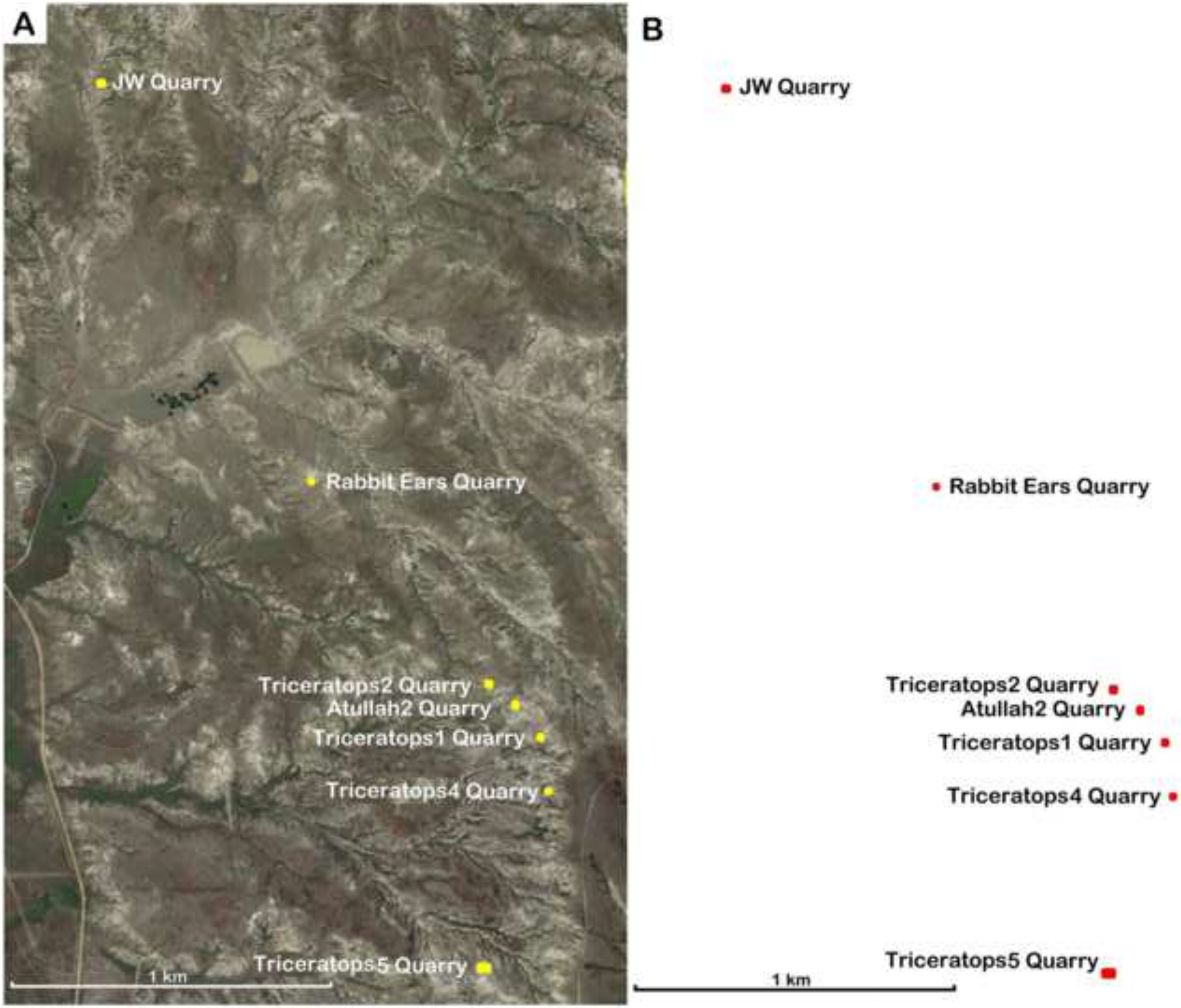

Figure 3

Click here to access/download;Figure;Figure 3 Paleo map.tif

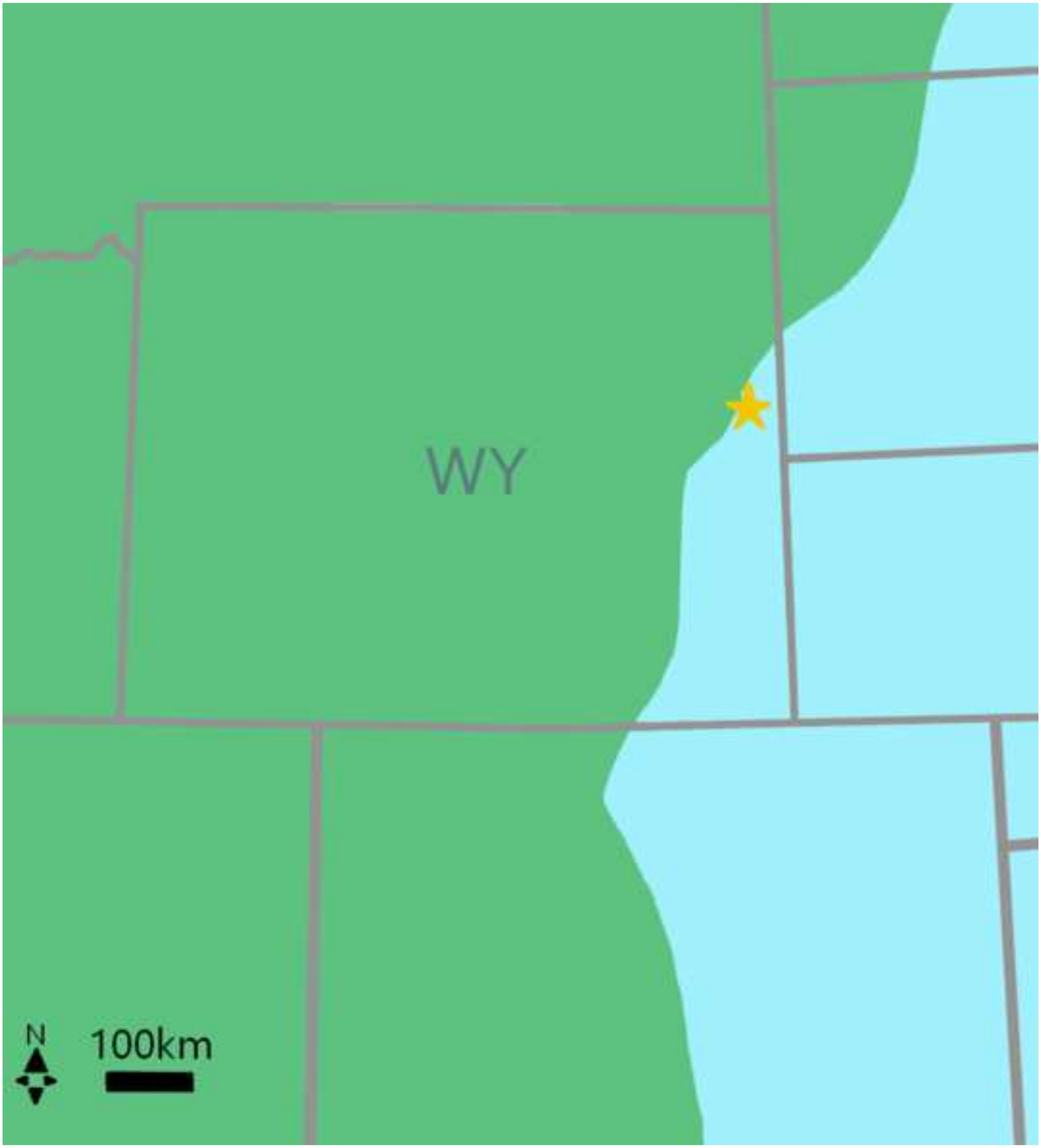

Figure 4

Click here to access/download;Figure;Figure 4 All Tri2 bones - colors.tif

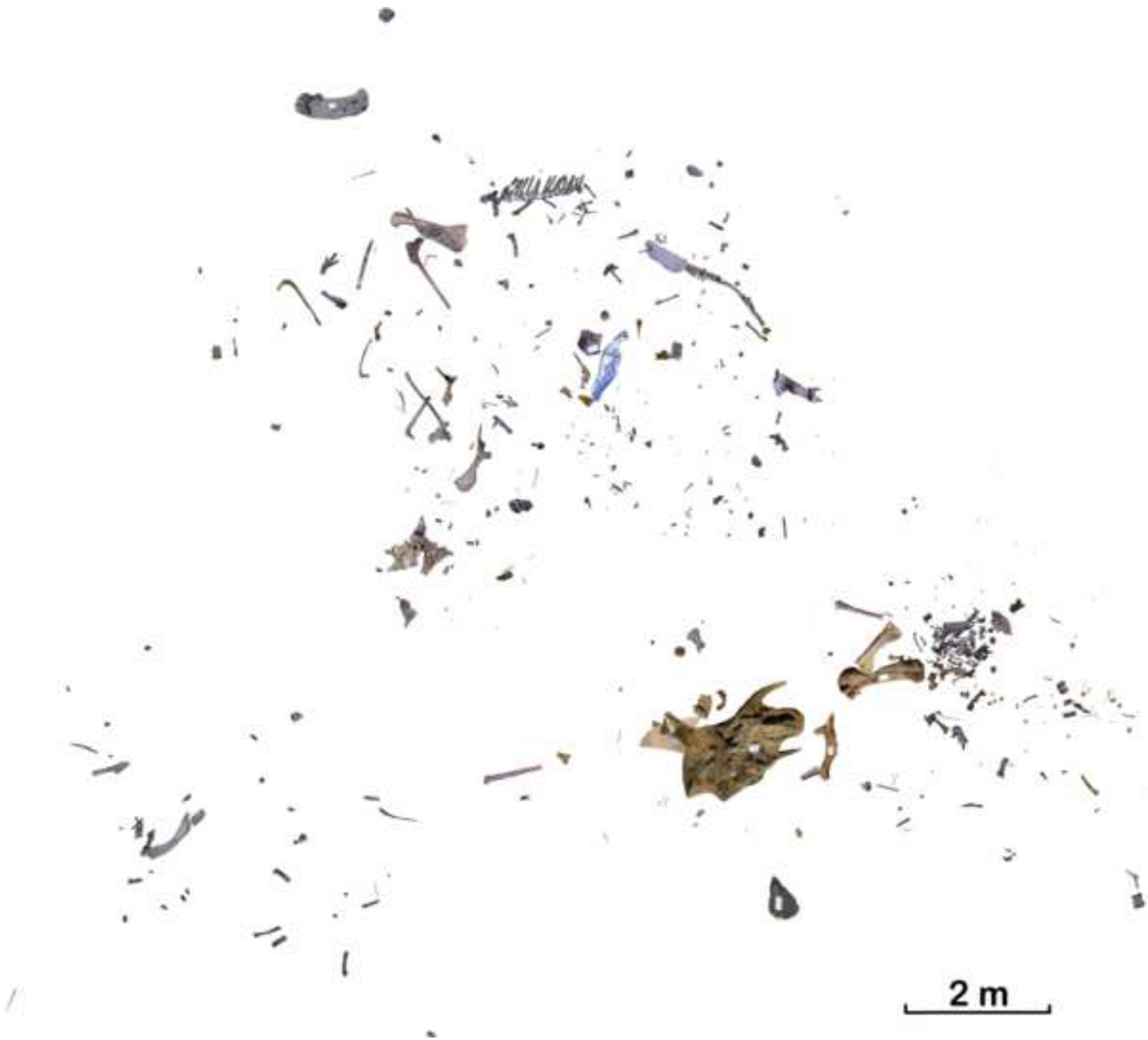

Figure 5 Click here to access/download;Figure;Figure 5 All rose diagrams.tif

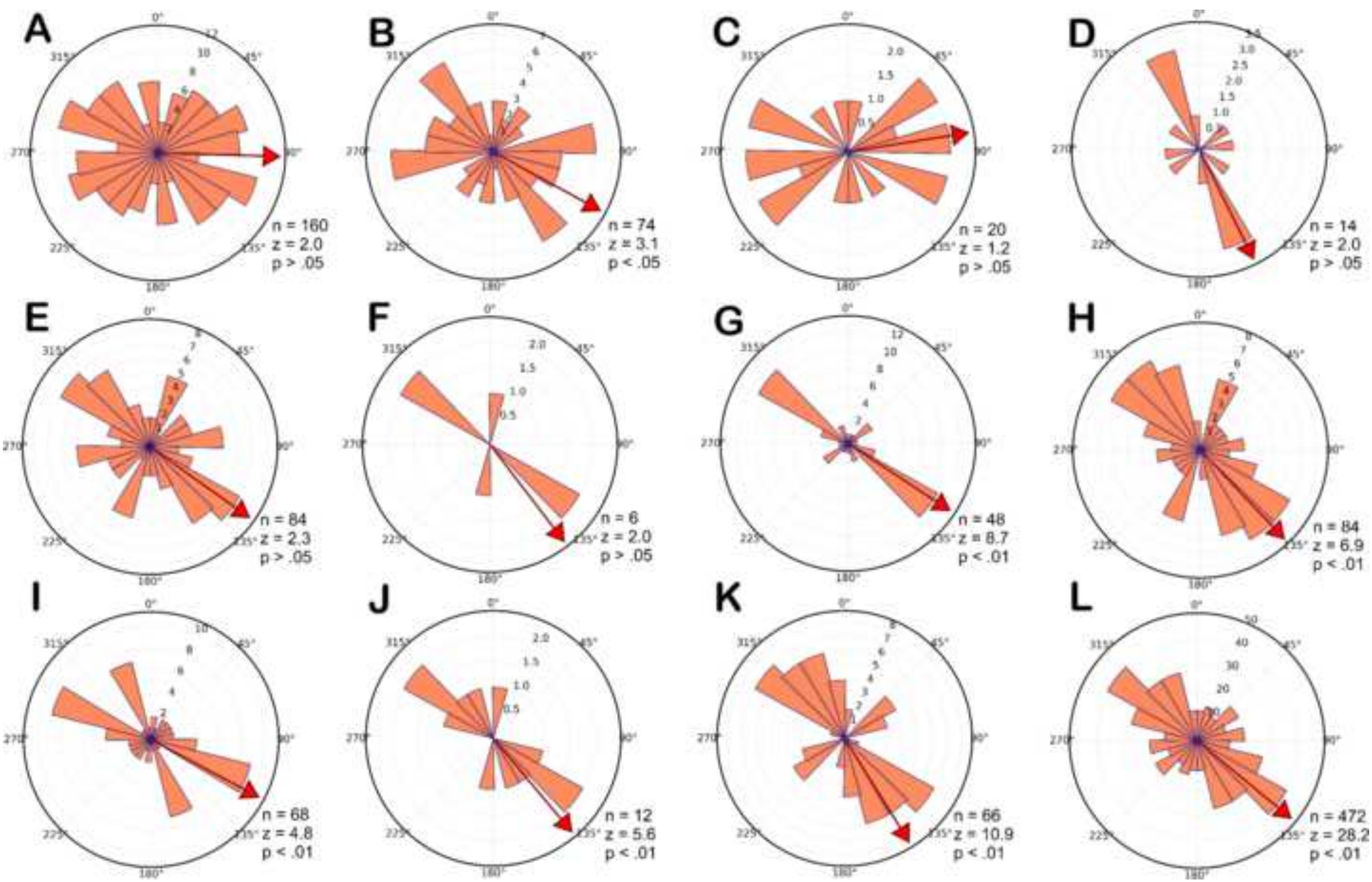

Figure 6

Click here to access/download;Figure;Figure 6 Tri2 Tricer bones.tif

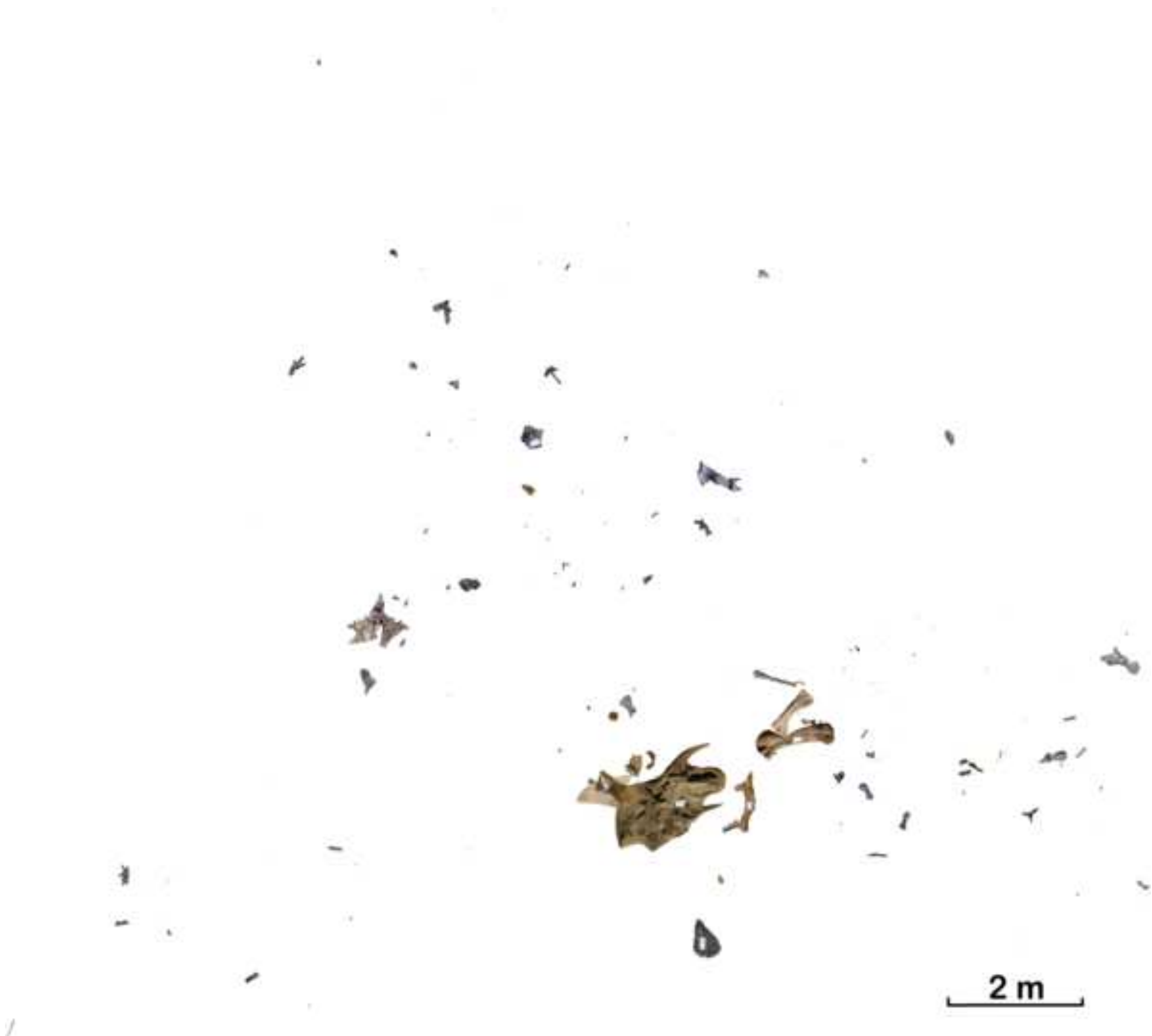

Figure 7

Click here to access/download;Figure;Figure 7 Tricer. body.tif

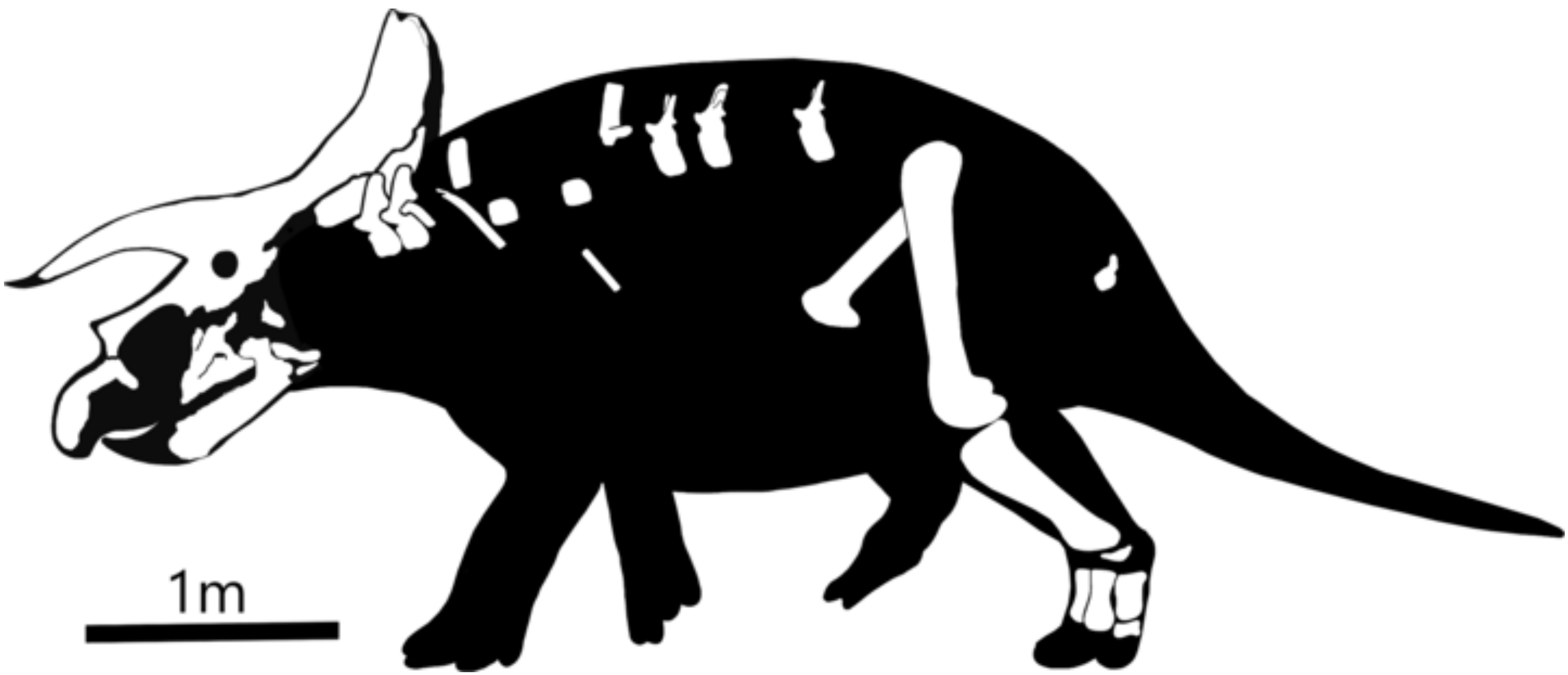

Figure 8

Click here to access/download;Figure;Figure 8 Tri2 Anzu bones.tif

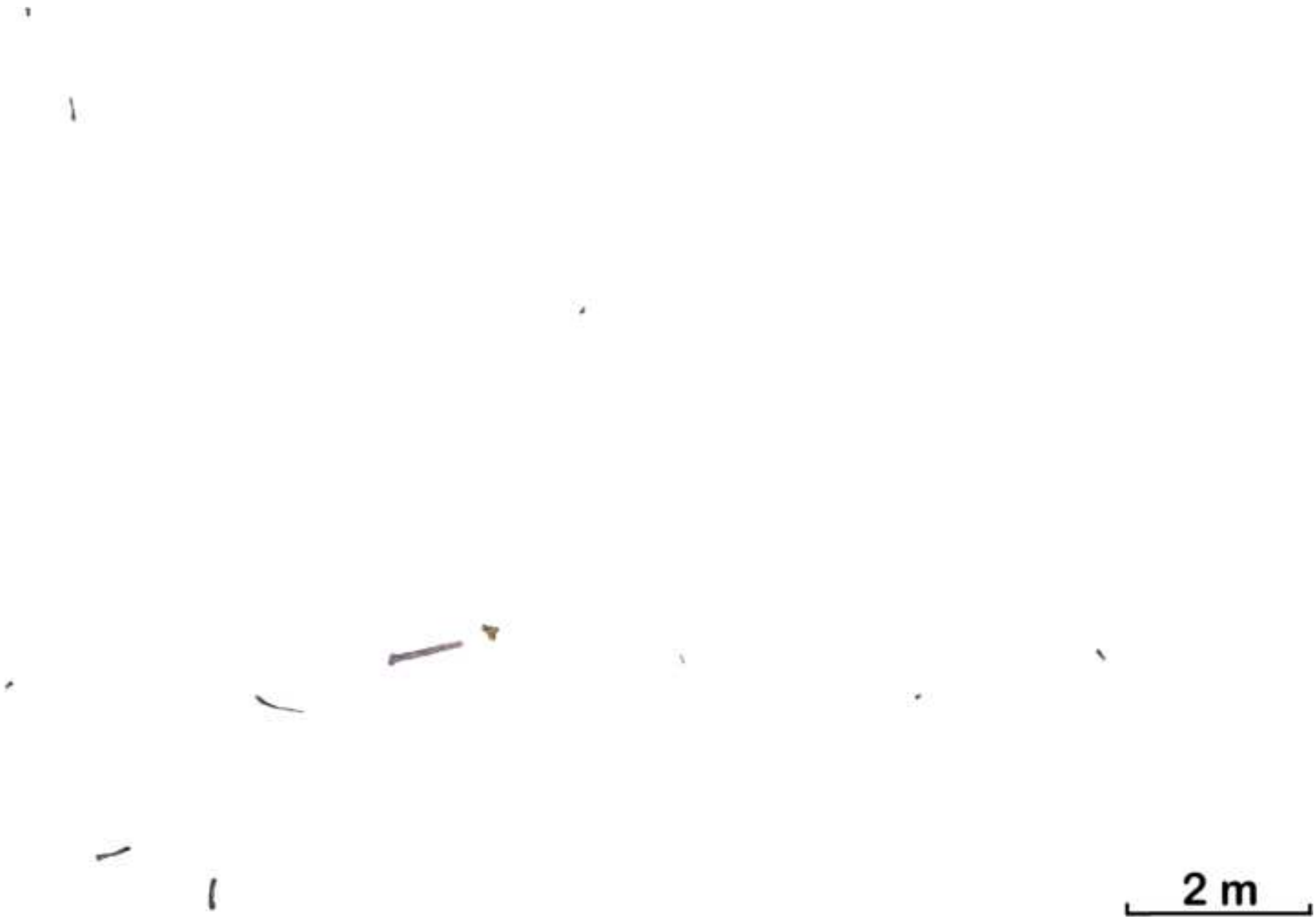

Figure 9

Click here to access/download;Figure;Figure 9 Tri2 Anzu body.tif

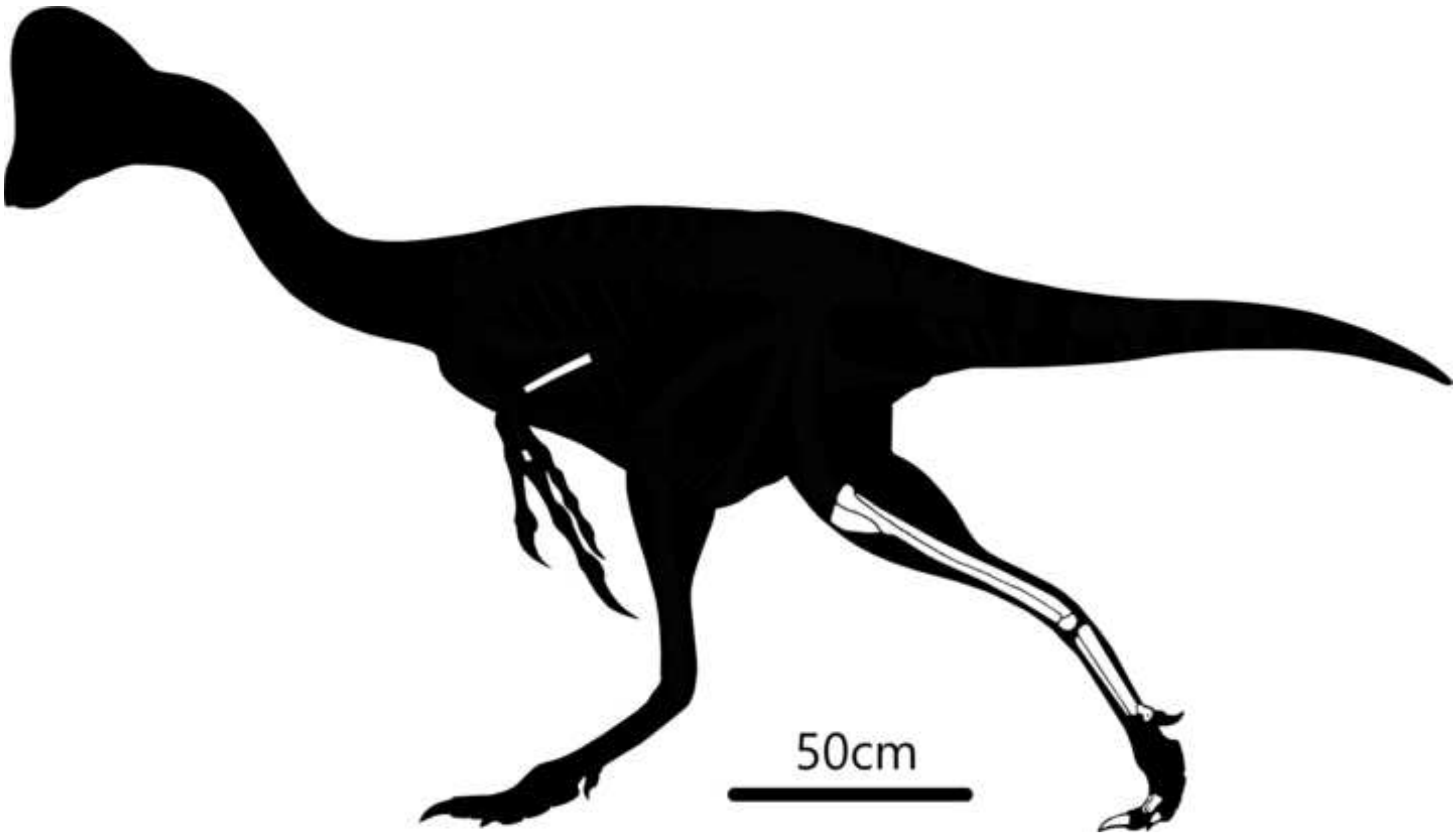

Figure 10

Click here to access/download;Figure;Figure 10 Tri2 Thesc. bones.tif

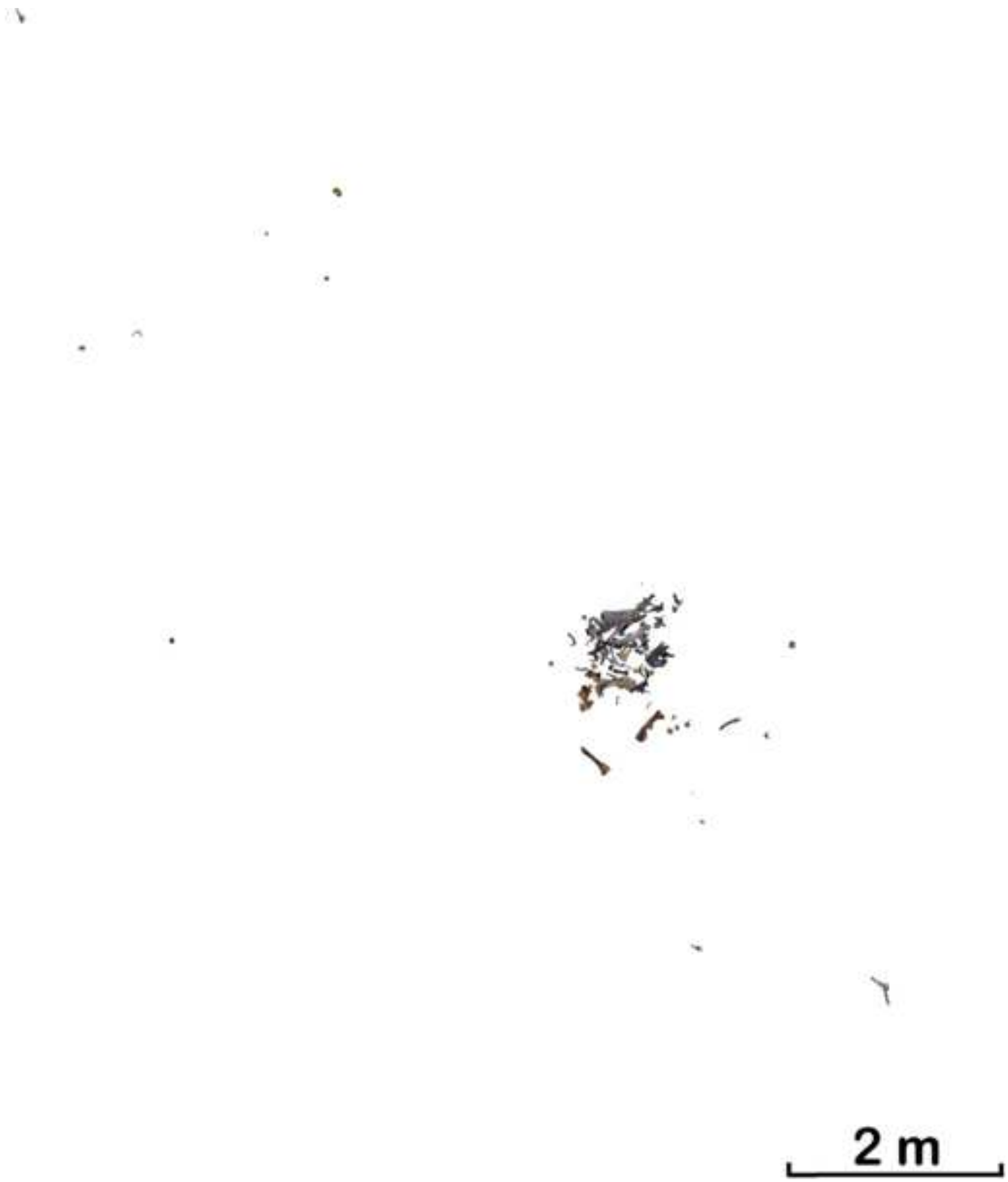

Figure 11

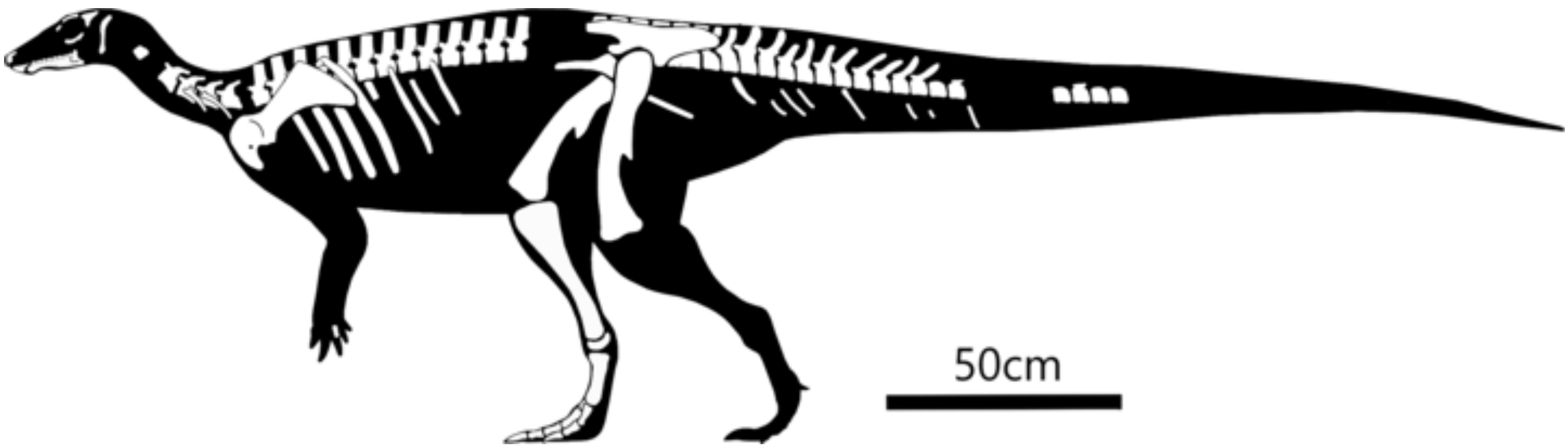

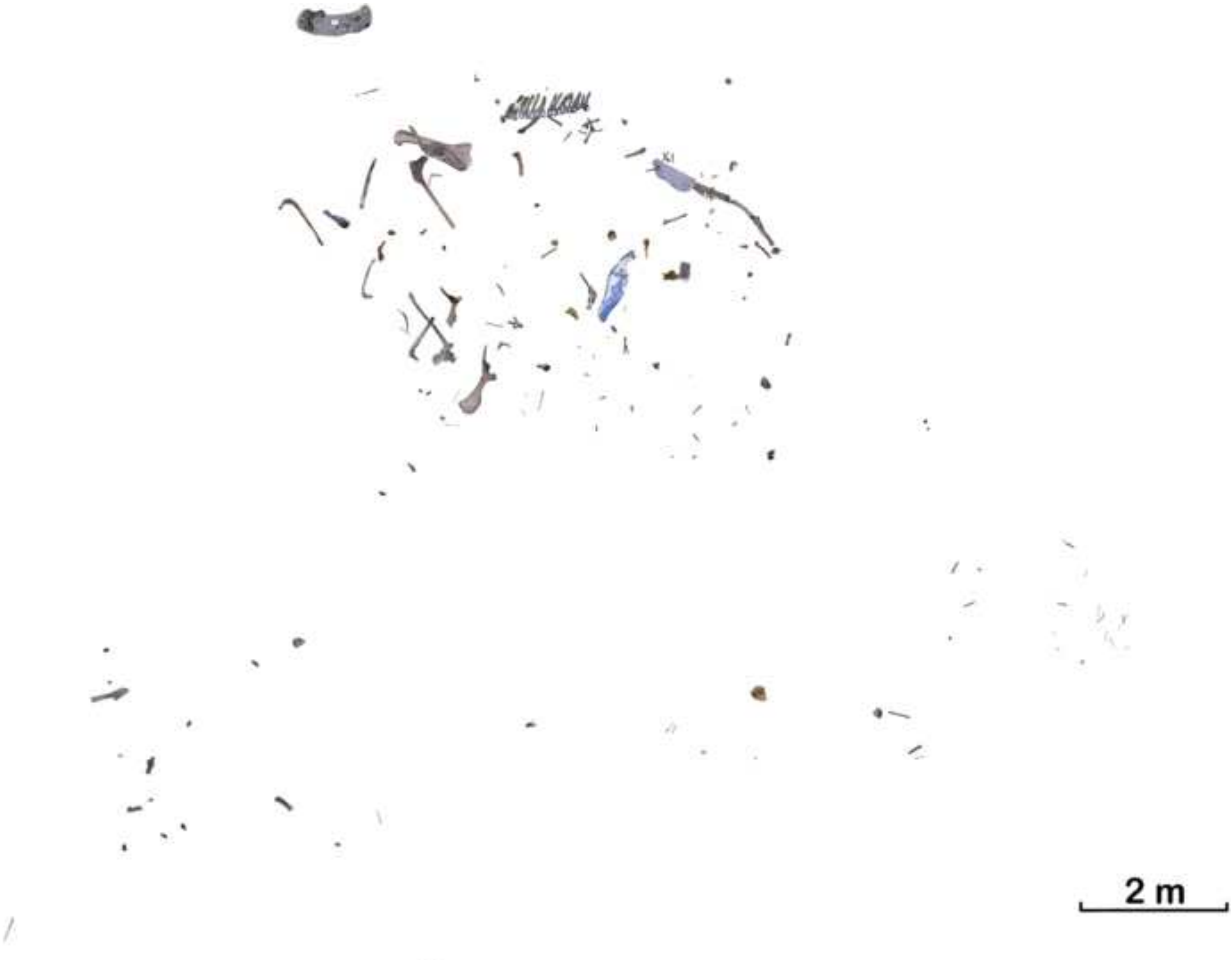
2 m

Figure 13

Click here to access/download;Figure;Figure 13 Tri2 Edmont. body.tif

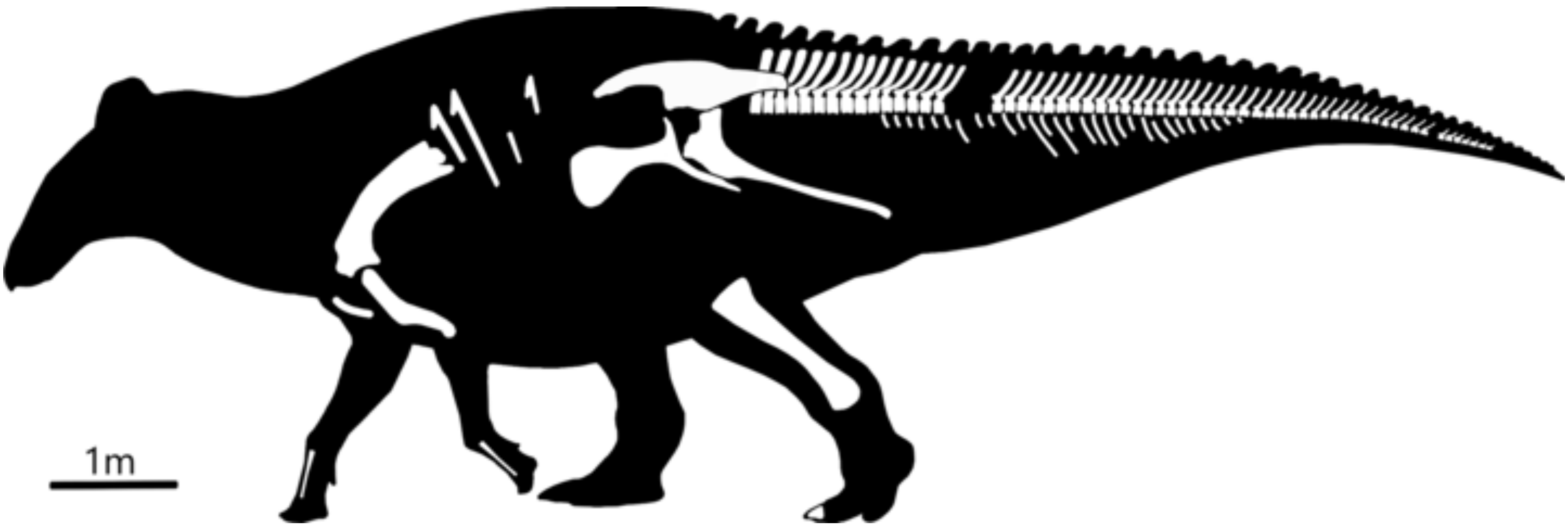

Figure 14

Click here to access/download;Figure;Figure 14 Tri2 testudines bones.tif

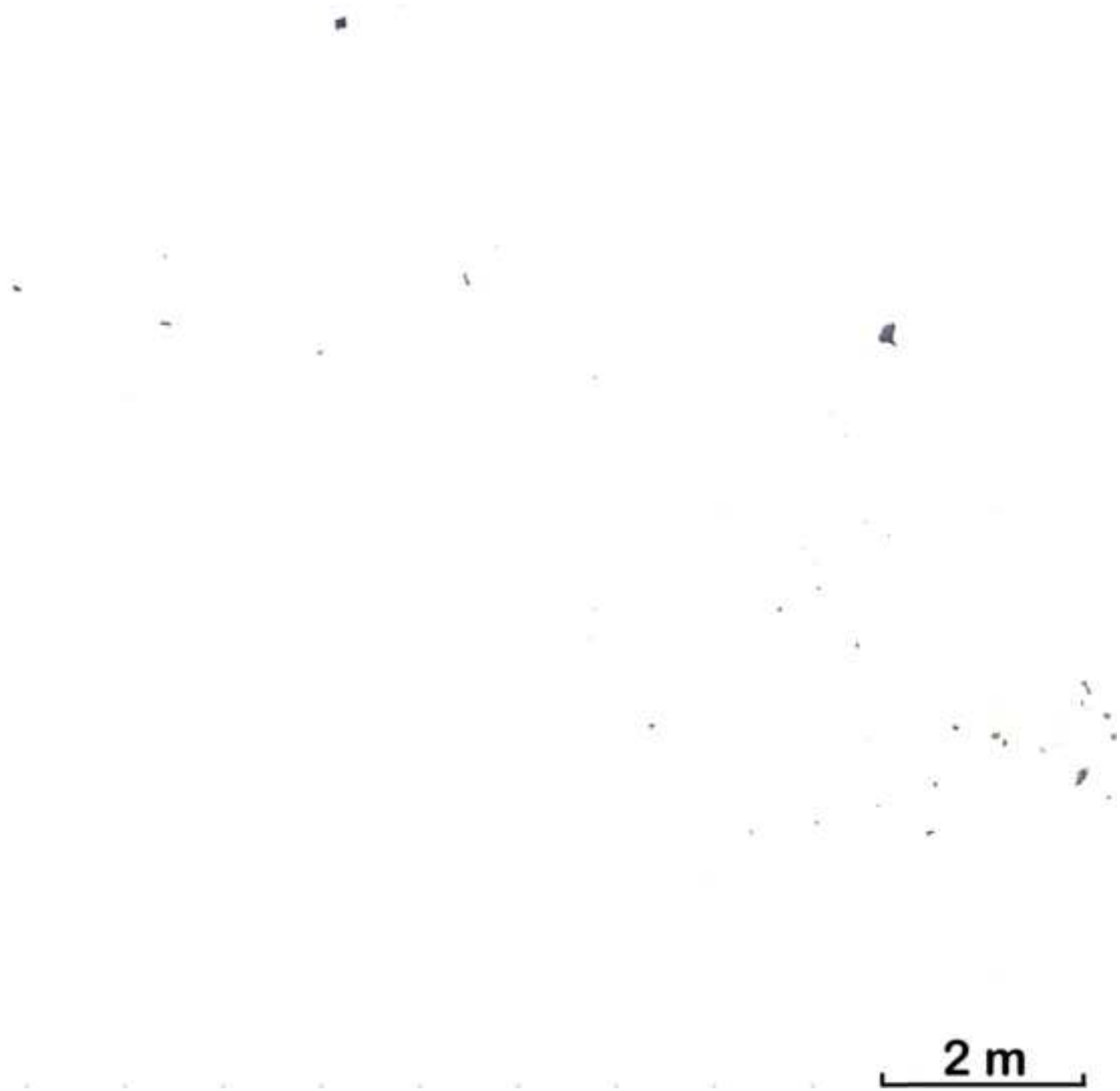

Figure 15

Click here to access/download;Figure;Figure 15 Tri1 bones.tif

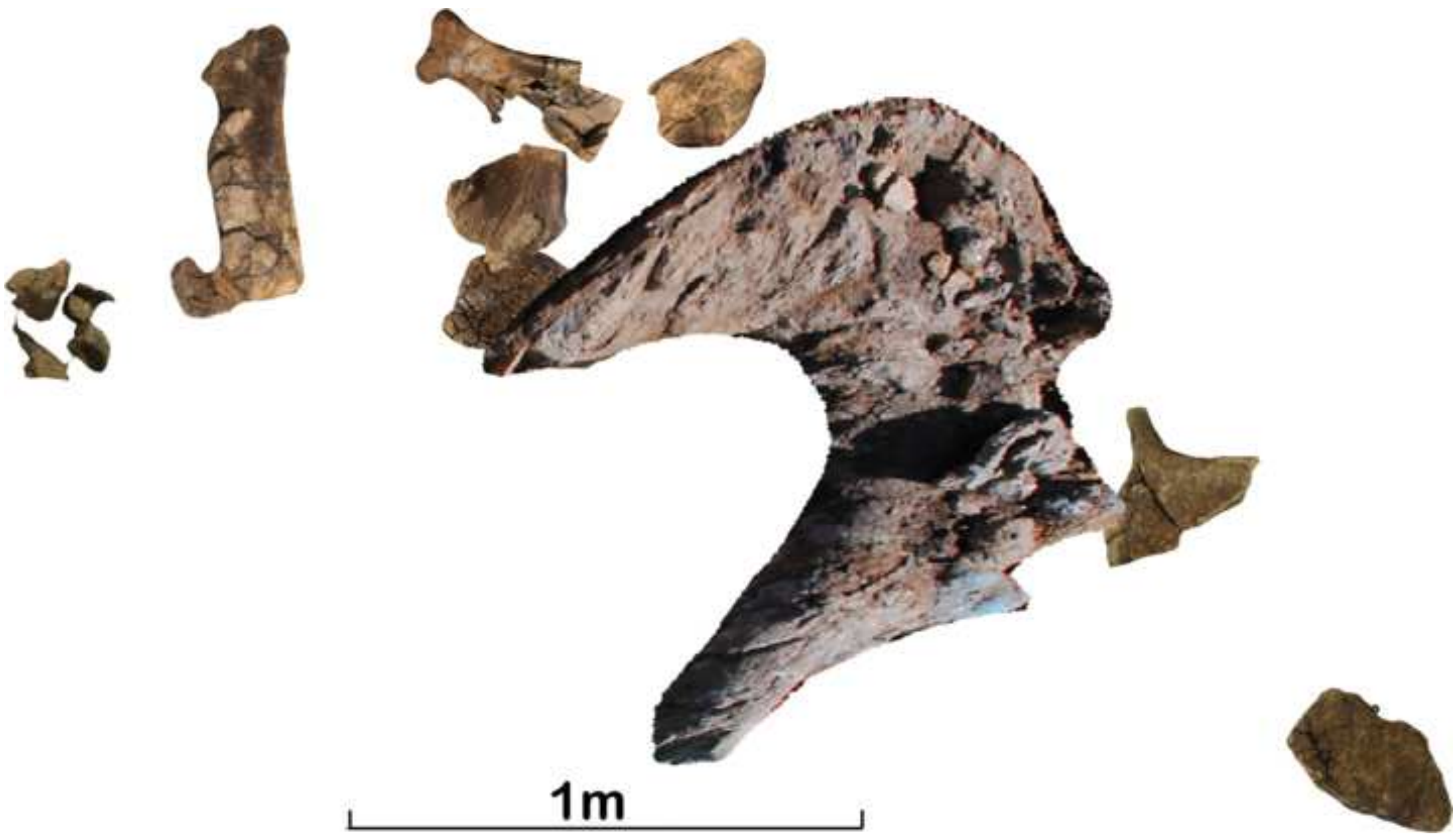

Figure 16

Click here to access/download;Figure;Figure 16 Tri1 body.tif

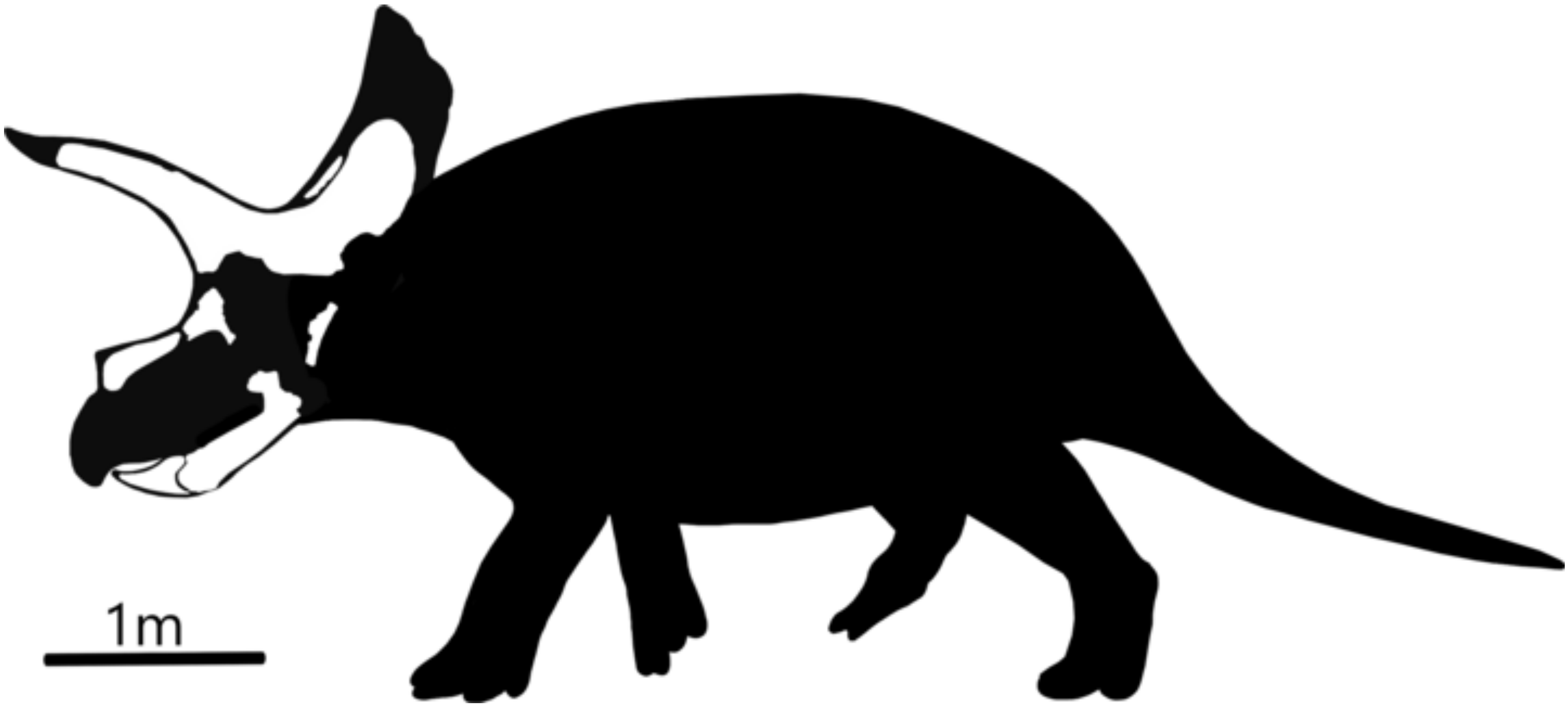

Figure 17

Click here to access/download;Figure;Figure 17 Atullah2 bones.tif

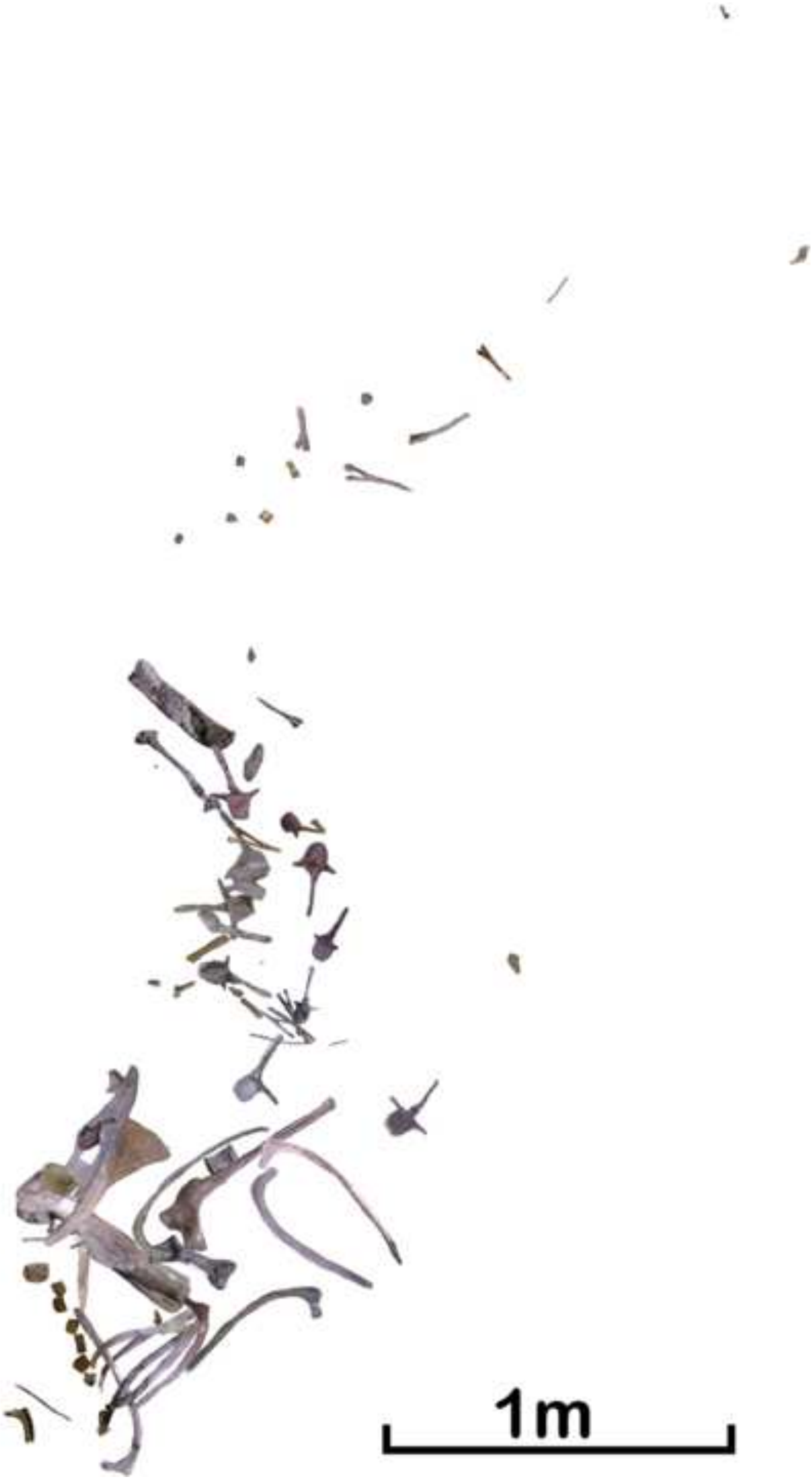

Figure 18

Click here to access/download;Figure;Figure 18 Atullah2 body.tif

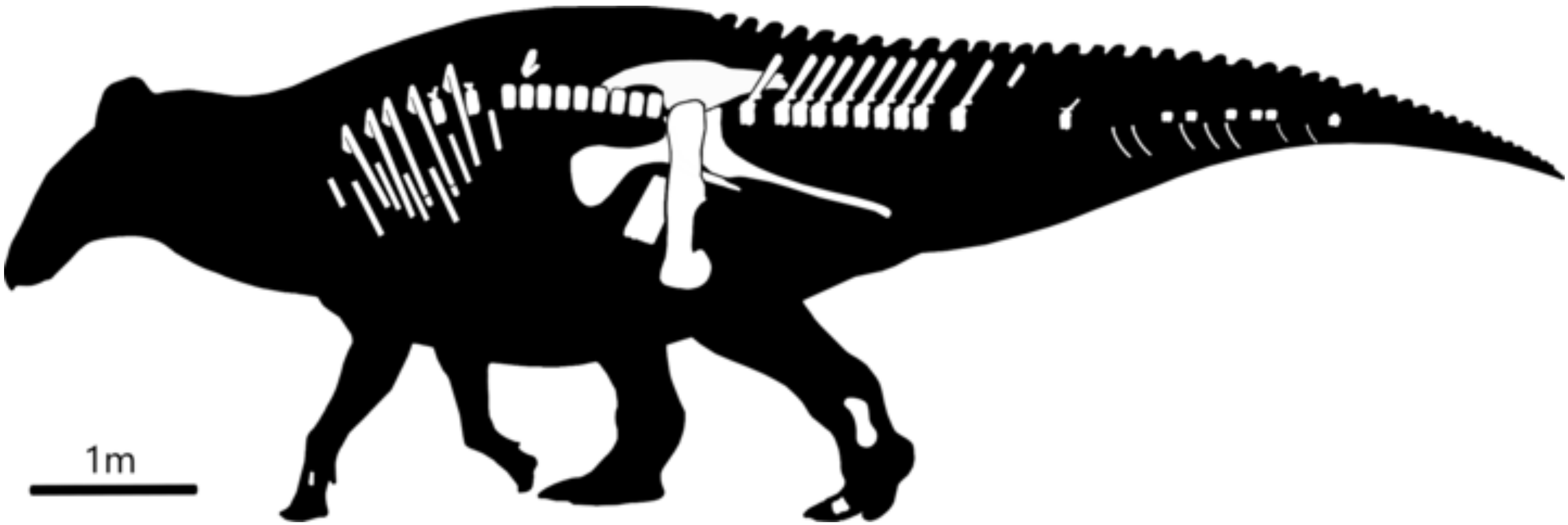

Figure 19

Click here to access/download;Figure;Figure 19 JW bones.tif

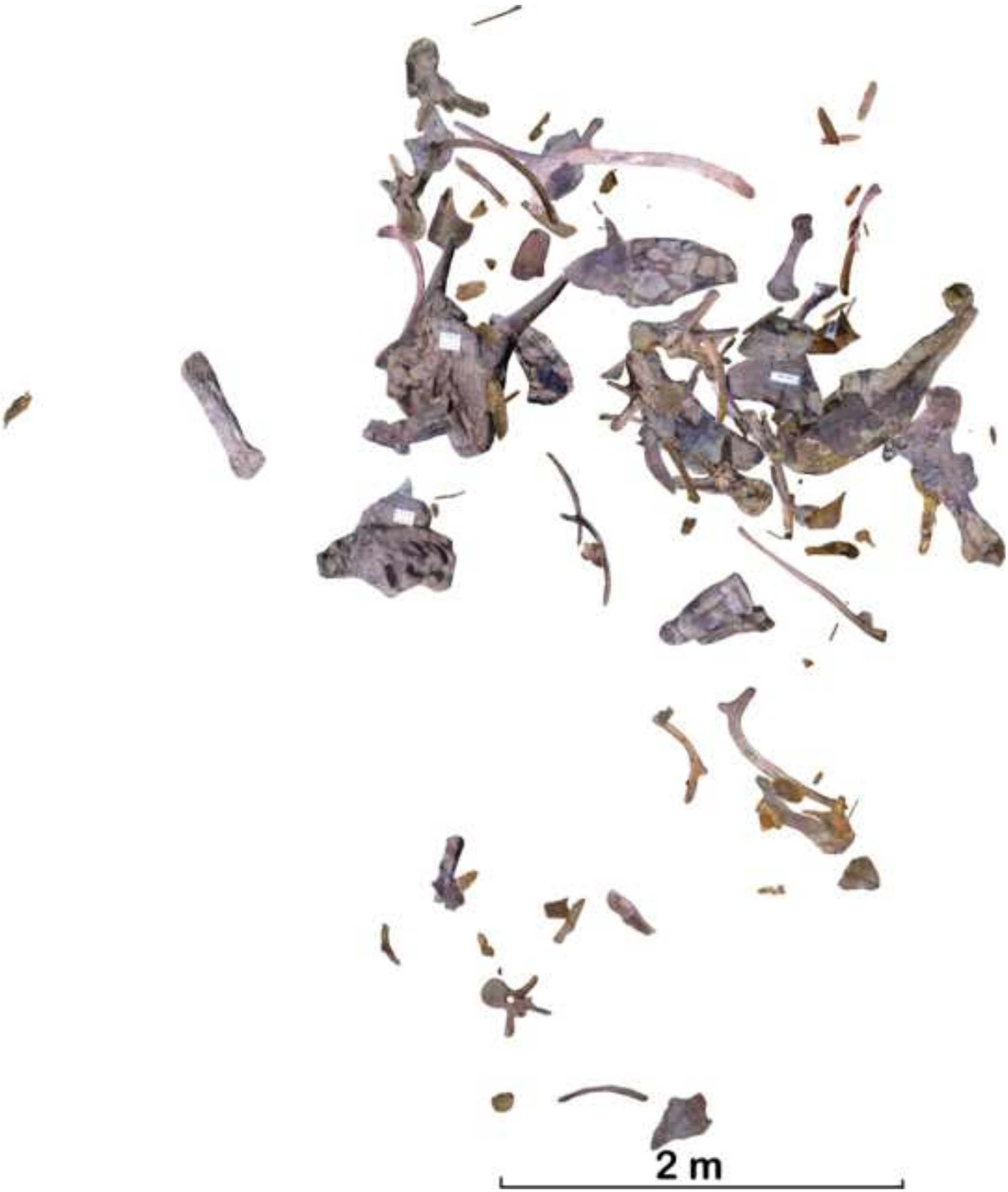

Figure 20

Click here to access/download;Figure;Figure 20 JW body.tif

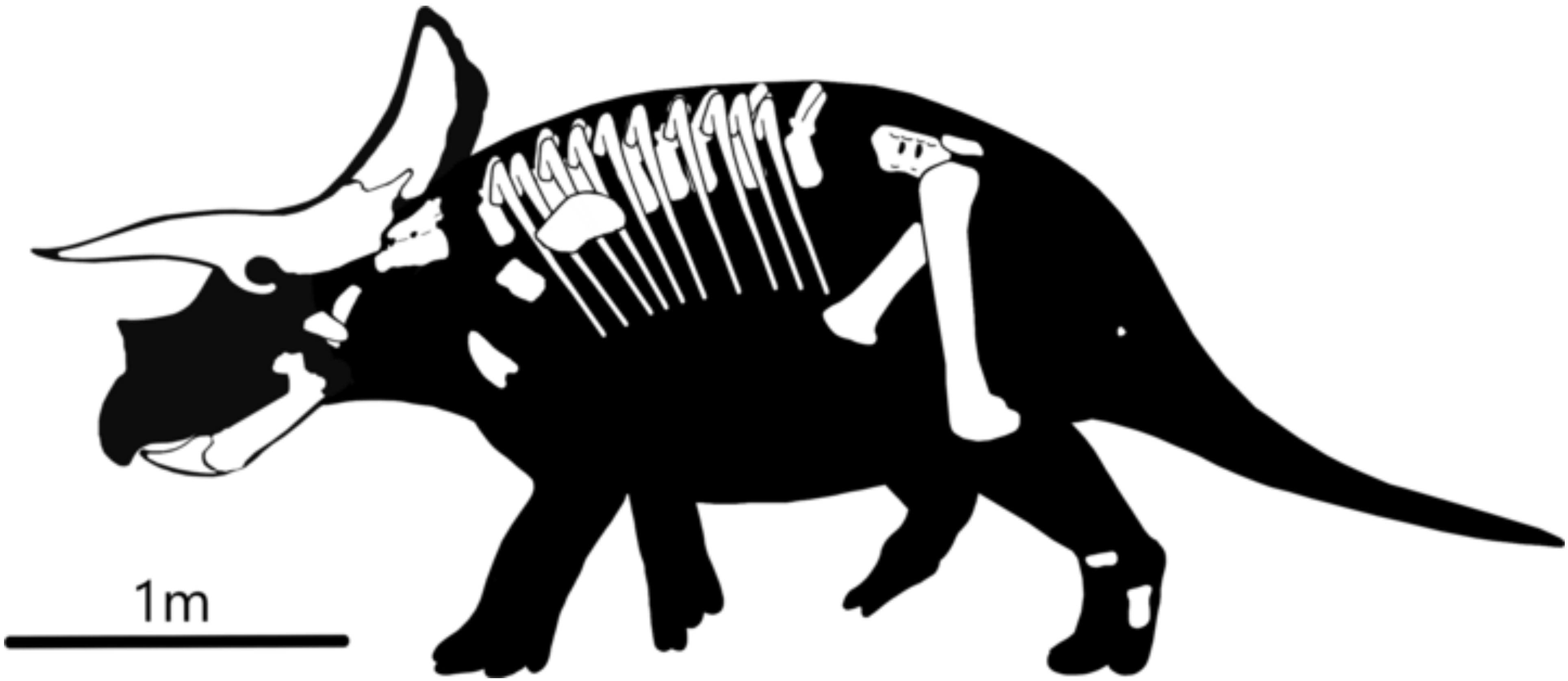

Figure 21

Click here to access/download;Figure;Figure 21 Tri4 bones.tif

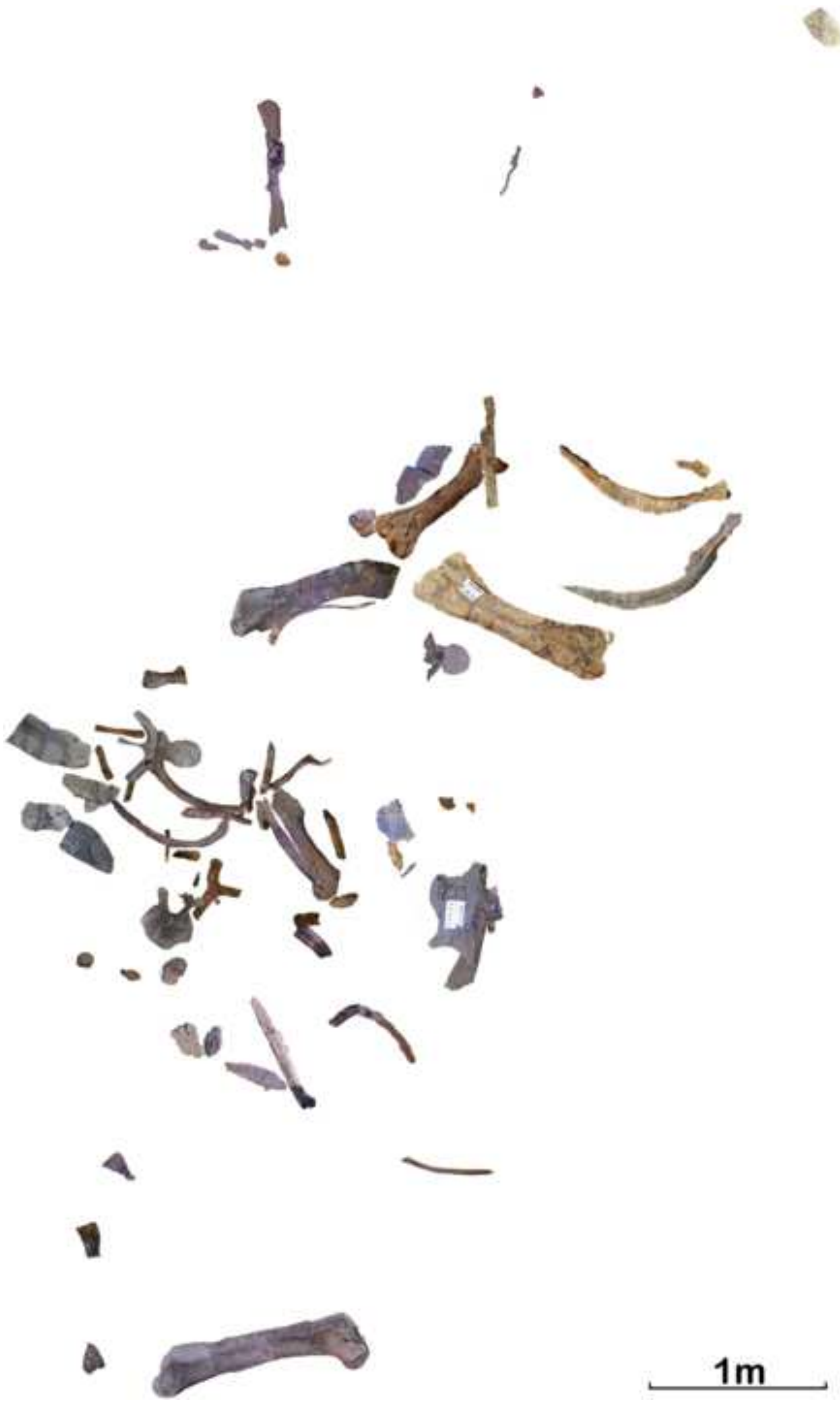

Figure 22

Click here to access/download;Figure;Figure 22 Tri4 body.tif

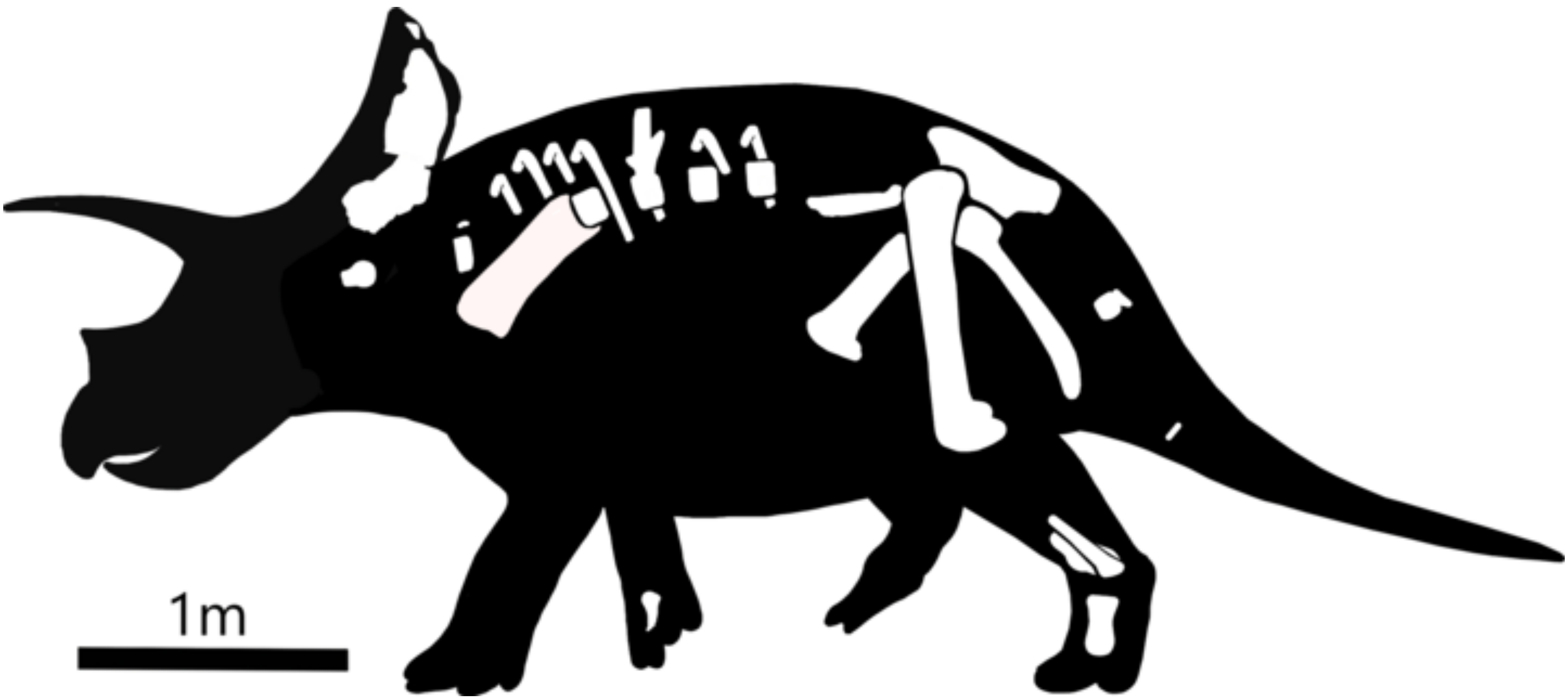

Figure 23

Click here to access/download;Figure;Figure 23 Rabbit Ears bones.tif

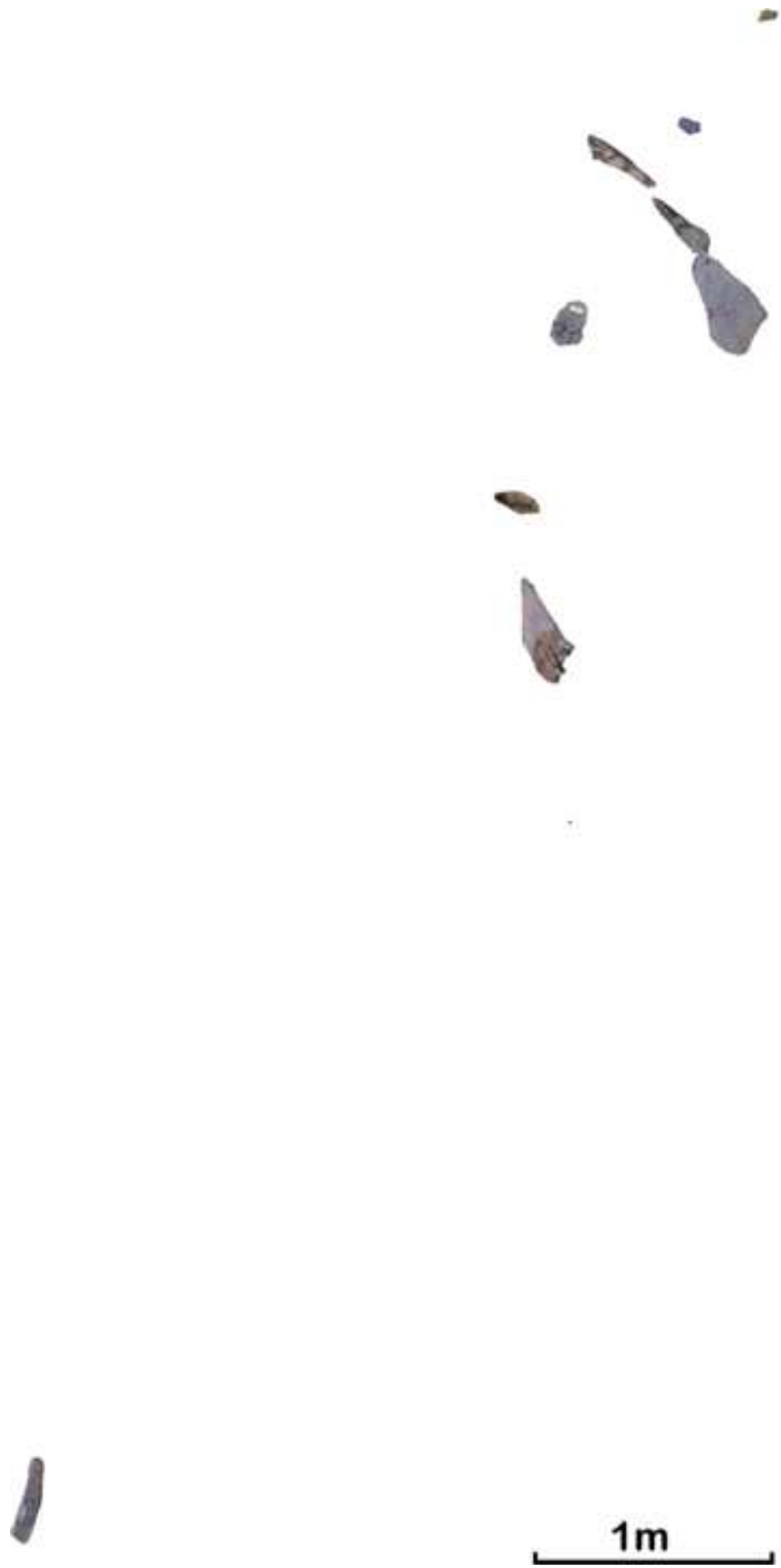

Figure 24 Click here to access/download;Figure;Figure 24 Tri5 bones.tif

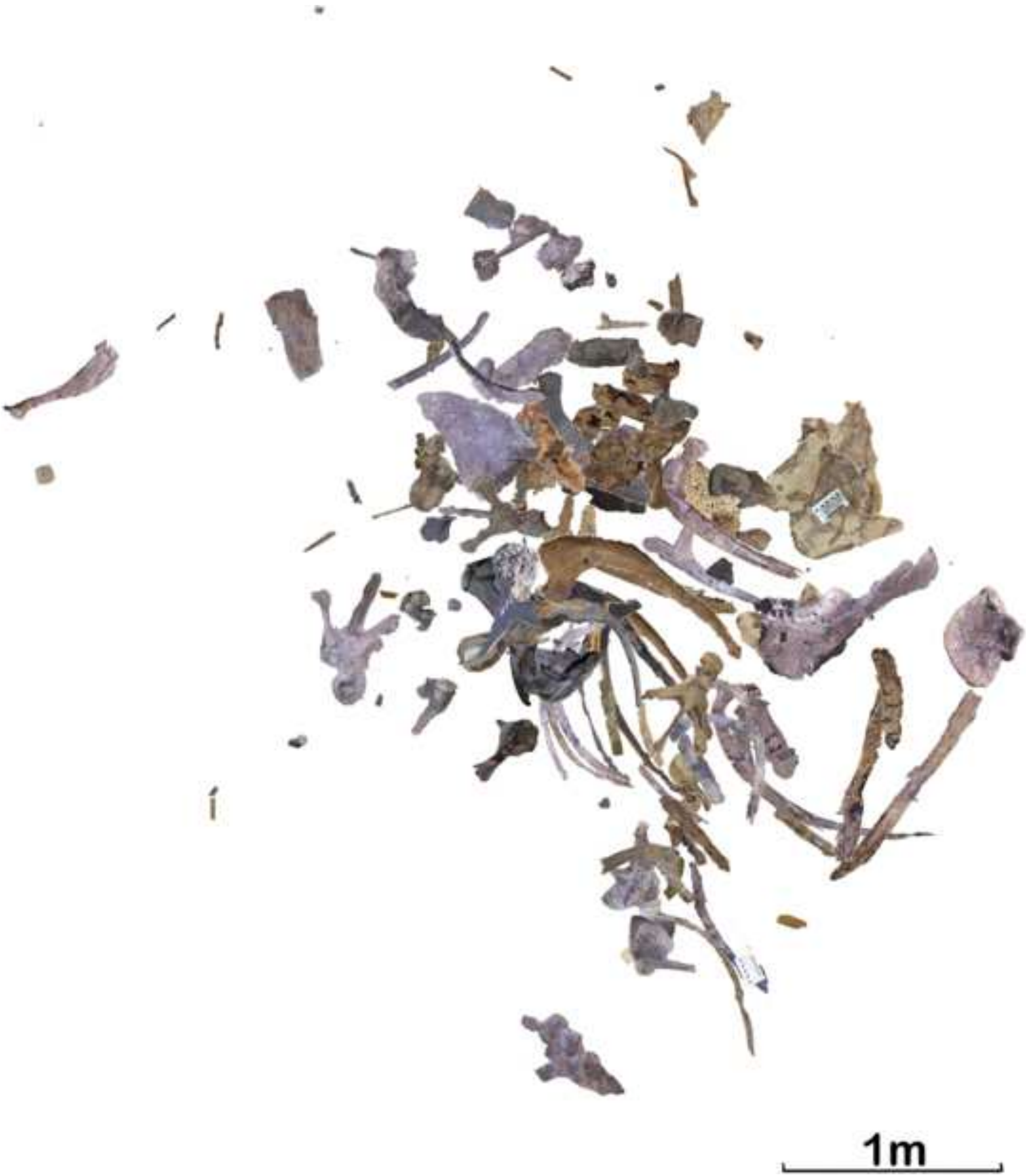

Figure 25

Click here to access/download;Figure;Figure 25 Tri5 body.tif

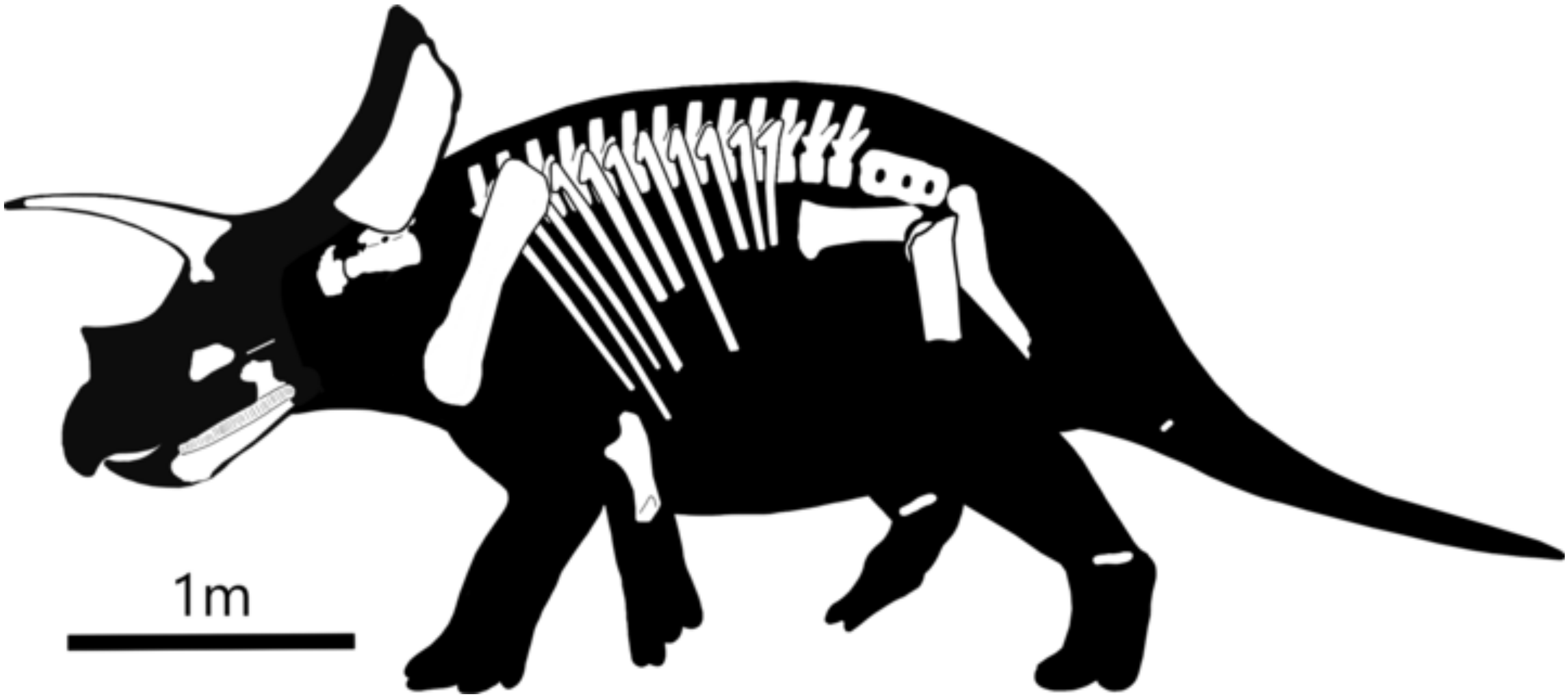

Figure 26

Click here to access/download;Figure;Figure 26 Stratigraphic sections.tif

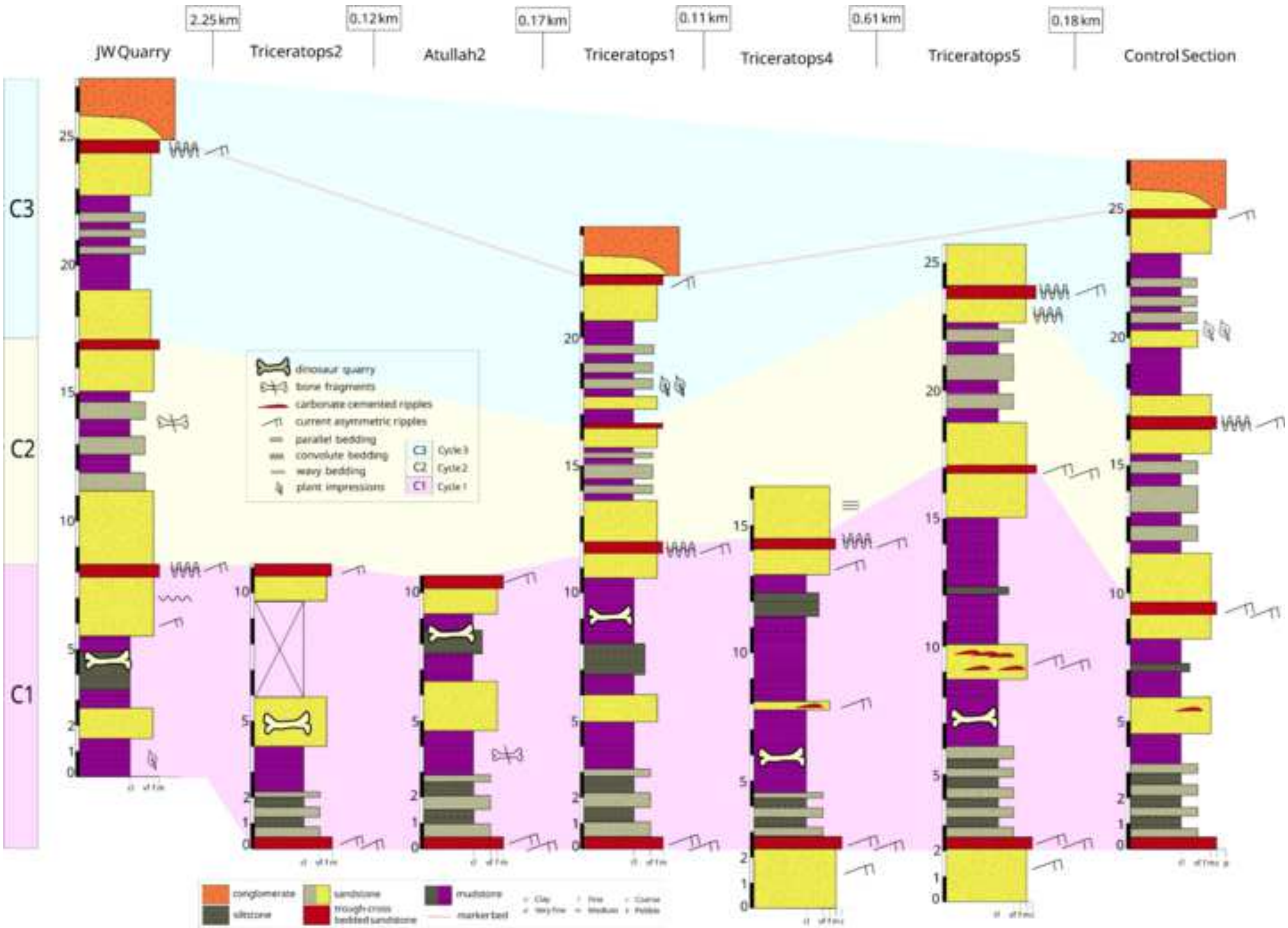

Supplement: Supplementary file 1 [file pone.0362687.s002.pdf]
