# Supplementary material for: Evidence for an upper cretaceous strandline deposit in the Lance Formation, Wyoming, USA
Source: PLoS One. 2026 Nov 12;21(11):e0362687. doi: 10.1371/journal.pone.0362687 (PMC13759098; doi:10.1371/journal.pone.0362687)
Supplement: Supplementary file 2 [file pone.0362687.s001.docx]

**General Comments**

1. The language throughout the manuscript requires significant revision to adhere to the conventions of formal scientific writing. The authors should adopt a more objective, professional tone, minimizing the use of first-person pronouns (e.g., "we," "our") in favor of the passive voice where appropriate. This shift will ensure the focus remains on the methodology and the data rather than the researchers.
2. The overall structure of the manuscript must be improved to establish a coherent and logical flow. Reorganizing the narrative to better guide the reader systematically through the geological context, taphonomic data, and subsequent paleoenvironmental interpretations will greatly enhance the manuscript's comprehensibility.
3. The visual presentation of the data needs substantial streamlining. The current number of figures is excessive and disrupts the flow of the text. The authors must reduce the total figure count by combining related images into structured, multi-panel composite figures (for example, aggregating the individual quarry maps and skeletal diagrams into unified plates). Furthermore, the overall resolution, labeling, and quality of the figures must be upgraded to meet publication standards.

**Major Critical Concerns**

1. The authors anchor their interpretation of a marginal marine environment for Cycle 1 entirely on the visual presence of "glauconite grains and glauconitic cement" derived from basic petrographic analysis. Relying solely on visual petrography to characterize these components is insufficient. To properly evaluate the diagenetic overprinting and the origin of these minerals, the study must apply standard methodologies, including Q-XRD (Quantitative X-ray Diffraction) and scanning electron microscopy (SEM) paired with detailed thin-section microscopy. Without these techniques, it is impossible to definitively prove whether the glauconite is authigenic (forming *in situ* and proving marine influence) or reworked/detrital.
2. The use of double-angle transformation for the bidirectional long-bone data is standard, but the resolution of the 180-degree ambiguity is highly problematic. The authors admit to resolving this ambiguity by using a "known paleocurrent direction, inferred from independent sedimentary structures" to assume a southeast flow. This borders on circular reasoning. If independent sedimentary structures (such as the briefly mentioned trough crossbedding or current ripples) establish the flow, those specific data points must be quantified and presented in their own rose diagrams. The authors cannot use an undocumented assumption to force bidirectional bone data into a unidirectional conclusion.
3. Connecting seven distinct quarries across a 2.5 km transect without continuous outcrop exposure is a massive interpretive leap. The authors note that continuous stratigraphic tracing is "difficult due to deep weathering, poor outcrop exposure, and limited resolution". Given this lack of continuous control, aligning the sites along a ~140-degree azimuth and concluding they represent a single, contiguous strandline event is highly speculative. The manuscript fails to rule out the possibility that these are simply localized fluvial channel lags or point-bar deposits that happen to align spatially within the broader basin architecture.
4. The "bloat and float" model is invoked to explain the missing skulls and cervical vertebrae. However, the manuscript downplays the extreme variance in preservation within Triceratops2 Quarry itself, which contains a tightly associated *Thescelosaurus* alongside highly disarticulated *Anzu* elements. If these carcasses were floating in a marine setting and stranded simultaneously, the authors need to rigorously explain the biomechanical or temporal reasons for such vastly different states of decay upon deposition.

These required revisions are substantial and are intended to elevate the analytical rigor of the manuscript. The dataset compiled from these Lance Formation quarries clearly represents a massive undertaking and a valuable contribution to Maastrichtian taphonomic studies. By integrating standard analytical methodologies and firmly grounding the stratigraphic correlations, the resulting paleoenvironmental interpretations will be significantly stronger and more impactful for the broader paleoecological community. I suggest authors resubmit the manuscript after addressing the suggested concerns.
