# Supplementary material for: Evidence for an upper cretaceous strandline deposit in the Lance Formation, Wyoming, USA
Source: PLoS One. 2026 Nov 12;21(11):e0362687. doi: 10.1371/journal.pone.0362687 (PMC13759098; doi:10.1371/journal.pone.0362687)
Supplement: Supplementary file 3 [file pone.0362687.s003.docx]

Dear Prof. Santanu Banerjee,

Thank you very much for inviting me to review the manuscript by Dr Keith Snyder et al. submitted to the PLOS One journal.

Manuscript entitled "Identification of an Upper Cretaceous strandline in the Lance Formation, Wyoming, USA" (PONE-D-26-22387).

I recommend the manuscript for publication, subject to minor revisions. Several corrections have been highlighted in yellow within the PDF manuscript, and additional comments and suggestions have been provided in the accompanying Word document. Once these minor issues are adequately addressed, the manuscript may be considered suitable for publication.

- **Abstract Section:** *"...These seven quarries were found along a line heading approximately 145 degrees...".* In the Introduction (Line 42) and the Discussion, you explicitly state that the quarries align on a **140-degree azimuth**. This should be consistent throughout the entire manuscript to avoid confusing the reader.
- **Abstract Section:** *"...Because the bones were still associated after transport and burial, we can know that the carcasses were still associated during the disarticulation process."*

Using "associated" multiple times in the abstract section. You can consider*"...Because individual bones were still associated after transport and burial, it is evident that carcasses remained intact during the disarticulation process."*

- **Line 127:** *"Neither the maxillaries nor the dentary possessed any teeth."*

You use "maxillaries" (plural noun), but the standard anatomical terms are "maxillae" (plural) and "maxilla" (singular). It should be *"*Neither the maxillae nor the dentary possesses any teeth*".*

- **Line 137:** *"The trend of the 34 elongate bones..."*

Line 100, states that there were 80 elongate specimens in this quarry. Here you mention 34. Clarify right away that these 34 represent the *Triceratops* subset specifically, e.g., *"The trend of the 34 elongate bones attributed to Triceratops..."*

- **Line 158:** *"A hallux (1235.3) was two meters north-northeast of that."*

Did you find a complete, articulated hallux, or a specific hallux phalanx/ungual? Please clarify the element.

- **Line 206:** *"...and clavicle (1234.8) were also recovered."*

Just check these are fused clavicles (**furcula**) or distinct **coracoids**. Given that you already mentioned scapulae, double-check if this element is actually a furcula or a mislabeled coracoid/sternal plate.

- **Line 455:** *"...which would explain the absence of cervical vertebrae from all seven sites."*

Line 129 explicitly states for the *Triceratops* section: *"Two cervical, five dorsal and one caudal vertebrae were recovered..."* Furthermore, line 312 explicitly records a *"syncervical"* at Triceratops5 Quarry. You cannot state cervical vertebrae are absent from *all* seven sites when your own taxonomic descriptions confirm their presence. Just check, which one is correct?

- **Line 482–485:** The identification of a transient shoreline based on quarry alignment is intriguing. If additional sedimentological evidence, such as wave-ripple structures, foreshore deposits, shoreline facies, or associated marine indicators, is available, its inclusion would further strengthen this interpretation. Otherwise, the current explanation is reasonable.
