# Supplementary material for: Evidence for an upper cretaceous strandline deposit in the Lance Formation, Wyoming, USA
Source: PLoS One. 2026 Nov 12;21(11):e0362687. doi: 10.1371/journal.pone.0362687 (PMC13759098; doi:10.1371/journal.pone.0362687)
Supplement: Supplementary file 4 [file pone.0362687.s005.docx]

Dear Prof. Santanu Banerjee,

Thank you very much for inviting me to review the revised manuscript by Dr Keith Snyder et al. submitted to the PLOS One journal.

Manuscript entitled “Evidence for an Upper Cretaceous strandline deposit in the Lance Formation, Wyoming, USA: A strandline in the Lance Formation” (PONE-D-26-22387R1).

The authors have addressed all the previous comments, and the addition of SEM and XRD data has strengthened the interpretations. Once the remaining minor issues, including the incorporation of relevant analytical specifications in the Methodology section, are addressed, the manuscript may be considered suitable for publication.

The authors should provide the relevant analytical specifications in the Methodology section, including the following:

XRD analysis: X-ray radiation source, operating conditions (voltage and current), and scanning range (2θ).

SEM analysis: Imaging mode (secondary electron, SE, or backscattered electron, BSE), accelerating voltage, beam current, and magnification used for image acquisition.

Line number 199: addition of citation to support the glauconite authigenesis.

The authors may consider citing Choudhury et al. (2024), which presents recent studies on glauconite authigenesis and its paleoenvironmental significance. The work of Roy Choudhury et al. (2022) provides valuable insights into glauconite formation and a proxy for interpreting depositional environments and paleoceanographic conditions.

Reference:

Roy Choudhury, T., Khanolkar, S., Banerjee, S., 2022. Glauconite authigenesis during the warm climatic events of Paleogene: Case studies from shallow marine sections of western India. Global and Planetary Change, <https://doi.org/10.1016/j.gloplacha.2022.103857>.

Line number 558-560: addition of citation to support the use of independent paleocurrent indicators for determining sediment transport direction and interpreting sediment distribution patterns.

The authors may consider citing Nahak et al. 2024, which provides a detailed discussion on the application of cross-bedding measurements for reconstructing paleocurrent directions and interpreting sediment dispersal patterns. The study also highlights the relationship between paleodrainage reorganisation and tectonically induced topographic changes, demonstrating how paleocurrent data can be used to infer tectonic controls on sediment transport and basin evolution.

Reference:

Nahak, S.K., Dasgupta, S., Prabhakar, N., and Banerjee, S., 2024. Paleozoic to Mesozoic paleotectonic reconstructions of Gondwana assembly: insights from petrography, heavy mineral chemistry, and detrital U-Th-total Pb monazite geochronology of sandstones in the Pranhita-Godavari Basin (SE India). Marine and Petroleum Geology, p.106914.
