# Supplementary material for: Evidence for an upper cretaceous strandline deposit in the Lance Formation, Wyoming, USA
Source: PLoS One. 2026 Nov 12;21(11):e0362687. doi: 10.1371/journal.pone.0362687 (PMC13759098; doi:10.1371/journal.pone.0362687)
Supplement: Supplementary file 5 [file pone.0362687.s006.docx]

Response to Reviewers

8-17-26

PONE-D-26-22387R1

Evidence for Upper Cretaceous strandline deposit in the Lance Formation, Wyoming, USA

PLOS One

Good afternoon,

We have addressed each and every one of the editor’s comments along with the two reviewers. There is some overlap between the two. In order to help clarify how we addressed each of these suggestions, I have **bolded the suggestion** and followed it with the changes made.

Editor:

**1. Please ensure that your manuscript meets PLOS ONE's style requirements, including those for file naming.**

This has been done. Additionally, all figures have been submitted to NAAS for acceptance. The only figure that we are unsure how to modify is Fig. 7. We have clarified the important points in the figure legend. If anything needs to be done, we are happy to modify it.

**2. In your manuscript, please provide additional information regarding the specimens used in your study. Ensure that you have reported human remain specimen numbers and complete repository information, including museum name and geographic location.**

We have modified the <https://fossil.swau.edu/> link to specify the next step (Quarry). This will allow the reader to get the most up-to-date information for each quarry listed in this manuscript as we find more elements and add them to the database. This is a recognized repository, and has been recognized as the best online repository two years ago. PLOS ONE has accepted it for at least two different publications.

**If no permits were required, please include the following statement:
'No permits were required for the described study, which complied with all relevant regulations.'**

This has been done. Methods: “No permits were required for the described study, which complied with all relevant regulations. Contracts between the landowners and Southwestern Adventist University are on file at the university.”

**3. We noted in your submission details that a portion of your manuscript may have been presented or published elsewhere. Please clarify whether this “Fig. 1 is a modification of Fig. 1 from the PLOS paper "Over 13,000 elements from a single bonebed..."   This paper is a continuation of the research reported on in the above-listed paper. “ was peer-reviewed and formally published. If this work was previously peer-reviewed and published, in the cover letter please provide the reason that this work does not constitute dual publication and should be included in the current manuscript.**

Fig. 1 has been modified from the original publication in PLOS ONE. It is included with this manuscript to help clarify the area that is being studied. We hope to draw readers to the 2020 paper “Over 13,000 elements from a single bonebed…” However, many readers will not access that paper, and need to know where this research is being conducted since strand lines are rarely reported in the paleontological literature.

**4. Thank you for uploading your study's underlying data set. Unfortunately, the repository you have noted in your Data Availability statement does not qualify as an acceptable data repository according to PLOS's standards.**

We have now utilized DRYAD for the data repository. See link below. This link is to be used for review purposes. The final link is listed in the manuscript, but is not active until acceptance.

- [http://datadryad.org/share/LINK_NOT_FOR_PUBLICATION/qyMSbG0TTgzzvjuetzcYovdXdJc8KFayZpdwJhaXITA](https://urldefense.com/v3/__http:/h52wvrds.r.us-west-2.awstrack.me/L0/http:*2F*2Fdatadryad.org*2Fshare*2FLINK_NOT_FOR_PUBLICATION*2FqyMSbG0TTgzzvjuetzcYovdXdJc8KFayZpdwJhaXITA/1/0101019fde06d549-9858fd61-63e2-48b4-b26b-16cac501a676-000000/jygqyiJoKXRLXrrhnCgmA1q6uR4=474__;JSUlJSU!!KM5jTBEYT0g9TeDPtIM!tEfOsC8MKJgSpzvXlUlGyCMI9wwRJ5nExP5JA3NsUkKno8ZEuUTF6OuhHfJGuT9Pc0zUHTWSFkesTHQjUCkLosW0wWhn0HVy$).

**5. We note that Figures 1, 2 and 3 in your submission contain map and satellite images which may be copyrighted.**

We have hired an independent artist to develop Fig. 1. We returned to the field this summer and built our own maps with drone technology (Fig. 2). Fig. 3 was developed in-house using written descriptions of the area of interest from multiple sources including Roberts and Kirschbaum 1995, and Lillegraven and Ostresh 1990. We did not use their figures for this construction.

Additional Editor Comments:

**The paper lacks focuss with too many figures. It needs better organization. You need to address the following comments of the reviewers and revise the paper.**

Agreed. We have incorporated all the major and minor revisions suggested by the reviewers. Please see the comments below. It has greatly strengthened the paper and we are happy to now resubmit.

Reviewers' comments:

**General Comments – Reviewer 1**

**1. The language throughout the manuscript requires significant revision to adhere to the conventions of formal scientific writing. The authors should adopt a more objective, professional tone, minimizing the use of first-person pronouns (e.g., "we," "our") in favor of the passive voice where appropriate. This shift will ensure the focus remains on the methodology and the data rather than the researchers.**

Implementation of this suggestion in combination with the second recommendation below, has resulted in major rewrites of most sections of the manuscript, especially the Introduction, Methods, and Discussion sections. A few examples of these changes are listed below:

**Original:** “Because the bones were still associated after transport and burial, we can know that the carcasses were still associated during the disarticulation process.”

**Modified:** “The preservation of associated remains suggests that soft-tissue connections or integumentary containment persisted during the transport and burial.”

**Original:** “However, our 29 years of research on the Hanson Ranch in Niobrara County in southeastern Wyoming has been focused on ten quarries in a major hadrosaur bonebed in the Lance.”

**Modified:** “Extensive excavation conducted on the Hanson Ranch in Niobrara County, Wyoming over the past three decades has produced tens of thousands of vertebrate fossils …”

**2. The overall structure of the manuscript must be improved to establish a coherent and logical flow. Reorganizing the narrative to better guide the reader systematically through the geological context, taphonomic data, and subsequent paleoenvironmental interpretations will greatly enhance the manuscript's comprehensibility.**

The manuscript has been reorganized to follow this suggested organization. Whereas previously, the Methods section followed this organization, the Results section did not. This has been corrected so that each section of the manuscript follows this suggested flow of information. There are also additional subsection headings to guide the reader, and providing a more coherent flow.

**3. The visual presentation of the data needs substantial streamlining. The current number of figures is excessive and disrupts the flow of the text. The authors must reduce the total figure count by combining related images into structured, multi-panel composite figures (for example, aggregating the individual quarry maps and skeletal diagrams into unified plates). Furthermore, the overall resolution, labeling, and quality of the figures must be upgraded to meet publication standards.**

The original draft separated each of the bone-representations on silhouettes as 9 separate figures. They are now all combined into a single figure (Fig. 9). Triceratops2 Quarry was also represented by 5 separate figures showing bones from individual creatures. We have condensed these down to a single figure representing the entire quarry relying on varying colors for clarification. Due to the additional data requested concerning the confirmation of glauconite inclusion, and newly collected data on paleocurrents, a few additional figures have been added. Total figures have been reduced from 26 to 17. They have been submitted to NAAS for acceptance to PLOS ONE requirements.

**Major Critical Concerns – Reviewer 1**

**1. The authors anchor their interpretation of a marginal marine environment for Cycle 1 entirely on the visual presence of "glauconite grains and glauconitic cement" derived from basic petrographic analysis. Relying solely on visual petrography to characterize these components is insufficient. To properly evaluate the diagenetic overprinting and the origin of these minerals, the study must apply standard methodologies, including Q-XRD (Quantitative X-ray Diffraction) and scanning electron microscopy (SEM) paired with detailed thin-section microscopy. Without these techniques, it is impossible to definitively prove whether the glauconite is authigenic (forming *in situ* and proving marine influence) or reworked/detrital.**

The manuscript has been enhanced with both SEM and XRD figures strengthening the interpretation of a marginal marine environment (Figs. 6,7). Detailed thin-section microscopy is presented in Fig. 5.

**2. The use of double-angle transformation for the bidirectional long-bone data is standard, but the resolution of the 180-degree ambiguity is highly problematic. The authors admit to resolving this ambiguity by using a "known paleocurrent direction, inferred from independent sedimentary structures" to assume a southeast flow. This borders on circular reasoning. If independent sedimentary structures (such as the briefly mentioned trough crossbedding or current ripples) establish the flow, those specific data points must be quantified and presented in their own rose diagrams. The authors cannot use an undocumented assumption to force bidirectional bone data into a unidirectional conclusion.**

A month of work in the field this summer enabled us to collect trough, crossbedding, and climbing ripples (65 samples, Fig. 8, Dryad – see link above). This has strengthened the orientation data that was a weak spot before.

**Original**: “The known paleocurrent direction, inferred from independent sedimentary structures, was toward the southeast.”

**Modified - Methods**: “Also, because axial data give direction but do not independently establish the sense of transport, paleocurrent data from Brunton compass measurements on cross-beds, ripples, and trough axes in the vicinities of the quarries were compiled and used to aid in interpretation of sense of flow (Fig.8, Dryad – see link above).”

**Modified – Discussion**: “Long-bone orientation constitutes axial rather than directional data, and the measured alignments do not independently establish the sense of paleo-flow direction. But the consistent trend throughout the quarries implies the directional data have a sense. In this case, independently derived data obtained from local paleocurrent indicators align with the long-axis data from the bones and give a preferred sense for the bone transport toward the southeast (Fig. 8, [40,41]).”

**3. Connecting seven distinct quarries across a 2.5 km transect without continuous outcrop exposure is a massive interpretive leap. The authors note that continuous stratigraphic tracing is "difficult due to deep weathering, poor outcrop exposure, and limited resolution". Given this lack of continuous control, aligning the sites along a ~140-degree azimuth and concluding they represent a single, contiguous strandline event is highly speculative. The manuscript fails to rule out the possibility that these are simply localized fluvial channel lags or point-bar deposits that happen to align spatially within the broader basin architecture.**

This suggestion has been addressed in the added Discussion portion of the manuscript.

Added – “**Evaluation of Alternative Depositional Models”**

Several depositional models may potentially explain the observed distribution of associated dinosaur remains. Fluvial channel-lag deposits are capable of concentrating vertebrate remains and producing preferred orientation of elongate skeletal elements through hydraulic processes [59-61]. Such deposits commonly exhibit hydraulic sorting, selective preservation of resistant skeletal elements, and extensive disarticulation resulting from transport and reworking [60,61]. Consequently, the repeated occurrence of associated skeletons at multiple localities is more consistent with limited transport and relatively rapid burial than with prolonged accumulation within a reworked channel-lag deposit [61,62].

Point-bar deposits can generate preferred orientation of elongate skeletal elements through hydraulic alignment and sorting [59,63]. However, because the taphonomic characteristics of such deposits are strongly influenced by local hydrologic and depositional conditions, the repeated occurrence of associated skeletons across multiple localities suggests the recurrent operation of similar preservational processes rather than stochastic hydraulic concentration alone [62,64]. Floodplain pond or oxbow-lake settings may preserve associated skeletons but generally do not predict the consistent alignment of multiple quarries along a common regional trend, nor do they readily explain recurring preferred orientations observed across the assemblage.

Deltaic environments remain plausible alternatives. Such settings are characterized by complex interactions among fluvial, tidal, and wave-driven processes [65,66] that can transport, rework, and concentrate vertebrate remains while preserving a broad spectrum of taphonomic states ranging from isolated elements to associated or partially articulated skeletons [67,68]. Each of these models accounts for some aspects of the observed data. However, none individually explains the combined occurrence of associated skeletons, recurring orientation patterns, stratigraphic similarities, and regional spatial alignment as effectively as a shoreline-influenced depositional setting.”

**4. The "bloat and float" model is invoked to explain the missing skulls and cervical vertebrae. However, the manuscript downplays the extreme variance in preservation within Triceratops2 Quarry itself, which contains a tightly associated *Thescelosaurus* alongside highly disarticulated *Anzu* elements. If these carcasses were floating in a marine setting and stranded simultaneously, the authors need to rigorously explain the biomechanical or temporal reasons for such vastly different states of decay upon deposition.**

The modified manuscript addresses this concern in the Discussion.

**Original**: “Degrees of Disarticulation

In Triceratops2 Quarry, a variety of different modes of association of bones were encountered, from completely disarticulated and only loosely associated (*Anzu*), to completely disarticulated, but generally associated (*Triceratops*), to mostly disarticulated, but tightly associated (*Thescelosaurus*), to significantly articulated and associated (*Edmontosaurus*). Although this wide variation in states is informative, it almost necessitates a somewhat different history for each specimen. However, differering amounts of decomposition due to varying time since death could result in slightly different distribution patterns with bones in the more intact carcass, (Thescelosaurus), remaining together and the more decomposed carcasses spreading out due to wave action. A shoreline irregularity could explain the rich accumulation of carcasses in one place, and result in the wider range of bone orientation for this particular quarry. The overall pattern of alignment of bones in the quarry suggests transport of some sort is a part of the explanation, but what mechanism of transport can keep the bones of each taxon together during transport?”

**Modified**: “Carcass Transport and Differential Preservation

One of the most evident features of the assemblage is the substantial variation in preservation among taxa and individuals. Triceratops2 Quarry contains a tightly clustered and relatively complete *Thescelosaurus* skeleton alongside considerably more dispersed remains of *Anzu*, partially associated remains of *Triceratops* and a partially articulated and associated *Edmontosaurus*. This variability suggests that the carcasses entered the depositional system in different states of decay. Differences in body size, buoyancy, transport distances and stages of decomposition all may have been operative in determining the state of preservation of the remains. Experimental studies of vertebrate decomposition demonstrate that carcasses may remain intact for different amounts of time depending on the environmental conditions and biological factors [27]. Thus, the variation in preservation seen in Triceratops2 Quarry does not in itself require disparate depositional environments. In fact, it seems clear that the various carcasses entered a common depositional setting at different stages of decay. Although this wide variation in states is informative, it almost necessitates a somewhat different history for each specimen.

Decomposition due to varying time since death could also explain different distribution patterns seen in the quarry with bones in the most intact carcass (*Thescelosaurus*) remaining together and the more decomposed carcasses spreading out due to wave action and longshore drift. A shoreline irregularity could explain the rich accumulation of carcasses in one place, and result in the wider range of bone orientation for this particular quarry. The overall pattern of alignment of bones in the quarry suggests transport of some sort is a part of the explanation.

Several authors have proposed bloating and floating for dinosaurs found in marine deposits [28-30] and for the frequent discoveries of upside-down ankylosaurs [31,32]. Bloating and floating has been observed in many extant marine vertebrates, with the mandibles and head detaching from the rest of the carcass [33]. Schäfer [33] found from experiments with seal decomposition that the lower jaws detached after 36 days of floating at the surface, and by 41 days “the carcass is still afloat but it now resembles a loose sack” (p. 33). After 48 days, Schäfer [33] noted that the body was open at both ends, but that the vertebral column, rib cage, shoulder blades, pelvis, and thighs were still in the floating sack of skin. Brand et al. [34] with lizards, and Syme and Salisbury [35] with crocodiles, have shown that modern reptiles, allowed to decay in water, are also surrounded by a tough sack of skin that can retain the bones of the creature for extended periods after death. Although dinosaurs like *Triceratops* and *Edmontosaurus* are orders of magnitude larger than lizards, the similar decay in floating seals and extant reptiles suggests that encapsulation and transport of bones in a sack of skin is a realistic possibility to explain some of the taphonomy encountered. Crocodiles [35], lizards [34], and seals [33] initially sank to the bottom after death, then rose and floated at the surface for a protracted period before coming to final rest on the bottom. During the interim, the floating sac of dermis/epidermis tended to keep the bones together as disarticulation ensued. Since both Brand et al. [34] and Syme and Salisbury [35] documented the loss of bones from the dermal/epidermal sac during transport and/or flotation, a similar package of dinosaur remains could be delivered to the site of ultimate burial in a variety of different states, as argued for varying taphonomic modes of dinosaur fossils in marine deposits in Eastern North America [28]. The common occurrence of dinosaur specimens lacking skulls [36] matches well with taphonomic observations of floating carcasses losing heads before elements of the torso [33,34]. The loss of the skull would leave a tear in the dermal sack. This tear may have opened the door to rapid loss of cervical vertebrae in floating dinosaur carcasses, which would explain the absence of most cervical vertebrae from all seven sites. The absence of caudal vertebrae from all sites except for the mostly intact *Edmontosaurus* tail found in Triceratops2 and a few proximal caudals from the *Edmontosaurus* at Atullah2 Quarry, can be explained by the early loss of tails prior to stranding and burial.”

**Reviewer 2.**

**Abstract Section: *"...These seven quarries were found along a line heading approximately 145 degrees...".* In the Introduction (Line 42) and the Discussion, you explicitly state that the quarries align on a 140-degree azimuth. This should be consistent throughout the entire manuscript to avoid confusing the reader.**

This has been corrected to both state 140-degree azimuth.

**Abstract Section: *"...Because the bones were still associated after transport and burial, we can know that the carcasses were still associated during the disarticulation process. "*Using "associated" multiple times in the abstract section. You can consider*"...Because individual bones were still associated after transport and burial, it is evident that carcasses remained intact during the disarticulation process."***

**Modified:** “The preservation of associated remains suggests that soft-tissue connections or integumentary containment persisted during the transport and burial.”

**Line 127: *"Neither the maxillaries nor the dentary possessed any teeth."***

**You use "maxillaries" (plural noun), but the standard anatomical terms are "maxillae" (plural) and "maxilla" (singular). It should be *"*Neither the maxillae nor the dentary possesses any teeth*".***

**Corrected**: Neither the maxillae nor the dentary possessed any teeth.”

**Line 137: *"The trend of the 34 elongate bones..."***

**Line 100, states that there were 80 elongate specimens in this quarry. Here you mention 34. Clarify right away that these 34 represent the *Triceratops* subset specifically, e.g., *"The trend of the 34 elongate bones attributed to Triceratops..."***

**Corrected**: “The trend of the 34 elongate bones attributable to *Triceratops* is generally along a west-northwest to east- southeast line (Fig 5B).

**Line 158: *"A hallux (1235.3) was two meters north-northeast of that."***

**Did you find a complete, articulated hallux, or a specific hallux phalanx/ungual? Please clarify the element.**

**Clarified**: “A phalanx of the hallux (1235.3) was two meters north-northeast of that.”

**Line 206: *"...and clavicle (1234.8) were also recovered."***

**Just check these are fused clavicles (furcula) or distinct coracoids. Given that you already mentioned scapulae, double-check if this element is actually a furcula or a mislabeled coracoid/sternal plate.**

**Clarified**: “Two scapulae (both 1234.8), a single humerus (1235.2), radius (1235.5), and coracoid (1234.8) were also recovered.”

**Line 455: *"...which would explain the absence of cervical vertebrae from all seven sites."* Line 129 explicitly states for the *Triceratops* section: *"Two cervical, five dorsal and one caudal vertebrae were recovered..."* Furthermore, line 312 explicitly records a *"syncervical"* at Triceratops5 Quarry. You cannot state cervical vertebrae are absent from *all* seven sites when your own taxonomic descriptions confirm their presence. Just check, which one is correct?**

**Clarified**: “This tear may have opened the door to rapid loss of cervical vertebrae in floating dinosaur carcasses, which would explain the absence of most cervical vertebrae from all seven sites.”

**Line 482–485: The identification of a transient shoreline based on quarry alignment is intriguing. If additional sedimentological evidence, such as wave-ripple structures, foreshore deposits, shoreline facies, or associated marine indicators, is available, its inclusion would further strengthen this interpretation. Otherwise, the current explanation is reasonable.**

**Additional data collected** in the field:

**Methods:** “Also, because axial data give direction but do not independently establish the sense of transport, paleocurrent data from Brunton compass measurements on cross-beds, ripples, and trough axes in the vicinities of the quarries were compiled and used to aid in interpretation of sense of flow.”

**Results**: “Compiled paleocurrent data from 65 measurements on outcrops in the vicinity of these quarries yielded a mean direction of 141 degrees (r>22, p<.005) shown in Figure 17.”

Thank you for your time and consideration,

Keith Snyder
