# Supplementary material for: Evidence for an upper cretaceous strandline deposit in the Lance Formation, Wyoming, USA
Source: PLoS One. 2026 Nov 12;21(11):e0362687. doi: 10.1371/journal.pone.0362687 (PMC13759098; doi:10.1371/journal.pone.0362687)
Supplement: Peer review history

# Peer review: Decision Letter 0

**DOI:** 10.1371/journal.pone.0362687.r001

**Authors:** Santanu Banerjee

5 Jun 2026

Dear Dr. Snyder,

Thank you for submitting your manuscript to PLOS ONE. After careful consideration, we feel that it has merit but does not fully meet PLOS ONE’s publication criteria as it currently stands. Therefore, we invite you to submit a revised version of the manuscript that addresses the points raised during the review process.

Please submit your revised manuscript by Aug 04 2026 11:59PM. If you will need more time than this to complete your revisions, please reply to this message or contact the journal office at plosone@plos.org. When you're ready to submit your revision, log on to https://www.editorialmanager.com/pone/ and select the 'Submissions Needing Revision' folder to locate your manuscript file.

- A letter that responds to each point raised by the academic editor and reviewer(s). You should upload this letter as a separate file labeled 'Response to Reviewers'.
- A marked-up copy of your manuscript that highlights changes made to the original version. You should upload this as a separate file labeled 'Revised Manuscript with Track Changes'.
- An unmarked version of your revised paper without tracked changes. You should upload this as a separate file labeled 'Manuscript'.

If you would like to make changes to your financial disclosure, please include your updated statement in your cover letter. Guidelines for resubmitting your figure files are available below the reviewer comments at the end of this letter.

If applicable, we recommend that you deposit your laboratory protocols in protocols.io to enhance the reproducibility of your results. Protocols.io assigns your protocol its own identifier (DOI) so that it can be cited independently in the future. For instructions see: https://journals.plos.org/plosone/s/submission-guidelines#loc-laboratory-protocols. Additionally, PLOS ONE offers an option for publishing peer-reviewed Lab Protocol articles, which describe protocols hosted on protocols.io. Read more information on sharing protocols at https://plos.org/protocols?utm_medium=editorial-email&utm_source=authorletters&utm_campaign=protocols.

As the corresponding author, your ORCID iD is verified in the submission system and will appear in the published article. PLOS supports the use of ORCID, and we encourage all coauthors to register for an ORCID iD and use it as well. Please encourage your coauthors to verify their ORCID iD within the submission system before final acceptance, as unverified ORCID iDs will not appear in the published article. *Only*  the individual author can complete the verification step; PLOS staff *cannot*  verify ORCID iDs on behalf of authors.

We look forward to receiving your revised manuscript.

Kind regards,

Santanu Banerjee

Academic Editor

PLOS One

Journal Requirements:

When submitting your revision, we need you to address these additional requirements.

1. Please ensure that your manuscript meets PLOS ONE's style requirements, including those for file naming. The PLOS ONE style templates can be found at

https://journals.plos.org/plosone/s/file?id=wjVg/PLOSOne_formatting_sample_main_body.pdf and

https://journals.plos.org/plosone/s/file?id=ba62/PLOSOne_formatting_sample_title_authors_affiliations.pdf

2. In your manuscript, please provide additional information regarding the specimens used in your study. Ensure that you have reported human remain specimen numbers and complete repository information, including museum name and geographic location.

If permits were required, please ensure that you have provided details for all permits that were obtained, including the full name of the issuing authority, and add the following statement:

'All necessary permits were obtained for the described study, which complied with all relevant regulations.'

If no permits were required, please include the following statement:

'No permits were required for the described study, which complied with all relevant regulations.'

For more information on PLOS One's requirements for paleontology and archeology research, see https://journals.plos.org/plosone/s/submission-guidelines#loc-paleontology-and-archaeology-research.

3. We noted in your submission details that a portion of your manuscript may have been presented or published elsewhere. Please clarify whether this “Fig. 1 is a modification of Fig. 1 from the PLOS paper "Over 13,000 elements from a single bonebed..."   This paper is a continuation of the research reported on in the above-listed paper. “ was peer-reviewed and formally published. If this work was previously peer-reviewed and published, in the cover letter please provide the reason that this work does not constitute dual publication and should be included in the current manuscript.

4. Thank you for uploading your study's underlying data set. Unfortunately, the repository you have noted in your Data Availability statement does not qualify as an acceptable data repository according to PLOS's standards.

At this time, please upload the minimal data set necessary to replicate your study's findings to a stable, public repository (such as figshare or Dryad) and provide us with the relevant URLs, DOIs, or accession numbers that may be used to access these data. For a list of recommended repositories and additional information on PLOS standards for data deposition, please see https://journals.plos.org/plosone/s/recommended-repositories.

5. We note that Figures 1, 2 and 3 in your submission contain map and satellite images which may be copyrighted. All PLOS content is published under the Creative Commons Attribution License (CC BY 4.0), which means that the manuscript, images, and Supporting Information files will be freely available online, and any third party is permitted to access, download, copy, distribute, and use these materials in any way, even commercially, with proper attribution. For these reasons, we cannot publish previously copyrighted maps or satellite images created using proprietary data, such as Google software (Google Maps, Street View, and Earth). For more information, see our copyright guidelines: http://journals.plos.org/plosone/s/licenses-and-copyright.

We require you to either (1) present written permission from the copyright holder to publish these figures specifically under the CC BY 4.0 license, or (2) remove the figures from your submission:

1. You may seek permission from the original copyright holder of Figure(s) [#] to publish the content specifically under the CC BY 4.0 license.

We recommend that you contact the original copyright holder with the Content Permission Form (http://journals.plos.org/plosone/s/file?id=7c09/content-permission-form.pdf) and the following text:

“I request permission for the open-access journal PLOS ONE to publish XXX under the Creative Commons Attribution License (CCAL) CC BY 4.0 (http://creativecommons.org/licenses/by/4.0/). Please be aware that this license allows unrestricted use and distribution, even commercially, by third parties. Please reply and provide explicit written permission to publish XXX under a CC BY license and complete the attached form.”

Please upload the completed Content Permission Form or other proof of granted permissions as an "Other" file with your submission.

In the figure caption of the copyrighted figure, please include the following text: “Reprinted from [ref] under a CC BY license, with permission from [name of publisher], original copyright [original copyright year].”

2. If you are unable to obtain permission from the original copyright holder to publish these figures under the CC BY 4.0 license or if the copyright holder’s requirements are incompatible with the CC BY 4.0 license, please either i) remove the figure or ii) supply a replacement figure that complies with the CC BY 4.0 license. Please check copyright information on all replacement figures and update the figure caption with source information. If applicable, please specify in the figure caption text when a figure is similar but not identical to the original image and is therefore for illustrative purposes only.

The following resources for replacing copyrighted map figures may be helpful:

USGS National Map Viewer (public domain): http://viewer.nationalmap.gov/viewer/

The Gateway to Astronaut Photography of Earth (public domain): http://eol.jsc.nasa.gov/sseop/clickmap/

Maps at the CIA (public domain): https://www.cia.gov/library/publications/the-world-factbook/index.html and https://www.cia.gov/library/publications/cia-maps-publications/index.html

NASA Earth Observatory (public domain): http://earthobservatory.nasa.gov/

Landsat: http://landsat.visibleearth.nasa.gov/

USGS EROS (Earth Resources Observatory and Science (EROS) Center) (public domain): http://eros.usgs.gov/#

Natural Earth (public domain): http://www.naturalearthdata.com/

6. If the reviewer comments include a recommendation to cite specific previously published works, please review and evaluate these publications to determine whether they are relevant and should be cited. There is no requirement to cite these works unless the editor has indicated otherwise.

Additional Editor Comments:

The paper lacks focuss with too many figures. It needs better organization. You need to address the following comments of the reviewers and revise the paper.

[Note: HTML markup is below. Please do not edit.]

Reviewers' comments:

Reviewer's Responses to Questions

**Comments to the Author**

1. Is the manuscript technically sound, and do the data support the conclusions?

Reviewer #1: No

Reviewer #2: Yes

**********

2. Has the statistical analysis been performed appropriately and rigorously?

Reviewer #1: I Don't Know

Reviewer #2: Yes

**********

3. Have the authors made all data underlying the findings in their manuscript fully available?

The PLOS Data policy

Reviewer #1: No

Reviewer #2: Yes

**********

4. Is the manuscript presented in an intelligible fashion and written in standard English?

Reviewer #1: No

Reviewer #2: Yes

**********

Reviewer #1: General Comments

1.The language throughout the manuscript requires significant revision to adhere to the conventions of formal scientific writing. The authors should adopt a more objective, professional tone, minimizing the use of first-person pronouns (e.g., "we," "our") in favor of the passive voice where appropriate. This shift will ensure the focus remains on the methodology and the data rather than the researchers.

2.The overall structure of the manuscript must be improved to establish a coherent and logical flow. Reorganizing the narrative to better guide the reader systematically through the geological context, taphonomic data, and subsequent paleoenvironmental interpretations will greatly enhance the manuscript's comprehensibility.

3.The visual presentation of the data needs substantial streamlining. The current number of figures is excessive and disrupts the flow of the text. The authors must reduce the total figure count by combining related images into structured, multi-panel composite figures (for example, aggregating the individual quarry maps and skeletal diagrams into unified plates). Furthermore, the overall resolution, labeling, and quality of the figures must be upgraded to meet publication standards.

Major Critical Concerns

1.The authors anchor their interpretation of a marginal marine environment for Cycle 1 entirely on the visual presence of "glauconite grains and glauconitic cement" derived from basic petrographic analysis. Relying solely on visual petrography to characterize these components is insufficient. To properly evaluate the diagenetic overprinting and the origin of these minerals, the study must apply standard methodologies, including Q-XRD (Quantitative X-ray Diffraction) and scanning electron microscopy (SEM) paired with detailed thin-section microscopy. Without these techniques, it is impossible to definitively prove whether the glauconite is authigenic (forming in situ and proving marine influence) or reworked/detrital.

2.The use of double-angle transformation for the bidirectional long-bone data is standard, but the resolution of the 180-degree ambiguity is highly problematic. The authors admit to resolving this ambiguity by using a "known paleocurrent direction, inferred from independent sedimentary structures" to assume a southeast flow. This borders on circular reasoning. If independent sedimentary structures (such as the briefly mentioned trough crossbedding or current ripples) establish the flow, those specific data points must be quantified and presented in their own rose diagrams. The authors cannot use an undocumented assumption to force bidirectional bone data into a unidirectional conclusion.

3.Connecting seven distinct quarries across a 2.5 km transect without continuous outcrop exposure is a massive interpretive leap. The authors note that continuous stratigraphic tracing is "difficult due to deep weathering, poor outcrop exposure, and limited resolution". Given this lack of continuous control, aligning the sites along a ~140-degree azimuth and concluding they represent a single, contiguous strandline event is highly speculative. The manuscript fails to rule out the possibility that these are simply localized fluvial channel lags or point-bar deposits that happen to align spatially within the broader basin architecture.

4.The "bloat and float" model is invoked to explain the missing skulls and cervical vertebrae. However, the manuscript downplays the extreme variance in preservation within Triceratops2 Quarry itself, which contains a tightly associated Thescelosaurus alongside highly disarticulated Anzu elements. If these carcasses were floating in a marine setting and stranded simultaneously, the authors need to rigorously explain the biomechanical or temporal reasons for such vastly different states of decay upon deposition.

These required revisions are substantial and are intended to elevate the analytical rigor of the manuscript. The dataset compiled from these Lance Formation quarries clearly represents a massive undertaking and a valuable contribution to Maastrichtian taphonomic studies. By integrating standard analytical methodologies and firmly grounding the stratigraphic correlations, the resulting paleoenvironmental interpretations will be significantly stronger and more impactful for the broader paleoecological community. I suggest authors resubmit the manuscript after addressing the suggested concerns.

Reviewer #2: •Abstract Section: "...These seven quarries were found along a line heading approximately 145 degrees...". In the Introduction (Line 42) and the Discussion, you explicitly state that the quarries align on a 140-degree azimuth. This should be consistent throughout the entire manuscript to avoid confusing the reader.

•Abstract Section: "...Because the bones were still associated after transport and burial, we can know that the carcasses were still associated during the disarticulation process."

Using "associated" multiple times in the abstract section. You can consider"...Because individual bones were still associated after transport and burial, it is evident that carcasses remained intact during the disarticulation process."

•Line 127: "Neither the maxillaries nor the dentary possessed any teeth."

You use "maxillaries" (plural noun), but the standard anatomical terms are "maxillae" (plural) and "maxilla" (singular). It should be "Neither the maxillae nor the dentary possesses any teeth".

•Line 137: "The trend of the 34 elongate bones..."

Line 100, states that there were 80 elongate specimens in this quarry. Here you mention 34. Clarify right away that these 34 represent the Triceratops subset specifically, e.g., "The trend of the 34 elongate bones attributed to Triceratops..."

•Line 158: "A hallux (1235.3) was two meters north-northeast of that."

Did you find a complete, articulated hallux, or a specific hallux phalanx/ungual? Please clarify the element.

•Line 206: "...and clavicle (1234.8) were also recovered."

Just check these are fused clavicles (furcula) or distinct coracoids. Given that you already mentioned scapulae, double-check if this element is actually a furcula or a mislabeled coracoid/sternal plate.

•Line 455: "...which would explain the absence of cervical vertebrae from all seven sites."

Line 129 explicitly states for the Triceratops section: "Two cervical, five dorsal and one caudal vertebrae were recovered..." Furthermore, line 312 explicitly records a "syncervical" at Triceratops5 Quarry. You cannot state cervical vertebrae are absent from all seven sites when your own taxonomic descriptions confirm their presence. Just check, which one is correct?

•Line 482–485: The identification of a transient shoreline based on quarry alignment is intriguing. If additional sedimentological evidence, such as wave-ripple structures, foreshore deposits, shoreline facies, or associated marine indicators, is available, its inclusion would further strengthen this interpretation. Otherwise, the current explanation is reasonable.

**********

what does this mean?). If published, this will include your full peer review and any attached files.

If you choose “no”, your identity will remain anonymous but your review may still be made public.

**Do you want your identity to be public for this peer review?**  For information about this choice, including consent withdrawal, please see our Privacy Policy

Reviewer #1: No

Reviewer #2: **Yes:**  Dr. Sankar Kumar Nahak

**********

[NOTE: If reviewer comments were submitted as an attachment file, they will be attached to this email and accessible via the submission site. Please log into your account, locate the manuscript record, and check for the action link "View Attachments". If this link does not appear, there are no attachment files.]

To ensure your figures meet our technical requirements, please review our figure guidelines: https://journals.plos.org/plosone/s/figures

You may also use PLOS’s free figure tool, NAAS, to help you prepare publication quality figures: https://journals.plos.org/plosone/s/figures#loc-tools-for-figure-preparation.

NAAS will assess whether your figures meet our technical requirements by comparing each figure against our figure specifications.

Attachment Submitted filename: Reviewer comments.docx

Attachment Submitted filename: PONE-D-26-22387_Comment to author.pdf

Attachment Submitted filename: Comment to Author.docx

# Author comment: Author response to Decision Letter 1

**DOI:** 10.1371/journal.pone.0362687.r002

4 Sep 2026

The point by point response to the editor and two reviewers has been uploaded as directed into the "Response to Reviewers".

Attachment Submitted filename: Response to Reviewers - submit 8-17-26.docx

# Peer review: Decision Letter 1

**DOI:** 10.1371/journal.pone.0362687.r003

**Authors:** Santanu Banerjee

29 Oct 2026

Evidence for an Upper Cretaceous strandline deposit in the Lance Formation, Wyoming, USA

PONE-D-26-22387R1

Dear Dr. Snyder,

We’re pleased to inform you that your manuscript has been judged scientifically suitable for publication and will be formally accepted for publication once it meets all outstanding technical requirements.

Within one week, you’ll receive an e-mail detailing the required amendments. When these have been addressed, you’ll receive a formal acceptance letter and your manuscript will be scheduled for publication.

An invoice will be generated when your article is formally accepted. Please note, if your institution has a publishing partnership with PLOS and your article meets the relevant criteria, all or part of your publication costs will be covered. Please make sure your user information is up-to-date by logging into Editorial Manager at Editorial Manager® and clicking the ‘Update My Information' link at the top of the page. For questions related to billing, please contact billing support.

If your institution or institutions have a press office, please notify them about your upcoming paper to help maximize its impact. If they’ll be preparing press materials, please inform our press team as soon as possible -- no later than 48 hours after receiving the formal acceptance. Your manuscript will remain under strict press embargo until 2 pm Eastern Time on the date of publication. For more information, please contact onepress@plos.org.

Kind regards,

Santanu Banerjee

Academic Editor

PLOS One

Additional Editor Comments (optional):

Reviewers' comments:

Reviewer's Responses to Questions

**Comments to the Author**

Reviewer #2: All comments have been addressed

**********

2. Is the manuscript technically sound, and do the data support the conclusions?

Reviewer #2: Yes

**********

3. Has the statistical analysis been performed appropriately and rigorously?

Reviewer #2: Yes

**********

4. Have the authors made all data underlying the findings in their manuscript fully available?

The PLOS Data policy

Reviewer #2: Yes

**********

5. Is the manuscript presented in an intelligible fashion and written in standard English?

Reviewer #2: Yes

**********

Reviewer #2: (No Response)

**********

what does this mean?). If published, this will include your full peer review and any attached files.

If you choose “no”, your identity will remain anonymous but your review may still be made public.

**Do you want your identity to be public for this peer review?**  For information about this choice, including consent withdrawal, please see our Privacy Policy

Reviewer #2: No

**********

Attachment Submitted filename: Comments to author.docx

# Editor report: Acceptance letter

**DOI:** 10.1371/journal.pone.0362687.r004

**Authors:** Santanu Banerjee

PONE-D-26-22387R1

PLOS One

Dear Dr. Snyder,

I'm pleased to inform you that your manuscript has been deemed suitable for publication in PLOS One. Congratulations! Your manuscript is now being handed over to our production team.

At this stage, our production department will prepare your paper for publication. This includes ensuring the following:

* All references, tables, and figures are properly cited

* All relevant supporting information is included in the manuscript submission,

* There are no issues that prevent the paper from being properly typeset

You will receive further instructions from the production team, including instructions on how to review your proof when it is ready. Please keep in mind that we are working through a large volume of accepted articles, so please give us a few days to review your paper and let you know the next and final steps.

Lastly, if your institution or institutions have a press office, please let them know about your upcoming paper now to help maximize its impact. If they'll be preparing press materials, please inform our press team within the next 48 hours. Your manuscript will remain under strict press embargo until 2 pm Eastern Time on the date of publication. For more information, please contact onepress@plos.org.

You will receive an invoice from PLOS for your publication fee after your manuscript has reached the completed accept phase. If you receive an email requesting payment before acceptance or for any other service, this may be a phishing scheme. Learn how to identify phishing emails and protect your accounts at https://explore.plos.org/phishing.

If we can help with anything else, please email us at customercare@plos.org.

Thank you for submitting your work to PLOS One and supporting open access.

Kind regards,

PLOS One Editorial Office Staff

on behalf of

Dr. Santanu Banerjee

Academic Editor

PLOS One
